# Supplementary material for: Honour, competition and cooperation across 13 societies
Source: Nat Hum Behav. 2025 Sep 26;10(2):255–67. doi: 10.1038/s41562-025-02308-0 (PMC12932108; doi:10.1038/s41562-025-02308-0)
Supplement: Supplementary file 1 — Supplementary Figs. 1–7b, Tables 1–42 and analyses. [file 41562_2025_2308_MOESM1_ESM.pdf]

nature human behaviour

Supplementary information https://doi.org/10.1038/s41562-025-02308-0

Honour, competition and cooperation across 13 societies

In the format provided by the authors and unedited

Table of Contents

1. Preregistration Deviations and Unregistered Steps **2**

2. Obtaining Factor Scores for Robustness Checks **8**

2.1 Perceived normative and personal honour values 9

2.2 Belief in a zero-sum game 10

2.3 Relational mobility 10

3. Supporting Analyses **12**

3.1 Competition and cooperation 12

3.2 Honour and competition 15

3.3 Honour and cooperation 36

3.4 Honour and anticipation of coordination success 65

3.5 Honour and behavioural deviations from expectations 68

4. Descriptives **74**

5. Games **85**

5.1 Contest game 85

5.2 Step-level public goods game 87

6. References **89**

1. Preregistration Deviations and Unregistered Steps

Table S1. Preregistration deviations

#	Details		Original Wording	Deviation Description	Reader Impact
1	Type	Covariates; Analysis	*"Covariates such as number of rounds, gender and age will be entered to the aforementioned models as controls."*	We were unable to retrieve information about the number (i.e., order) of the randomized rounds from the Qualtrics survey due to programming constraints and could not include this variable as a control as pre-registered. Instead, variables including the order of the game and gender information of the partner were included in the model as controls.	We did not expect this deviation affecting our hypothesis testing. The inclusion of other controls, such as the order of the game and the partner's gender, ensured that key contextual variables were still accounted for in the analysis.
	Reason	Plan not possible			
	Timing	After data access			

Note. The present study has been pre-registered on OSF (https://osf.io/r9atc) before data collection. We reported preregistration deviations following the guide by Willroth and Atherton[1].

Table S2. Unregistered steps

#		Details	Original Wording	Deviation Description	Reader Impact
1	Type	Variables; Analysis	We did not pre-register the exclusion criteria.	We applied four exclusion criteria: (a) 120 participants who were not born in and currently located in the respective society; (b) 24 participants who did not self-identify as male or female. Participants were given a third gender option at the beginning of the study, and all were allowed to complete the survey and receive full compensation, including any game earnings. However, for data analysis, we only included participants who identified as male or female. This decision was made to align with our experimental design, which manipulated the gender of the game partner using binary categories (male, female, or gender not provided) to explore specific dynamics in competition and cooperation among female–female, male–male, and mixed-gender pairs. Given the scope of this manuscript, analyses of gender effects will be addressed in a separate paper; (c) 29 participants who failed the attention check question; and (d) 112 participants who failed all four comprehension questions designed to assess understanding of the contest game and step-level public goods game (PGG) rules.	This unregistered step was taken to improve the reliability of the data and the precision of the estimated associations between cultural variables and behavioural outcomes. Specifically, criterion (a) ensured that participants were embedded in the relevant cultural context; criterion (b) maintained alignment between the sample and the experimental manipulation; and criteria (c) and (d) ensured that participants were attentive and meaningfully engaged with the study.
	Timing	Before data access; During data collection			

Table S2 (continued)

#	Details		Original Wording	Deviation Description	Reader Impact
2	Type	Variables; Analysis	In the pre-registration, we did not specify whether we would use observed scores or factor scores of the predictor variables of interest (e.g., personal and perceived normative honour values, beliefs in a zero-sum game, relational mobility).	We used observed scores (e.g., unweighted means of scale items at the individual level, society means at the societal level) for hypothesis testing and exploratory analyses, while consistently using factor scores for robustness checks of these analyses. We conducted multilevel confirmatory factor analyses (CFA) to adjust for response style and obtain factor scores at both between-society level and within-society level for subsequent analyses in mixed-effects regression models (see Supplementary Section 2 for more information).	This unregistered step provided a more thorough test of the hypotheses and exploratory analyses, and also ensured the robustness of our findings.
	Timing	After data access			
3	Type	Variables; Research Q(s); Analysis	In the pre-registration, we specified that we would generate a societal-level honour using data from individual-level perceived normative honour values. However, for individual-level analyses, we did not specify whether we would use an overall indicator of perceived normative or personal honour values across both facets of honour values, or separate indicators for each facet (i.e., self-promotion and retaliation, defence of family reputation) to predict outcome variables (e.g., competition, cooperation).	For individual-level analyses, we calculated two separate indicators for each facet of perceived normative honour values as well as of personal honour values. These four individual-level indicators for perceived normative and personal honour values were then entered as predictors to the same mixed-effects models for hypotheses testing and additional analyses.	This unregistered step allowed for a more precise analysis of the relationships between perceived normative (and personal) honour values and the outcome variables. This approach prevented the dilution of specific associations that might occur with an overall score, enabling a clearer understanding of how each facet uniquely explained variations in behaviours and expectations.
	Timing	After data access			

Table S2 (continued)

#	Details		Original Wording	Deviation Description	Reader Impact
4	Type	Variables; Research Q(s); Analysis	In the pre-registration, we specified that we would test both societal and individual-level effects of honour values, with societal-level honour indicator obtained from individual-level perceived normative honour values. However, we did not pre-register the exploration of contextual effects of societal-level honour on competition, cooperation or expectations of these behaviours from unrelated others.	As an additional exploratory analysis, we applied a grand mean centring approach to the observed scores of perceived normative honour values to further decompose the between-society effects into contextual and individual-level effects. Specifically, we added societal mean perceived normative values and grand mean-centred individual perceived normative honour values (along with control variables such as participant age and gender, partner gender information, and game order) into the same model. This allowed us to examine the fixed effect of societal-level perceived normative honour values on behaviours or expectations, while controlling for individual-level perceived normative honour values — the contextual effect (see Supplementary Sections 3.2.4 and 3.3.4 for more information).	These unregistered exploratory analyses allowed us to explore unique questions about how (hypothetical) individuals with the same level of perceived normative honour values, but inhabiting in societies with different societal mean perceived normative honour values, would differ in competition, cooperation, or expectations of these behaviours from unrelated others. These findings complemented the results from our pre-registered hypotheses and research questions regarding between-society and within-society effects.
	Timing	After data access			

Table S2 (continued)

#	Details		Original Wording	Deviation Description	Reader Impact
5	Type	Research Q(s); Analysis	We did not pre-register to explore whether honour values would be associated with coordinative decisions (contributing 8 or 6 MUs), or the occurrence of different types of anticipated coordination success in the step-level public goods game at the societal and individual levels.	As an additional exploratory analysis, we used generalized linear mixed models to examine the association between societal-level honour (and individual-level personal and perceived normative honour values) and the likelihood of contributing 8 or 6 MUs in a given round (see Supplementary Section 3.4.1 for more information). Using the sum of an individual's own investment and expected partner's investment in a given round, we categorized a given game round in the step-level public goods game as anticipated *efficient coordination* success, *less-efficient coordination* success and *failed coordination* (see Supplementary Section 3.4.3 for more information). We then used generalized linear mixed models to explore the association between societal-level honour or individual-level honour values and the occurrence of anticipated success of efficient coordination or less-efficient coordination (i.e., the likelihood of a game round being categorized as efficient coordination success or less-efficient coordination success).	These unregistered exploratory analyses provided us with novel insights into the association between honour, coordination, and anticipation of coordination dynamics and outcomes with unrelated others in one's society. Given that each contributing 8 MUs is one of the Nash equilibria in the current step-level public goods game (see Supplementary Section 5.2 for more information), these analyses can further reveal how closely individuals aligned their behaviour with rational, self-interested decision-making strategies, and how honour values may relate to adherence to this equilibrium.
	Timing	After data access			

Table S2 (continued)

#	Details		Original Wording	Deviation Description	Reader Impact
6	Type	Research Q(s); Analysis	We did not pre-register to explore the differences between one's own investment and expectations of other's investment, and whether honour values would be associated with different types of behavioural deviations from expectations at both societal and individual levels.	As an additional exploratory analysis, we categorized a given game round in the contest game as *underinvested competition*, *tie*, *efficient competition*, and *less-efficient competition* (see Supplementary Section 3.5.1 for more information), and categorized a given game round in the step-level public goods game as *underinvested cooperation*, *conditional cooperation*, and *unconditional cooperation* (see Supplementary Section 3.5.2 for more information). We then used generalized linear mixed models to explored the association between societal-level honour or individual-level honour values and a) the occurrence of efficient competition or less-efficient competition (i.e., the likelihood of a game round being categorized as efficient competition and less-efficient competition), and b) the occurrence of conditional cooperation or unconditional cooperation (i.e., the likelihood of a game round being categorized as conditional cooperation or unconditional cooperation).	Since the games used in the present study define both competing and cooperating as investing behaviour, it is challenging to distinguish the general tendency to allocate monetary units (and expect others to do so) from the behaviours (and expectations) measured in these two games. This deviation approach helped address this challenge by examining how individuals behave relative to their expectations of their game partner's behaviour. These unregistered exploratory analyses provided us with novel insights into the association between honour and competitive and cooperative behavioural patterns relative to one's expectations of other's behaviours. There findings complemented the results from our pre-registered hypotheses and research questions.
	Timing	After data access			

Note. The present study has been pre-registered on OSF (https://osf.io/r9atc) before data collection. We reported preregistration deviations following the guide by Willroth and Atherton[1].

2. Obtaining Factor Scores for Robustness Checks

As explained in the unregistered steps (see Table S2), we did not specify in the pre-registration whether we will use observed scores or factor scores of the predictor variables of interest (e.g., personal and perceived normative honour values, beliefs in a zero-sum game, relational mobility). To provide robust findings, we conducted analysis using observed scores (e.g., unweighted means of scale items at the individual level, society means at the societal level) and used factor scores as a robustness check. Compared to simply averaging ratings from multiple items, factor scores offer advantages such as weighting items based on their loadings onto the factor and allowing for adjustment of response style. For each of the four sets of items (i.e., personal honour values, perceived normative honour values, relational mobility, belief in a zero-sum game), we conducted multilevel confirmatory factor analysis (CFA) to separately model factors at the within- and between-society levels.

We conducted the analyses using Mplus Version 8.10[2]. We evaluated model fit using the Comparative Fit Index (CFI), Tucker-Lewis Index (TLI), Root Mean Square Error of Approximation (RMSEA), and Standardized Root Mean Squared Residual (SRMR). Values of CFI and TLI > .95 (or > .90) RMSEA < .06 (or < .08), and SRMR < .08 (or < .10) have been proposed as criteria for "good" (or "acceptable") fit[3,4]. For multilevel models, Mplus provides separate values of SRMR for the within-society and between-society parts of the model: $SRMR_{within}$ and $SRMR_{between}$. However, it is known that SRMR becomes inflated and is arguably of limited use with sample sizes below 200[5]. With 13 units of analysis at the between-society level of our multilevel models, we therefore considered that values of $SRMR_{between}$ < .20 should be considered acceptable, provided that other fit indices did not suggest otherwise.

2.1 Perceived normative and personal honour values

For both personal and perceived normative honour values, we adopted a multilevel measurement model based on recent research[6]. This measurement model included one culture-level content factor of honour, and separated honour values into two distinct content factors, (a) defending family reputation and (b) self-promotion and retaliation, at the individual level. To adjust for differences in response style in the measurement model, we created four indicators of acquiescent responding, each defined by averaging a pair of items with opposing substantive content from the relational mobility measure (e.g., averaged agreement with "*It is common for these people to have a conversation with someone they have never met before*" and "*It is uncommon for these people to have a conversation with people they have never met before*" without reverse scoring). We used these items to anchor a method factor which allowed us to adjust our measures of perceived normative values (and personal values) for acquiescent responding both at the individual and cultural level. The model fitted the data well (perceived normative values: $\chi^2_{[161]} = 880.721$, $CFI = .958$, $TLI = .952$, $RMSEA = .036$, $SRMR_{Within} = .114$, $SRMR_{Between} = .145$; personal values: $\chi^2_{[161]} = 898.684$, $CFI = .958$, $TLI = .953$, $RMSEA = .037$, $SRMR_{Within} = .082$, $SRMR_{Between} = .173$). The between-society level content factor of honour values showed significant variance in the multilevel CFA model for both personal ($p = 0.016$) and perceived normative values ($p = 0.020$), indicating cross-societal variation in both personal and perceived normative honour values. Factor scores for societal-level honour values and individual-level honour values, the latter including the dimensions of defence of family reputation, and self-promotion and retaliation, were saved from the final CFA models for personal and perceived normative honour values (see Mplus syntax file "personal_honor_values.out" and "perceived_normative_honor_values.out" on OSF at https://osf.io/3dscw/).

2.2 Belief in a zero-sum game

To obtain factor scores for belief in a zero-sum game[7], we applied a multilevel approach, including two content factors, one at the within-society level and the other at the between-society level. The same approach was used to adjust for differences in response style as was used for honour values, i.e., four indicators of acquiescent responding were created from selected items of the relational mobility measure to anchor method factors at the within- and between-samples level (see 2.1 Personal and perceived normative honour values). Two items ("*When some people are getting poorer, it means that other people are getting richer*", "*The wealth of a few is acquired at the expense of many*") were retained only at the within, but not the between-society level due to negative loadings and these items were therefore centered within societies. The model fitted the data well ($\chi^2_{[96]}$ = 837.868, *CFI* = .946, *TLI* = .937, *RMSEA* = .052, $SRMR_{Within}$ = .057, $SRMR_{Between}$ = .185). However, the between-society level content factor of beliefs in a zero-sum game did not show significant variance in the multilevel CFA model (p = .118), indicating that beliefs in a zero-sum game may not differ much at the societal level among the current samples. Factor scores for societal-level and individual-level beliefs in a zero-sum game were saved from the final CFA model for beliefs in a zero-sum game (see Mplus syntax file "bzsg.out" on OSF at https://osf.io/3dscw/).

2.3 Relational mobility

For relational mobility, we referred to the measurement model demonstrated in a previous study[8], and adopted a multilevel approach to obtain factor scores for relational mobility at the individual and culture-levels. The within-society part of the measurement model therefore included two first-order content factors (i.e., a "meeting" factor capturing the degree to which a society or social context affords opportunities for individuals to meet new people and forge new relationships, and a "choosing" factor capturing the degree to which

people have the freedom to choose and leave relationships based on personal preference) and a second-order content factor (i.e., relational mobility); at the between-society level, we modelled a single content factor representing relational mobility. To adjust for differences in response style, we introduced a method factor at both within and between-samples levels to account for variance due to acquiescent responding; all items had a fixed loading of 1 on this factor. The model fitted the data well ($\chi^2_{[107]}$ = 698.674, *CFI* = .956, *TLI* = .946, *RMSEA* = .041, $SRMR_{Within}$ = .033, $SRMR_{Between}$ = .082). However, the between-society level content factor of relational mobility did not show significant variance in the multilevel CFA model (*p* = .287), indicating that relational mobility may not differ much at the societal level among the current samples. Factor scores for societal-level and individual-level relational mobility, the latter including two first-order content factors (i.e., meeting and choosing) and one second-order content factor (i.e., relational mobility), were saved from the final CFA model for relational mobility (see Mplus syntax file "relational_mobility.out" on OSF at https://osf.io/3dscw/).

3. Supporting Analyses

3.1 Competition and cooperation

3.1.1 Cross-societal variation in competition and cooperation

Table S3. Model comparisons testing cross-societal variation in competition and cooperation.

Model	n_{par}	AIC	BIC	logLik	deviance	Chisq	Df	Pr(>Chisq)
m_CGB_0	3	83571	83595	-41782	83565			
m_CGB_1	4	83541	83573	-41767	83533	31.34	1	< .001
m_CGE_0	3	84535	84558	-42264	84529			
m_CGE_1	4	84497	84529	-42245	84489	39.34	1	< .001
m_SLB_0	3	82373	82397	-41184	82367			
m_SLB_1	4	82335	82367	-41164	82327	39.81	1	< .001
m_SLE_0	3	82602	82626	-41298	82596			
m_SLE_1	4	82567	82599	-41280	82559	37.03	1	< .001

Note. CGB = Competitive behaviour in the contest game, CGE = Expectations about other's competition, SLB = Cooperative behaviour in the step-level public goods game, SLE = Expectations about other's cooperation, n_{par} = number of parameters in the model, AIC = Akaike Information Criterion, BIC = Bayesian Information Criterion, logLik = Log-likelihood, deviance = Deviance of the model, Chisq = Chi-squared statistic for comparing models, Df = Degrees of freedom, Pr(>Chisq) = P-value of the Chi-squared test. m_[###]_0 indicated the intercept only model with participant ID included as a random intercept, m_[###]_1 indicated the intercept only model with participant ID and society included as two random intercepts. Model comparisons were consistently performed by contrasting each m_[###]_1 with each m_[###]_0.

3.1.2 Correlations between competition and cooperation

Table S4. Mixed-effects models with cooperation behaviour (competition behaviour) predicting competition behaviour (cooperation behaviour).

	**Model S4a: Competition**						**Model S4b: Cooperation**					
Predictors	*B*	*SE*	*t*(*df*)	*p*	*std. Beta*	*std. 95% CI*	*B*	*SE*	*t*(*df*)	*p*	*std. Beta*	*std. 95% CI*
(Intercept)	1.258	1.250	1.006(26)	0.336	/	/	1.495	1.213	1.232(23)	0.243	/	/
Societal mean cooperation	**0.791**	**0.200**	**3.946(11)**	**0.002**	**0.106**	**[0.047, 0.165]**						
Individual mean cooperation	**0.654**	**0.016**	**41.508(3354)**	**<0.001**	**0.581**	**[0.553, 0.608]**						
Societal mean competition							**0.750**	**0.189**	**3.969(11)**	**0.002**	**0.116**	**[0.052, 0.180]**
Individual mean competition							**0.519**	**0.012**	**41.510(3354)**	**<0.001**	**0.572**	**[0.545, 0.599]**
Participant gender [male]	0.186	0.056	3.312(3355)	0.001	0.092	[0.037, 0.146]	-0.012	0.050	-0.240(3355)	0.810	-0.007	[-0.061, 0.047]
Age	-0.004	0.002	-1.795(3357)	0.073	-0.025	[-0.052, 0.002]	0.006	0.002	3.270(3357)	0.001	0.045	[0.018, 0.072]
Game order [SL-CG]	0.548	0.057	9.677(3358)	<0.001	0.271	[0.216, 0.325]	-0.602	0.050	-12.026(3357)	<0.001	-0.331	[-0.385, -0.277]
**Random Effects**												
σ^2	2.653						2.103					
τ_{00}	0.028_{Society}						0.029_{Society}					
ICC	0.010						0.013					
N	13_{Society}						13_{Society}					
Observations	3371						3371					
Marginal R^2 / Conditional R^2	0.349 / 0.356						0.358 / 0.367					

Note. Societal mean cooperation = the mean level of cooperation across all participants per society in the step-level public goods game, Individual mean cooperation = the mean level of cooperation per participant across the six decisions in the step-level public goods game (centred within society), Societal mean competition = the mean level of competition across all participants per society in the contest game, Individual mean competition = the mean level of competition per participant across the six decisions in the contest game (centred within society), SL = step-level public goods game, CG = contest game, *std. Beta* = standardized regression coefficient. The reference level for participant gender was [female], and for game order was [CG-SL]. This table and the tables below showed estimates without adjustments for multiple comparisons unless specified. All tests were two-sided. Degree of freedom and p-values were estimated using the Satterthwaite approximation.

Societies	n	r [95%CI]
United States	256	0.74 [0.66 , 0.82]*
Lebanon	250	0.67 [0.58 , 0.77]*
Türkiye	260	0.63 [0.54 , 0.73]*
Egypt	270	0.63 [0.54 , 0.72]*
South Korea	271	0.63 [0.54 , 0.72]*
Spain	249	0.62 [0.52 , 0.72]*
United Kingdom	255	0.58 [0.48 , 0.68]*
Turkish Cypriot	245	0.57 [0.47 , 0.67]*
Japan	261	0.51 [0.40 , 0.61]*
Italy	270	0.51 [0.40 , 0.61]*
Greek Cypriot	269	0.47 [0.36 , 0.57]*
Greece	255	0.44 [0.32 , 0.55]*
Morocco	260	0.37 [0.26 , 0.49]*

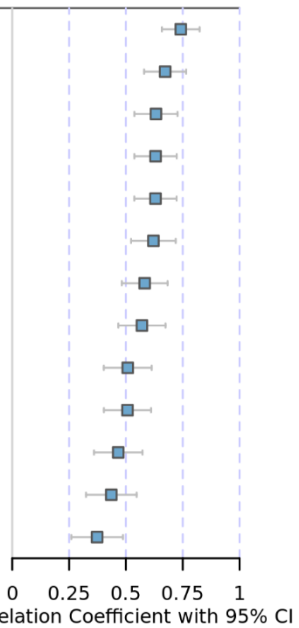

Figure S1. Forest plot of correlations between competitive and cooperative behaviour per society.
Note. n = sample size, r [95%CI] = correlation coefficient [95% confidence interval].
* $p < 0.05$

3.2 Honour and competition

In this section, we present the results of hypothesis testing (Section 3.2.1) and its robustness checks (Section 3.2.2), as well as exploratory analyses (mostly pre-registered) related to honour and competition (Section 3.2.3 to 3.2.6). First, we tested our hypotheses by running models with societal-level and individual-level honour values predicting competition and expectations of other's competition in separate models (Table S5-S6). For societal-level honour, we used a single indicator: the societal mean of perceived normative honour values across two facets — self-promotion and retaliation (SPR) and defence of family reputation (DFR). At the individual level, we derived four honour indicators, including personal values of SPR and DFR, and perceive normative values of SPR and DFR. These individual-level indicators were calculated using unweighted means across the respective scale items (referred to as *observed scores*). Next, we conducted robustness checks by using *factor scores* of honour values to predict competition and expectations of other's competition (Table S7-S8). The rationale for conducting these robustness checks can be found in the unregistered steps outlined in Supplementary Section 1, while details of the factor analysis were provided in Supplementary Section 2.

We also explored potential cross-level interactions, specifically whether societal-level honour would interact with individual-level honour values in predicting competition. This exploratory analysis used both observed scores and factor scores (Table S9). We then explored whether there was contextual effect of societal-level honour on competition and expectations of other's competition, while controlling for individual-level grand mean centred perceived normative honour values (see Table S10). Additionally, we tested whether beliefs in a zero-sum game would account for additional variance in competition beyond honour values at both the societal and individual levels (Table S11-S12). Finally, we explored a set of societal-level indicators that are theoretically relevant to the emergence of

the cultural logic of honour and examined their associations with competition (Table S13-S14).

3.2.1 Honour, competition and expectations about other's competition

In this section, we present the results from models with societal-level honour predicting competition (Model S5a) or expectations of other's competition (Model S6a). Results showed that societal-level honour was positively associated with competition, while no significant association was found with expectations of other's competition. We also fitted an intercept-only model with society and participant as random intercepts (see Model "m_CGB_0" in the online syntax "data_analysis_final.Rmd" on OSF), which showed that the variance of competition at the society level was 0.070. When societal-level honour was added as a predictor, this variance decreased to 0.043 (see Model S5a), suggesting that societal-level honour accounted for 38.57% of the societal-level variance in competition.

We then added four individual-level honour value indicators (centred within society) to the model to test whether personal honour values and perceived normative honour values are associated with competition (Model S5b) or expectations of other's competition (Model S6b). The models controlled for partner gender information, participant gender, age, and order of the game. The generalized variance inflation factor adjusted for the degree of freedom indicated a low risk of multicollinearity in both models [all the $\mathrm{GVIF}^{1/(2\times \mathrm{Df})} < 2$] (see Models "m_CGB_2_vif" and "m_CGE_2_vif" in the online syntax "data_analysis_final.Rmd" on OSF). The model results showed that perceived normative honour values for both facets were positively associated with competition and expectations of other's competition. However, neither the self-promotion and relation nor the defence of family reputation dimensions of personal honour values were associated with competition or expectations of other's competition.

For testing individual-level honour indicators, we conducted robustness checks by adding three additional demographic variables as controls into Models S5b and S6b: parents' education levels (1-8), belongingness to the ethnic majority group in the respective society (no, yes), and living environment (rural, urban, both). There analyses were conducted in Models named "m_CGB_2_RC" and "m_CGE_2_RC" in the online syntax "data_analysis_final.Rmd" on OSF. The results largely replicated findings from Models S5b and S6b. Specifically, perceived normative honour values of self-promotion and retaliation, as well as defence of family reputation, were associated with higher levels of competition [$\beta = .036$, $p = .048$ (SPR), $\beta = .073$, $p <.001$ (DFR), see results from Model object "m_CGB_2_RC"]. However, only perceived normative values of defence of family reputation were positively related to expectations of others' competition [$\beta = .028$, $p = .106$ (SPR); $\beta = .066$, $p = .001$ (DFR), see results from Model object "m_CGE_2_RC"]. Individual-level measures of personal honour values across both facets were not associated with engagement in competitive behaviour (*p*s > .150).

Table S5. Mixed-effects models with societal-level and individual-level honour values (observed scores) predicting individuals' own competitive behaviours.

	**Model S5a: Competition Behaviour**						**Model S5b: Competition Behaviour**					
Predictors	*B*	*SE*	*t(df)*	*p*	*std. Beta*	*std. 95% CI*	*B*	*SE*	*t(df)*	*p*	*std. Beta*	*std. 95% CI*
(Intercept)	4.567	0.720	6.346(11)	<0.001	/	/	4.407	0.728	6.053(27)	<0.001	/	/
Societal-level honour	**0.357**	**0.139**	**2.558(11)**	**0.027**	**0.069**	**[0.010, 0.128]**	0.356	0.139	2.559(11)	0.027	0.069	[0.010, 0.128]
Normative honour (SPR)							**0.100**	**0.039**	**2.589(3351)**	**0.010**	**0.047**	**[0.011, 0.082]**
Normative honour (DFR)							**0.154**	**0.045**	**3.447(3351)**	**0.001**	**0.069**	**[0.030, 0.109]**
Personal honour (SPR)							**-0.051**	**0.035**	**-1.453(3351)**	**0.146**	**-0.025**	**[-0.060, 0.009]**
Personal honour (DFR)							**0.051**	**0.044**	**1.149(3351)**	**0.251**	**0.023**	**[-0.016, 0.061]**
Partner gender [male]							0.055	0.027	2.026(16853)	0.043	0.022	[0.001, 0.044]
Partner gender [unknown]							-0.037	0.027	-1.376(16853)	0.169	-0.015	[-0.036, 0.006]
Participant gender [male]							0.264	0.069	3.842(3352)	<0.001	0.106	[0.052, 0.160]
Age							-0.002	0.002	-0.960(3353)	0.337	-0.014	[-0.041, 0.014]
Game order [SL-CG]							0.244	0.069	3.560(3355)	<0.001	0.098	[0.044, 0.152]
**Random Effects**												
σ^2	2.488						2.487					
τ_{00}	3.629 Participant_ID: Society						3.535 Participant_ID: Society					
	0.043 Society						0.043 Society					
ICC	0.596						0.590					
N	3371 Participant_ID						3371 Participant_ID					
	13 Society						13 Society					
Observations	20226						20226					
Marginal R^2 / Conditional R^2	0.005 / 0.598						0.021 / 0.599					

Note. SPR = self-promotion and retaliation, DFR = defence of family reputation, SL = step-level public goods game, CG = contest game, *std. Beta* = standardized regression coefficient, *std. 95% CI* = 95% Confidence Interval of the standardized regression coefficient. The standardization of the regression coefficients were calculated by multiplying the unstandardized coefficient by the ratio of the standard deviation of the predictor to the standard deviation of the outcome variable (this standardization approach was applied to all subsequent models). The reference level for partner gender was [female], for participant gender was [female], for game order was [CG-SL]. Societal-level honour was the societal mean of perceived normative honour values across both the SPR and DFR facets of honour, while personal honour and normative honour (centred within society) were the individual-level unweighted means across the scale items.

Table S6. Mixed-effects models with societal-level and individual-level honour values (observed scores) predicting individuals' expectations of others' competition.

	**Model S6a: Competition Expectation**						**Model S6b: Competition Expectation**					
Predictors	*B*	*SE*	*t(df)*	*p*	*std. Beta*	*std. 95% CI*	*B*	*SE*	*t(df)*	*p*	*std. Beta*	*std. 95% CI*
(Intercept)	4.773	0.852	5.600(11)	<0.001	/	/	4.527	0.853	5.308(20)	<0.001	/	/
Societal-level honour	**0.182**	**0.165**	**1.103(11)**	**0.294**	**0.037**	**[-0.037, 0.110]**	0.178	0.164	1.086(11)	0.301	0.036	[-0.037, 0.108]
Normative honour (SPR)							**0.074**	**0.035**	**2.114(3351)**	**0.035**	**0.036**	**[0.003, 0.070]**
Normative honour (DFR)							**0.138**	**0.041**	**3.392(3351)**	**0.001**	**0.065**	**[0.027, 0.102]**
Personal honour (SPR)							-0.034	0.032	-1.073(3351)	0.283	-0.018	[-0.050, 0.015]
Personal honour (DFR)							0.025	0.040	0.629(3351)	0.529	0.012	[-0.025, 0.048]
Partner gender [male]							0.218	0.028	7.678(16853)	<0.001	0.091	[0.068, 0.114]
Partner gender [unknown]							0.050	0.028	1.760(16853)	0.078	0.021	[-0.002, 0.044]
Participant gender [male]							0.121	0.063	1.934(3351)	0.053	0.051	[-0.001, 0.102]
Age							-0.004	0.002	-1.649(3352)	0.099	-0.022	[-0.048, 0.004]
Game order [SL-CG]							0.533	0.062	8.536(3353)	<0.001	0.223	[0.172, 0.274]
**Random Effects**												
σ^2	2.729						2.718					
τ_{00}	2.929 $_{\text{Participant\_ID: Society}}$						2.818 $_{\text{Participant\_ID: Society}}$					
	0.069 $_{\text{Society}}$						0.068 $_{\text{Society}}$					
ICC	0.523						0.515					
N	3371 $_{\text{Participant\_ID}}$						3371 $_{\text{Participant\_ID}}$					
	13 $_{\text{Society}}$						13 $_{\text{Society}}$					
Observations	20226						20226					
Marginal R^2 / Conditional R^2	0.001 / 0.524						0.024 / 0.526					

Note. SPR = self-promotion and retaliation, DFR = defence of family reputation, SL = step-level public goods game, CG = contest game, *std. Beta* = standardized regression coefficient, *std. 95% CI* = 95% Confidence Interval of the standardized regression coefficient. The reference level for partner gender was [female], for participant gender was [female], for game order was [CG-SL]. Societal-level honour was the societal mean of perceived normative honour values across both the SPR and DFR facets of honour, while personal honour and normative honour (centred within society) were the individual-level unweighted means across the scale items.

3.2.2 Robustness check using factor scores for honour values

In this section, we present results from models using factor scores of honour values at the societal and individual level to predict competition and expectations of other's competition as robustness checks. The results of Model S7a replicated the positive association between societal-level honour and competition found in Model S5a. At the individual-level, the results similarly replicated the positive association between each dimension of perceived normative honour values and competition (Model S7b), as well as expectations of other's competition (Model S8b), although the coefficient for perceived normative honour values of self-promotion and retaliation in predicting expectations was marginally significant. As in previous models using observed scores, factor scores of personal honour values did not predict competition or expectations of other's competition (see Model S7b and S8b).

Table S7. Mixed-effects models with societal-level and individual-level honour values (factor scores) predicting individuals' own competitive behaviours.

	**Model S7a: Competition Behaviour**						**Model S7b: Competition Behaviour**					
Predictors	*B*	*SE*	*t(df)*	*p*	*std. Beta*	*std. 95% CI*	*B*	*SE*	*t(df)*	*p*	*std. Beta*	*std. 95% CI*
(Intercept)	6.400	0.070	91.395(11)	<0.001	/	/	6.223	0.133	46.932(25)	<0.001	/	/
Societal-level honour (fs)	**0.420**	**0.187**	**2.249(11)**	**0.046**	**0.063**	**[0.001, 0.125]**	0.409	0.189	2.169(11)	0.053	0.062	[-0.001, 0.124]
Normative honour (SPR) (fs)							**0.185**	**0.081**	**2.275(3353)**	**0.023**	**0.046**	**[0.006, 0.086]**
Normative honour (DFR) (fs)							**0.152**	**0.068**	**2.248(3352)**	**0.025**	**0.052**	**[0.007, 0.097]**
Personal honour (SPR) (fs)							**-0.061**	**0.085**	**-0.718(3352)**	**0.473**	**-0.012**	**[-0.043, 0.020]**
Personal honour (DFR) (fs)							**0.063**	**0.059**	**1.068(3351)**	**0.286**	**0.020**	**[-0.017, 0.057]**
Partner gender [male]							0.055	0.027	2.026(16853)	0.043	0.022	[0.001, 0.044]
Partner gender [unknown]							-0.037	0.027	-1.376(16853)	0.169	-0.015	[-0.036, 0.006]
Participant gender [male]							0.263	0.069	3.825(3352)	<0.001	0.106	[0.052, 0.160]
Age							-0.002	0.002	-0.806(3353)	0.420	-0.011	[-0.039, 0.016]
Game order [SL-CG]							0.239	0.069	3.477(3354)	0.001	0.096	[0.042, 0.150]
**Random Effects**												
σ^2	2.488						2.487					
τ_{00}	3.629 $_{Participant\_ID:Society}$						3.546 $_{Participant\_ID:Society}$					
	0.048 $_{Society}$						0.050 $_{Society}$					
ICC	0.596						0.591					
N	3371 $_{Participant\_ID}$						3371 $_{Participant\_ID}$					
	13 $_{Society}$						13 $_{Society}$					
Observations	20226						20226					
Marginal R^2 / Conditional R^2	0.004 / 0.598						0.019 / 0.599					

Note. fs = factor score, SPR = self-promotion and retaliation, DFR = defence of family reputation, SL = step-level public goods game, CG = contest game, *std. Beta* = standardized regression coefficient, *std. 95% CI* = 95% Confidence Interval of the standardized regression coefficient. The reference level for partner gender was [female], for participant gender was [female], for game order was [CG-SL]. Societal-level honour (fs) was the societal-level factor scores of perceived normative honour values across both the SPR and DFR facets of honour, while personal honour (fs) and normative honour (fs) were the individual-level factor scores.

Table S8. Mixed-effects models with societal-level and individual-level honour values (factor scores) predicting individuals' expectations of others' competition.

	**Model S8a: Competition Expectation**						**Model S8b: Competition Expectation**					
Predictors	*B*	*SE*	*t(df)*	*p*	*std. Beta*	*std. 95% CI*	*B*	*SE*	*t(df)*	*p*	*std. Beta*	*std. 95% CI*
(Intercept)	5.709	0.071	80.566(11)	<0.001	/	/	5.426	0.124	43.691(23)	<0.001	/	/
Societal-level honour (fs)	**0.391**	**0.189**	**2.071(11)**	**0.063**	**0.061**	**[-0.004, 0.127]**	0.378	0.187	2.017(11)	0.069	0.059	[-0.005, 0.124]
Normative honour (SPR) (fs)							**0.138**	**0.074**	**1.865(3352)**	**0.062**	**0.036**	**[-0.002, 0.074]**
Normative honour (DFR) (fs)							**0.140**	**0.062**	**2.272(3352)**	**0.023**	**0.049**	**[0.007, 0.092]**
Personal honour (SPR) (fs)							-0.033	0.077	-0.431(3352)	0.666	-0.007	[-0.037, 0.023]
Personal honour (DFR) (fs)							0.027	0.053	0.503(3351)	0.615	0.009	[-0.026, 0.044]
Partner gender [male]							0.218	0.028	7.678(16853)	<0.001	0.091	[0.068, 0.114]
Partner gender [unknown]							0.050	0.028	1.760(16853)	0.078	0.021	[-0.002, 0.044]
Participant gender [male]							0.121	0.063	1.931(3351)	0.054	0.051	[-0.001, 0.102]
Age							-0.003	0.002	-1.462(3353)	0.144	-0.019	[-0.046, 0.007]
Game order [SL-CG]							0.528	0.062	8.459(3354)	<0.001	0.221	[0.170, 0.272]
**Random Effects**												
σ^2	2.729						2.718					
τ_{00}	2.929 Participant_ID:Society						2.827 Participant_ID:Society					
	0.052 Society						0.051 Society					
ICC	0.522						0.514					
N	3371 Participant_ID						3371 Participant_ID					
	13 Society						13 Society					
Observations	20226						20226					
Marginal R^2 / Conditional R^2	0.004 / 0.524						0.025 / 0.526					

Note. fs = factor score, SPR = self-promotion and retaliation, DFR = defence of family reputation, SL = step-level public goods game, CG = contest game, *std. Beta* = standardized regression coefficient, *std. 95% CI* = 95% Confidence Interval of the standardized regression coefficient. The reference level for partner gender was [female], for participant gender was [female], for game order was [CG-SL]. Societal-level honour (fs) was the societal-level factor scores of perceived normative honour values across both the SPR and DFR facets of honour, while personal honour (fs) and normative honour (fs) were the individual-level factor scores.

3.2.3 Societal-level honour as a potential moderator for personal honour values

We pre-registered to explore whether societal-level honour might moderate the relationship between individual-level personal honour values and competition. This allowed us to examine whether the broader societal and cultural context would influence the strength or direction of the relationship between personal endorsement of honour values and competition. We found no consistent evidence for a moderating effect of societal-level honour on the relationship between personal values of self-promotion and retaliation and competition, but found support for the defence of family reputation facet of personal values using both observed scores and factor scores (see Table S9). Simple slope analyses revealed that in societies with lower societal-level honour, personally endorsing values of defence of family reputation was associated with greater competition, whereas in societies with higher societal-level honour, these positive associations became weaker and nonsignificant (or even negative, see Figure S2).

Table S9. Mixed-effects models with individual-level honour values interacting with societal-level honour predicting competition.

	Competition Behaviour											
	**Model S9a: Observed scores of honour values**						**Model S9b: Factor scores of honour values**					
Predictors	*B*	*SE*	*t(df)*	*p*	*std. Beta*	*std. 95% CI*	*B*	*SE*	*t(df)*	*p*	*std. Beta*	*std. 95% CI*
(Intercept)	4.421	0.729	6.067(27)	<0.001	/	/	6.253	0.133	46.991(25)	<0.001	/	/
Personal honour (SPR)	-0.320	0.326	-0.981(3349)	0.326	-0.026	[-0.060, 0.008]	-0.058	0.085	-0.686(3350)	0.493	-0.011	[-0.042, 0.021]
Personal honour (DFR)	0.839	0.382	2.195(3349)	0.028	0.019	[-0.020, 0.058]	0.062	0.059	1.052(3349)	0.293	0.020	[-0.018, 0.057]
Societal-level honour	0.355	0.139	2.555(11)	0.027	0.069	[0.009, 0.128]	0.410	0.189	2.175(11)	0.052	0.062	[-0.001, 0.125]
Personal honour (SPR) × Soci. honour	**0.052**	**0.063**	**0.826(3349)**	**0.409**	**0.013**	**[-0.017, 0.042]**	**0.433**	**0.189**	**2.296(3349)**	**0.022**	**0.031**	**[0.005, 0.057]**
Personal honour (DFR) × Soci. honour	**-0.155**	**0.075**	**-2.077(3349)**	**0.038**	**-0.033**	**[-0.065, -0.002]**	**-0.238**	**0.115**	**-2.082(3349)**	**0.037**	**-0.029**	**[-0.056, -0.002]**
Normative honour (SPR)	0.100	0.039	2.590(3349)	0.010	0.047	[0.011, 0.082]	0.191	0.081	2.361(3351)	0.018	0.048	[0.008, 0.088]
Normative honour (DFR)	0.149	0.045	3.340(3349)	0.001	0.067	[0.028, 0.107]	0.147	0.068	2.180(3350)	0.029	0.050	[0.005, 0.095]
Partner gender [male]	0.055	0.027	2.026(16853)	0.043	0.022	[0.001, 0.044]	0.055	0.027	2.026(16853)	0.043	0.022	[0.001, 0.044]
Partner gender [unknown]	-0.037	0.027	-1.376(16853)	0.169	-0.015	[-0.036, 0.006]	-0.037	0.027	-1.376(16853)	0.169	-0.015	[-0.036, 0.006]
Participant gender [male]	0.269	0.069	3.908(3350)	<0.001	0.108	[0.054, 0.162]	0.271	0.069	3.939(3350)	<0.001	0.109	[0.055, 0.163]
Age	-0.003	0.002	-1.096(3351)	0.273	-0.016	[-0.044, 0.012]	-0.003	0.002	-1.106(3351)	0.269	-0.016	[-0.043, 0.012]
Game order [SL-CG]	0.245	0.069	3.570(3353)	<0.001	0.098	[0.044, 0.153]	0.233	0.069	3.398(3352)	0.001	0.094	[0.040, 0.148]
**Random Effects**												
σ^2	2.487						2.487					
τ_{00}	3.533 Participant_ID: Society						3.540 Participant_ID: Society					
	0.043 Society						0.050 Society					
ICC	0.590						0.591					
N	3371 Participant_ID						3371 Participant_ID					
	13 Society						13 Society					
Observations	20226						20226					
Marginal R^2 / Conditional R^2	0.022 / 0.599						0.020 / 0.599					

Note. SPR = self-promotion and retaliation, DFR = defence of family reputation, SL = step-level public goods game, CG = contest game, Soci. = Societal-level, *std. Beta* = standardized regression coefficient, *std. 95% CI* = 95% Confidence Interval of the standardized regression coefficient. The reference level for partner gender was [female], for participant gender was [female], for game order was [CG-SL]. In Model S9a, societal-level was the societal mean of perceived normative honour values across both the SPR and DFR facets of honour, while personal honour and normative honour (centred within society) were the individual-level unweighted means across the scale items. In Model S9b, societal-level honour was the societal-level factor scores of perceived normative honour values across both the SPR and DFR facets of honour, while personal honour and normative honour were the individual-level factor scores.

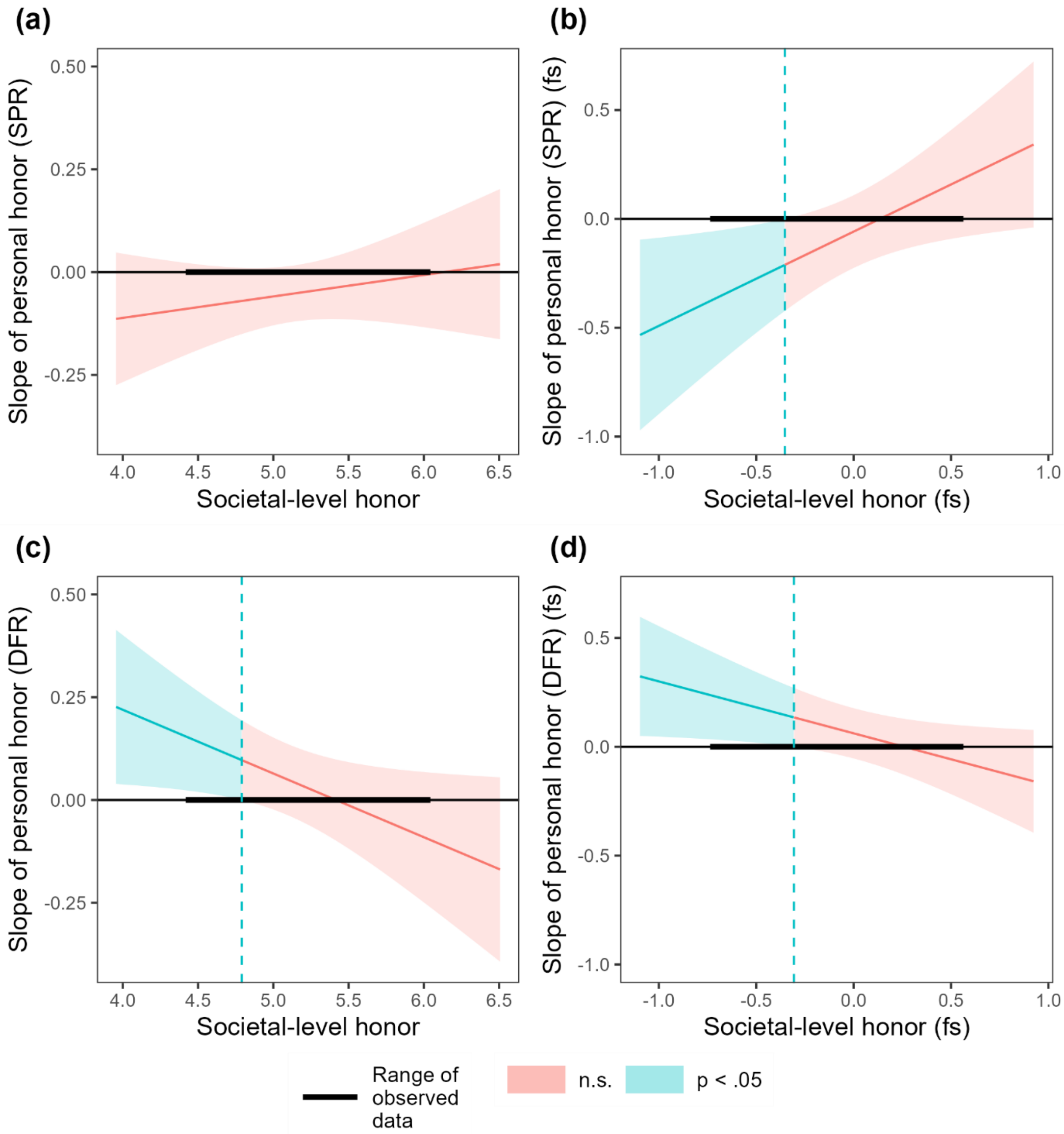

Figure S2. Johnson-Neyman plots for the interaction between personal honour values (self-promotion and retaliation, and defence of family reputation) and societal-level honour in predicting competition behaviour: (a) and (c) using observed scores for honour values, (b) and (d) using factor scores for honour values.

Note. SPR = self-promotion and retaliation, DFR = defence of family reputation, fs = factor scores. The x-axis shows the moderator societal-level honor, and y-axis shows the size of the estimated slope of each facet of personal honor values on competition behaviour. The range of observed scores of societal-level honor was [4.44, 6.03], and for factor scores, it was [-0.72, 0.55]. The solid line represents how the slope changes as the moderator (i.e., societal-level honor) increases. The shadow area represents the 95% confidence intervals around the slope estimate. The blue region marks the range of moderator values where the slope was statistically significant ($p < .05$): (a) interval could not be found, (b) outside the interval [-0.35, 1.36], (c) outside the interval [4.79, 11.26], (d) outside the interval [-0.31, 4.75].

3.2.4 Contextual effects of honour on competition and expectations about other's competition

In this section, we report the results from additional analyses that were not pre-registered to explore the potential contextual effects of honour on competition and expectations about other's competition. The contextual effects describe the differences in competition or expectations about other's competition among participants who have the same level of perceived normative and personal honour values but live in societies with different societal-level honour. In other words, even if individuals share similar perceptions of the societal prevalence of honour values or personally endorse similar levels of honour values, the broader societal context — reflected by the average prevalence of honour values perceived by all participants within the same society — may shape how individuals engage in or expect interpersonal competition. By applying a grand mean centring approach to the observed scores of perceived normative and personal honour values, we were able to further decompose the between-society effects into contextual and individual-level effects[9]. This allowed us to address whether individuals with the same level of perceived normative and personal honour vales, but inhabiting in societies with different societal mean perceived normative honour values, would engage in greater competition or expect more competition from unrelated others.

Specifically, we added grand mean-centred perceived normative and personally endorsed honour values (along with control variables such as participant age and gender, partner gender information, and game order) to Model S5a and S6a in Supplementary Section 3.2.1 to examine the fixed effect of societal-level perceived normative honour values controlling for individual level perceived normative and personal honour values — the contextual effect. The results showed that the association between societal mean perceived normative honour values and competition found in Model S5a became nonsignificant after

controlling for individual-level grand mean-centred perceived normative and personal honour values (see Model S10a). This suggested that for individuals with the same level of perceived normative and personal honour values, there was no evidence that those living in societies with higher societal mean perceived normative honour values would engage in greater competition. Comparing the *B* coefficients in Model S5b ($B = 0.356$, see Supplementary Section 3.2.1) and Model S10a ($B = 0.091$), 74% [i.e., = (0.356 – 0.091) / 0.356] of the variation explained by societal-level honour could be explained by individual-level honour values, particularly perceived normative honour values (SPR: $B = 0.096$, $p = .013$; DFR: $B = 0.155$, $p < .001$). These findings imply that the cultural contexts characterized by varying levels of honour value prevalence shape individual's engagement in interpersonal competition primarily through their perceptions of the prescribed values and norms within those cultural contexts.

Table S10. Mixed-effects models with societal-level and individual-level perceived normative honour values (grand mean centred observed scores) predicting individuals' own competitive behaviours and expectations of others' competition.

	**Model S10a: Competition Behaviour**						**Model S10b: Competition Expectation**					
Predictors	*B*	*SE*	*t(df)*	*p*	*std. Beta*	*std. 95% CI*	*B*	*SE*	*t(df)*	*p*	*std. Beta*	*std. 95% CI*
(Intercept)	5.773	0.744	7.763(28)	<0.001	/	/	5.613	0.851	6.595(21)	<0.001	/	/
Societal-level honour	**0.091**	**0.141**	**0.641(13)**	**0.533**	**0.018**	**[-0.042, 0.077]**	**-0.033**	**0.163**	**-0.202(12)**	**0.843**	**-0.007**	**[-0.078, 0.065]**
Normative honour (SPR)	0.096	0.038	2.493(3359)	0.013	0.049	[0.010, 0.087]	0.072	0.035	2.069(3362)	0.039	0.038	[0.002, 0.074]
Normative honour (DFR)	0.155	0.045	3.486(3361)	<0.001	0.077	[0.034, 0.120]	0.139	0.041	3.429(3358)	0.001	0.071	[0.031, 0.112]
Personal honour (SPR)	-0.051	0.035	-1.478(3236)	0.139	-0.028	[-0.064, 0.009]	-0.038	0.032	-1.211(3330)	0.226	-0.021	[-0.056, 0.013]
Personal honour (DFR)	0.054	0.044	1.242(3356)	0.215	0.027	[-0.016, 0.069]	0.029	0.040	0.723(3362)	0.470	0.015	[-0.025, 0.055]
Partner gender [male]	0.055	0.027	2.026(16853)	0.043	0.022	[0.001, 0.044]	0.218	0.028	7.678(16853)	<0.001	0.091	[0.068, 0.114]
Partner gender [unknown]	-0.037	0.027	-1.376(16853)	0.169	-0.015	[-0.036, 0.006]	0.050	0.028	1.760(16853)	0.078	0.021	[-0.002, 0.044]
Participant gender [male]	0.263	0.069	3.836(3352)	<0.001	0.106	[0.052, 0.160]	0.121	0.062	1.935(3352)	0.053	0.051	[-0.001, 0.102]
Age	-0.002	0.002	-0.987(3354)	0.324	-0.014	[-0.042, 0.014]	-0.004	0.002	-1.675(3353)	0.094	-0.022	[-0.048, 0.004]
Game order [SL-CG]	0.244	0.069	3.562(3355)	<0.001	0.098	[0.044, 0.152]	0.533	0.062	8.538(3353)	<0.001	0.223	[0.172, 0.274]
**Random Effects**												
σ^2	2.487						2.718					
τ_{00}	3.535 $_{\text{Participant\_ID:Society}}$						2.818 $_{\text{Participant\_ID:Society}}$					
	0.040 $_{\text{Society}}$						0.063 $_{\text{Society}}$					
ICC	0.590						0.515					
N	3371 $_{\text{Participant\_ID}}$						3371 $_{\text{Participant\_ID}}$					
	13 $_{\text{Society}}$						13 $_{\text{Society}}$					
Observations	20226						20226					
Marginal R^2 / Conditional R^2	0.022 / 0.599						0.024 / 0.526					

Note. SL = step-level public goods game, CG = contest game, *std. Beta* = standardized regression coefficient, *std. 95% CI* = 95% Confidence Interval of the standardized regression coefficient. The reference level for partner gender was [female], for participant gender was [female], for game order was [CG-SL]. Societal-level honour was the societal mean of perceived normative honour values across both the self-promotion and retaliation, and the defence of family reputation facets of honour, while normative honour (centred at the grand mean) was the individual-level unweighted means across all the scale items measuring perceived normative honour values, including both the self-promotion and retaliation, and the defence of family reputation facets of honour.

3.2.5 Additional explanation for competition: Beliefs in a zero-sum game

We pre-registered to test whether there are other cultural factors additional to honour values that may explain between-individuals level and between-societies level variation in competition. *Beliefs in a zero-sum game* is the belief that "one person's gain is possible only at the expense of other persons"[7]. With its roots in classic game theory, zero-sum belief captures the generalized beliefs about the nature of social relations involving completely conflicting interests. Previous research suggests that zero-sum belief can lead to competition and conflict, and varies across societies and social economic status[7,10]. Therefore, we examined whether beliefs in a zero-sum game could explain additional variation in competition beyond what was explained by honour values.

We did not find evidence that societal-level beliefs in a zero-sum game explained variation in competition beyond societal-level honour (see Model S11a), nor that individual-level beliefs in a zero-sum game explained variation beyond personal and perceived normative honour values (see Model S11b). These results were replicated while using factor scores of honour values and beliefs in a zero-sum game at the societal-level and individual-level (see Table S12). This indicated that beliefs in a zero-sum game may not provide additional explanation for competition beyond honour values at either level.

However, interpretations of the societal-level patterns should be approached with caution due to two key factors: Firstly, the relatively small societal-level sample size (i.e., $N_{society} = 11$) may limit the statistical power and generalizability of our findings. Secondly, results from the multilevel confirmatory factor analysis indicated that zero-sum beliefs may not vary significantly among the societies sampled in this study (see Section 2.2). At the individual level, we observed that both perceived normative and personal honour values, especially the facets of self-promotion and retaliation, were positively correlated with zero-sum game beliefs (see Figures S4a and S4b). Hence, perceived normative and personal

honour values might already account for that part of the variation in competition that could have been attributed to zero-sum beliefs.

Table S11. Mixed-effects models with societal-level and individual-level honour values and beliefs in a zero-sum game (observed scores) predicting individuals’ own competitive behaviours.

	**Model S11a: Competition Behaviour**						**Model S11b: Competition Behaviour**					
Predictors	*B*	*SE*	*t*(*df*)	*p*	*std. Beta*	*std. 95% CI*	*B*	*SE*	*t*(*df*)	*p*	*std. Beta*	*std. 95% CI*
(Intercept)	5.026	1.439	3.493(8)	0.008	/	/	4.947	1.447	3.419(17)	0.009	/	/
Societal-level honour	0.544	0.234	2.328(8)	0.049	0.090	[0.001, 0.180]	0.541	0.234	2.313(8)	0.050	0.090	[0.000, 0.179]
Societal-level BZSG	**-0.372**	**0.428**	**-0.868(8)**	**0.411**	**-0.033**	**[-0.123, 0.056]**	-0.369	0.429	-0.861(8)	0.414	-0.033	[-0.122, 0.056]
Normative honour (SPR)							0.057	0.043	1.320(2841)	0.187	0.026	[-0.012, 0.063]
Normative honour (DFR)							0.193	0.049	3.921(2841)	<0.001	0.082	[0.041, 0.123]
Personal honour (SPR)							-0.021	0.040	-0.535(2841)	0.593	-0.010	[-0.048, 0.028]
Personal honour (DFR)							0.032	0.048	0.671(2841)	0.502	0.014	[-0.027, 0.054]
Beliefs in a zero-sum game							**-0.003**	**0.040**	**-0.068(2841)**	**0.946**	**-0.001**	**[-0.033, 0.031]**
Partner gender [male]							0.064	0.030	2.174(14298)	0.030	0.026	[0.003, 0.049]
Partner gender [unknown]							-0.031	0.030	-1.037(14298)	0.300	-0.012	[-0.036, 0.011]
Participant gender [male]							0.244	0.075	3.274(2841)	0.001	0.098	[0.039, 0.157]
Age							-0.005	0.003	-1.753(2843)	0.080	-0.027	[-0.057, 0.003]
Game order [SL-CG]							0.286	0.074	3.844(2843)	<0.001	0.115	[0.056, 0.174]
**Random Effects**												
σ^2	2.492						2.491					
τ_{00}	3.630 $_{\text{Participant\_ID:Society}}$						3.539 $_{\text{Participant\_ID:Society}}$					
	0.055 $_{\text{Society}}$						0.055 $_{\text{Society}}$					
ICC	0.596						0.591					
N	2860 $_{\text{Participant\_ID}}$						2860 $_{\text{Participant\_ID}}$					
	11 $_{\text{Society}}$						11 $_{\text{Society}}$					
Observations	17160						17160					
Marginal R^2 / Conditional R^2	0.006 / 0.599						0.022 / 0.600					

Note. BZSG = beliefs in a zero-sum game, SPR = self-promotion and retaliation, DFR = defence of family reputation, SL = step-level public goods game, CG = contest game, *std. Beta* = standardized regression coefficient, *std. 95% CI* = 95% Confidence Interval of the standardized regression coefficient. The reference level for partner gender was [female], for participant gender was [female], for game order was [CG-SL]. Societal-level honour was the societal mean of perceived normative honour values across both the SPR and DFR facets of honour, and societal-level BZSG was the societal mean of beliefs in a zero-sum game, while personal honour, normative honour and beliefs in a zero-sum game (centred within society) were the individual-level unweighted means across the scale items. We did not include data on beliefs in a zero-sum game for the UK and the USA because participants’ responses in these two societies were recorded on a seven-point scale due to a survey programming error, while a six-point scale was used in the other samples.

Table S12. Mixed-effects models with societal-level and individual-level honour values and beliefs in a zero-sum game (factor scores) predicting individuals' own competitive behaviours.

	**Model S12a: Competition Behaviour**						**Model S12b: Competition Behaviour**					
Predictors	*B*	*SE*	*t*(*df*)	*p*	*std. Beta*	*std. 95% CI*	*B*	*SE*	*t*(*df*)	*p*	*std. Beta*	*std. 95% CI*
(Intercept)	6.374	0.098	64.784(8)	<0.001	/	/	6.262	0.158	39.709(16)	<0.001	/	/
Societal-level honour (fs)	0.459	0.442	1.039(8)	0.330	0.049	[-0.061, 0.160]	0.444	0.445	0.998(8)	0.348	0.048	[-0.063, 0.158]
Societal-level BZSG (fs)	**-0.358**	**0.598**	**-0.599(8)**	**0.566**	**-0.029**	**[-0.139, 0.082]**	-0.374	0.601	-0.622(8)	0.551	-0.030	[-0.141, 0.081]
Normative honour (SPR) (fs)							0.106	0.090	1.172(2842)	0.241	0.026	[-0.017, 0.069]
Normative honour (DFR) (fs)							0.222	0.074	2.981(2842)	0.003	0.073	[0.025, 0.121]
Personal honour (SPR) (fs)							0.027	0.096	0.285(2842)	0.776	0.005	[-0.030, 0.040]
Personal honour (DFR) (fs)							0.039	0.064	0.606(2841)	0.545	0.012	[-0.028, 0.052]
Beliefs in a zero-sum game (fs)							**-0.001**	**0.058**	**-0.020(2841)**	**0.984**	**-0.000**	**[-0.030, 0.030]**
Partner gender [male]							0.064	0.030	2.174(14298)	0.030	0.026	[0.003, 0.049]
Partner gender [unknown]							-0.031	0.030	-1.037(14298)	0.300	-0.012	[-0.036, 0.011]
Participant gender [male]							0.244	0.075	3.273(2841)	0.001	0.098	[0.039, 0.157]
Age							-0.004	0.003	-1.507(2843)	0.132	-0.023	[-0.053, 0.007]
Game order [SL-CG]							0.285	0.074	3.834(2842)	<0.001	0.115	[0.056, 0.173]
**Random Effects**												
σ^2	2.492						2.491					
τ_{00}	3.630 Participant_ID:Society						3.540 Participant_ID:Society					
	0.060 Society						0.061 Society					
ICC	0.597						0.591					
N	2860 Participant_ID						2860 Participant_ID					
	11 Society						11 Society					
Observations	17160						17160					
Marginal R^2 / Conditional R^2	0.005 / 0.599						0.022 / 0.600					

Note. fs = factor score, BZSG = beliefs in a zero-sum game, SPR = self-promotion and retaliation, DFR = defence of family reputation, SL = step-level public goods game, CG = contest game, *std. Beta* = standardized regression coefficient, *std. 95% CI* = 95% Confidence Interval of the standardized regression coefficient. The reference level for partner gender was [female], for participant gender was [female], for game order was [CG-SL]. Societal-level honour (fs) was the societal-level factor scores of perceived normative honour values across both the SPR and DFR facets of honour, and societal-level BZSG (fs) was the societal-level factor scores of beliefs in a zero-sum game, while personal honour (fs), normative honour (fs) and beliefs in a zero-sum game (fs) were the individual-level factor scores. We did not include data on beliefs in a zero-sum game for the UK and the USA because participants' responses in these two societies were recorded on a seven-point scale due to a survey programming error, while a six-point scale was used in the other samples.

3.2.6 Other societal-level indicators and competition

In this section, we report the results of a series of mixed effects models, where each model included one societal-level indicator predicting competition (see Table S13 for the operationalization of these societal-level indicators). The results showed that among all these societal-level indicators, only GNI ($\beta = –.060$, $p = .047$), and market competitiveness ($\beta = –.063$, $p = .037$) were negatively associated with competition (see Table S14). Interestingly, intercorrelations revealed that GNI and market competitiveness were among the strongest societal-level indicators that negatively correlated with societal-level honour (see Figure S4a and S4b). Here, GNI captures the economic wealth of a country. The Global Competitiveness Index (GCI), used to operationalize market competitiveness, is a complex indicator that assesses the ability of countries to provide high levels of prosperity to their citizens. Thus, within the sample of societies included in our dataset, those societies where honour values were perceived to be more prevalent tended to be harsher environments characterized by lower economic development and prosperity, where more interpersonal competition has been observed (see Section 3.3.6 for analyses on the association between other societal-level indicators and cooperation).

Table S13. Operationalization of societal-level indicators.

Societal-level indicator	Description	Range	Source	Year
Economic indicators				
GDP per capita	Gross Domestic Product per capita (current US$)	N/A	World Bank	2022
GNI	Gross National Income per capita (Atlas method, current US$)	N/A	World Bank	2022
Human Development Index	A summary measure of average achievement in key dimensions of human development: a long and healthy life, being knowledgeable and having a decent standard of living.	0-1	Human Development Report	2021
Gender Inequality Index	A composite measure reflecting inequality in achievement between women and men in three dimensions: reproductive health, empowerment and the labour market.	0-1	Human Development Report	2021
Institutions				
Government Effectiveness	Government effectiveness	−2.5–2.5	World Bank	2022
Rule of law	Rule of Law	−2.5–2.5	World Bank	2022
Political Stability and Absence of Violence/Terrorism	Political Stability and Absence of Violence/Terrorism	−2.5–2.5	World Bank	2022
Corruption control	Control of corruption	−2.5–2.5	World Bank	2022
Corruption perception index	Perceived levels of public sector corruption	0-100	Transparency International	2022
Market competitiveness	Global competitiveness index (GCI), which captures the microeconomic and macroeconomic foundations of national competitiveness.	0-100	World Economic Forum	2019
Historical and ecological threats				
Historical prevalence of pathogens	Prevalence of leishmanias, schistosomes, trypanosomes, leprosy, malaria, typhus, filariae, dengue, and tuberculosis	0–3	Murray & Schaller (2010)[11]	N/A
World risk index	The disaster risk from extreme natural events and negative climate change impacts (Interaction of exposure and vulnerability).	0-100	The World Risk Report	2022
Exposure	The extent to which populations in hazard-prone areas are exposed to and burdened by the impacts of extreme natural events or the negative consequences of climate change.	0-100	The World Risk Report	2022
Vulnerability	The predisposition of populations to be vulnerable to damage from extreme natural events or negative impacts of climate change. It is composed of the three dimensions of susceptibility, lack of coping capacities, and lack of adaptive capacities, which are subdivided into further categories.	0-100	The World Risk Report	2022

Table S14. A series of mixed-effects models for each societal-level indicator predicting competition in separate models.

		**Models S13a-S13n: Competition Behaviour**								
#	**Societal-level indicator**	***B***	***SE***	***t*(*df*)**	***p***	***std. Beta***	***std. 95%CI***	$N_{society}$	$N_{participant}$	$N_{observation}$
	Economic indicators									
a	GDP per capita	-0.149	0.071	-2.095(10)	0.063	-0.057	[-0.111, -0.004]	12	3126	18756
b	GNI	-0.157*	0.069	-2.260(10)	0.047	-0.060	[-0.112, -0.008]	12	3126	18756
c	Human development index	-0.132	0.074	-1.773(10)	0.107	-0.051	[-0.107, 0.005]	12	3126	18756
d	Gender inequality	0.143	0.072	1.989(10)	0.075	0.055	[0.001, 0.110]	12	3126	18756
	Institutions									
e	Government effectiveness	-0.093	0.080	-1.156(10)	0.274	-0.036	[-0.096, 0.025]	12	3126	18756
f	Rule of law	-0.016	0.085	-0.192(10)	0.852	-0.006	[-0.070, 0.058]	12	3126	18756
g	Political stability	-0.018	0.085	-0.206(10)	0.841	-0.007	[-0.071, 0.057]	12	3126	18756
h	Corruption control	-0.051	0.084	-0.613(10)	0.554	-0.020	[-0.083, 0.043]	12	3126	18756
i	Corruption perceptions index	-0.136	0.074	-1.844(10)	0.095	-0.052	[-0.108, 0.003]	12	3126	18756
j	Market competitiveness	-0.163*	0.068	-2.407(10)	0.037	-0.063	[-0.114, -0.012]	12	3126	18756
	Historical and ecological threats									
k	Historical prevalence of infectious disease	0.122	0.076	1.596(10)	0.141	0.047	[-0.011, 0.104]	12	3126	18756
l	World risk index	0.092	0.080	1.146(10)	0.278	0.035	[-0.025, 0.096]	12	3126	18756
m	Exposure	-0.027	0.085	-0.320(10)	0.756	-0.010	[-0.074, 0.054]	12	3126	18756
n	Vulnerability	0.112	0.077	1.451(10)	0.178	0.043	[-0.015, 0.102]	12	3126	18756

Note. std. Beta = standardized regression coefficient, *std. 95% CI* = 95% Confidence Interval of the standardized regression coefficient, $N_{society}$ = number of societies, $N_{participant}$ = number of participants, $N_{observation}$ = number of observations at the decision level. Unstandardized regression coefficients (*B*) were marked with an asterisk if they were significant at the 0.05 level. See Table S13 for the operationalization for each societal-level indicator.

3.3 Honour and cooperation

In this section, we present the results addressing our main research questions on honour and cooperation (Section 3.3.1) and their robustness checks (Section 3.3.2), as well as exploratory analyses related to honour and cooperation (Section 3.3.3 to 3.3.8). First, we ran models with societal-level and individual-level honour values (using *observed scores*) predicting cooperation and expectations of other's cooperation in separate models (Table S15-S16). Next, we conducted robustness checks by using *factor scores* of honour values to predict cooperation and expectations of other's cooperation (Table S17-S18). The rationale for conducting these robustness checks can be found in the unregistered steps outlined in Supplementary Section 1, while details of the factor analysis were provided in Supplementary Section 2.

We also explored potential cross-level interactions, specifically whether societal-level honour would interact with individual-level honour values in predicting cooperation. This exploratory analysis used both observed scores and factor scores (Table S19). We then explored whether there was contextual effect of societal-level honour on cooperation and expectations of other's cooperation, while controlling for individual-level grand mean centred perceived normative honour values (Table S20). Additional, we tested whether relational mobility would account for additional variance in cooperation beyond honour values at both the societal and individual levels (Table S21-S22). Moreover, we explored a set of societal-level indicators that are theoretically relevant to the emergence of the cultural logic of honour and examined their associations with cooperation (Table S23).

As pre-registered, we further conducted secondary analyses of existing datasets to explore whether societal-level honour relates to cooperation versus non-cooperation in situations where non-cooperation can always yield the best outcome for an individual regardless of what others do (i.e., prisoner's dilemma, public goods dilemma, Table S24-

S26). Finally, we conducted analyses exploring the association between societal-level honour or individual-level honour values and the likelihood of contributing 8 MUs or contributing 6 MUs following a compelling decision rule of equally splitting the cost of meeting the provision points in the step-level public goods game (Table S27-S28).

3.3.1 Honour, cooperation and expectations about other's cooperation

In this section, we present the results from models with societal-level honour predicting cooperation (Model S15a) or expectations of other's cooperation (Model S16a). Results showed that societal-level honour was positively associated with cooperation and with expectations of other's competition. We also fitted an intercept-only model with society and participant as random intercepts (see Model "m_SLB_0" in the online syntax "data_analysis_final.Rmd" on OSF), which showed that the variance of cooperation at the society level was 0.067. When societal-level honour was added as a predictor, this variance decreased to 0.035 (see Model S15a), suggesting that societal-level honour accounted for 47.76% of the societal-level variance in competition.

We then added four individual-level honour value indicators to the model to test whether perceived normative and personal honour values were associated with cooperation (Model S15b) or expectations of other's cooperation (Model S15b). The models controlled for partner gender information, participant gender, age, and order of the game. The generalized variance inflation factor adjusted for the degree of freedom indicated a low risk of multicollinearity in both models [all the $\mathrm{GVIF}^{1/(2\times \mathrm{Df})} < 2$] (see Models "m_SLB_2_vif" and "m_SLE_2_vif" in the online syntax "data_analysis_final.Rmd" on OSF). The model results showed that perceiving honour values of self-promotion and retaliation as more prevalent in one's society, positively predicted cooperation (see Model 15b) and expectations of other's cooperation (though marginally significant, see Model 16b). Perceiving honour values of defence of family reputation to be more prevalent in one's society positively

predicted expectations of other's cooperation (see Model 16b), and while its association with individuals' own cooperation was not significant, it exhibited a positive trend (see Model 15b). Personally endorsing honour values related to defence of family reputation was also positively associated with individual's own cooperation (see Model 15b) and expectations of other's cooperation (see Model 15b). However, personal values of self-promotion and retaliation were negatively associated with expectations of other's cooperation (see Model S16b), and although their association with individuals' own cooperation was not significant, the direction of the relationship was also negative (see Model 15b).

For testing individual-level honour indicators, we conducted robustness checks by adding three additional demographic variables as controls into Models S15b and S16b: parents' education levels (1-8), belongingness to the ethnic majority group in the respective society (no, yes), and living environment (rural, urban, both). There analyses were conducted in Models named "m_SLB_2_RC" and "m_SLE_2_RC" in the online syntax "data_analysis_final.Rmd" on OSF. The results largely replicated findings from Models S15b and S16b. Specifically, perceived normative values of self-promotion and retaliation predicted more cooperation ($\beta = .047$, $p = .007$, see results from Model object "m_SLB_2_RC"), and perceived normative values of both facets of honour predicted greater expectation of other's cooperation ($\beta = .036$, $p = .037$ (SPR); $\beta = .068$, $p < .001$ (DFR), see results from Model object "m_SLE_2_RC"). The two facets of personal honour values showed contrasting associations. Personal values of defence of family reputation positively predicted both cooperation ($\beta = .060$, $p = .002$) and expectations of others' cooperation ($\beta = .042$, $p = .023$), while personal values of self-promotion and retaliation negatively predicted expectations of others' cooperation ($\beta = –.043$, $p = .009$).

Table S15. Mixed-effects models with societal-level and individual-level honour values (observed scores) predicting individuals' own cooperative behaviours.

	**Model S15a: Cooperation Behaviour**						**Model S15b: Cooperation Behaviour**					
Predictors	*B*	*SE*	*t(df)*	*p*	*std. Beta*	*std. 95% CI*	*B*	*SE*	*t(df)*	*p*	*std. Beta*	*std. 95% CI*
(Intercept)	4.286	0.652	6.573(11)	<0.001	/	/	4.309	0.667	6.460(27)	<0.001	/	/
Societal-level honour	**0.376**	**0.126**	**2.974(11)**	**0.013**	**0.078**	**[0.020, 0.136]**	0.381	0.127	2.993(11)	0.012	0.080	[0.021, 0.138]
Normative honour (SPR)							**0.095**	**0.034**	**2.784(3351)**	**0.005**	**0.048**	**[0.014, 0.082]**
Normative honour (DFR)							**0.064**	**0.040**	**1.622(3351)**	**0.105**	**0.031**	**[-0.007, 0.069]**
Personal honour (SPR)							**-0.029**	**0.031**	**-0.951(3351)**	**0.342**	**-0.016**	**[-0.049, 0.017]**
Personal honour (DFR)							**0.118**	**0.039**	**3.000(3351)**	**0.003**	**0.057**	**[0.020, 0.093]**
Partner gender [male]							-0.019	0.027	-0.725(16853)	0.468	-0.008	[-0.031, 0.014]
Partner gender [unknown]							-0.012	0.027	-0.437(16853)	0.662	-0.005	[-0.028, 0.018]
Participant gender [male]							0.113	0.061	1.846(3352)	0.065	0.049	[-0.003, 0.101]
Age							0.003	0.002	1.570(3353)	0.116	0.021	[-0.005, 0.048]
Game order [SL-CG]							-0.471	0.061	-7.720(3354)	<0.001	-0.204	[-0.256, -0.152]
**Random Effects**												
σ^2	2.420						2.420					
τ_{00}	2.846 $_{\text{Participant\_ID:Society}}$						2.727 $_{\text{Participant\_ID:Society}}$					
	0.035 $_{\text{Society}}$						0.037 $_{\text{Society}}$					
ICC	0.544						0.533					
N	3371 $_{\text{Participant\_ID}}$						3371 $_{\text{Participant\_ID}}$					
	13 $_{\text{Society}}$						13 $_{\text{Society}}$					
Observations	20226						20226					
Marginal R^2 / Conditional R^2	0.006 / 0.546						0.029 / 0.547					

Note. SPR = self-promotion and retaliation, DFR = defence of family reputation, SL = step-level public goods game, CG = contest game, *std. Beta* = standardized regression coefficient, *std. 95% CI* = 95% Confidence Interval of the standardized regression coefficient. The reference level for partner gender was [female], for participant gender was [female], for game order was [CG-SL]. Societal-level honour was the societal mean of perceived normative honour values across both the SPR and DFR facets of honour, while personal honour and normative honour (centred within society) were the individual-level unweighted means across the scale items.

Table S16. Mixed-effects models with societal-level and individual-level honour values (observed scores) predicting individuals' expectations of others' cooperation.

	**Model S16a: Cooperation Expectation**						**Model S16b: Cooperation Expectation**					
Predictors	*B*	*SE*	*t(df)*	*p*	*std. Beta*	*std. 95% CI*	*B*	*SE*	*t(df)*	*p*	*std. Beta*	*std. 95% CI*
(Intercept)	4.391	0.635	6.910(11)	<0.001	/	/	4.342	0.647	6.709(26)	<0.001	/	/
Societal-level honour	**0.306**	**0.123**	**2.488(11)**	**0.030**	**0.066**	**[0.008, 0.125]**	0.309	0.124	2.496(11)	0.030	0.067	[0.008, 0.126]
Normative honour (SPR)							**0.061**	**0.032**	**1.914(3351)**	**0.056**	**0.032**	**[-0.001, 0.065]**
Normative honour (DFR)							**0.139**	**0.037**	**3.763(3351)**	**<0.001**	**0.070**	**[0.034, 0.107]**
Personal honour (SPR)							-0.080	0.029	-2.768(3351)	0.006	-0.045	[-0.076, -0.013]
Personal honour (DFR)							0.090	0.036	2.485(3351)	0.013	0.045	[0.010, 0.081]
Partner gender [male]							0.174	0.027	6.342(16853)	<0.001	0.078	[0.054, 0.102]
Partner gender [unknown]							0.047	0.027	1.713(16853)	0.087	0.021	[-0.003, 0.045]
Participant gender [male]							0.056	0.057	0.990(3352)	0.322	0.025	[-0.025, 0.075]
Age							0.001	0.002	0.734(3353)	0.463	0.010	[-0.016, 0.035]
Game order [SL-CG]							-0.253	0.057	-4.456(3354)	<0.001	-0.114	[-0.164, -0.064]
**Random Effects**												
σ^2	2.530						2.524					
τ_{00}	2.361 $_{\text{Participant\_ID:Society}}$						2.285 $_{\text{Participant\_ID:Society}}$					
	0.035 $_{\text{Society}}$						0.035 $_{\text{Society}}$					
ICC	0.486						0.479					
N	3371 $_{\text{Participant\_ID}}$						3371 $_{\text{Participant\_ID}}$					
	13 $_{\text{Society}}$						13 $_{\text{Society}}$					
Observations	20226						20226					
Marginal R^2 / Conditional R^2	0.004 / 0.489						0.022 / 0.490					

Note. SPR = self-promotion and retaliation, DFR = defence of family reputation, SL = step-level public goods game, CG = contest game, *std. Beta* = standardized regression coefficient, *std. 95% CI* = 95% Confidence Interval of the standardized regression coefficient. The reference level for partner gender was [female], for participant gender was [female], for game order was [CG-SL]. Societal-level honour was the societal mean of perceived normative honour values across both the SPR and DFR facets of honour, while personal honour and normative honour (centred within society) were the individual-level unweighted means across the scale items.

3.3.2 Robustness check using factor scores for honour values

In this section, we present results from models using factor scores of honour values at the societal and individual level to predict cooperation and expectations of other's cooperation as robustness checks. The results of Model S17a replicated the positive association between societal-level honour and cooperation found in Model S15a. Additionally, using factor scores, societal-level honour also positively predicted expectations of other's cooperation (see Model S18a). At the individual-level, the results replicated the positive association between perceived normative honour values of self-promotion and retaliation (and persona values of defence of family reputation) and cooperation (see Model S17b), as well as the positive association between perceived normative values of defence of family reputation and expectations of other's cooperation (see Model S18b).

Table S17. Mixed-effects models with societal-level and individual-level honour values (factor scores) predicting individuals' own cooperative behaviours.

	**Model S17a: Cooperation Behaviour**						**Model S17b: Cooperation Behaviour**					
Predictors	*B*	*SE*	*t(df)*	*p*	*std. Beta*	*std. 95% CI*	*B*	*SE*	*t(df)*	*p*	*std. Beta*	*std. 95% CI*
(Intercept)	6.217	0.068	92.092(11)	<0.001	/	/	6.235	0.121	51.333(23)	<0.001	/	/
Societal-level honour (fs)	**0.405**	**0.180**	**2.247(11)**	**0.046**	**0.066**	**[0.001, 0.130]**	0.408	0.182	2.238(11)	0.047	0.066	[0.001, 0.131]
Normative honour (SPR) (fs)							**0.148**	**0.072**	**2.049(3353)**	**0.041**	**0.040**	**[0.002, 0.078]**
Normative honour (DFR) (fs)							**0.016**	**0.060**	**0.269(3352)**	**0.788**	**0.006**	**[-0.037, 0.049]**
Personal honour (SPR) (fs)							**-0.072**	**0.076**	**-0.949(3352)**	**0.343**	**-0.015**	**[-0.045, 0.016]**
Personal honour (DFR) (fs)							**0.122**	**0.053**	**2.322(3351)**	**0.020**	**0.042**	**[0.007, 0.078]**
Partner gender [male]							-0.019	0.027	-0.725(16853)	0.468	-0.008	[-0.031, 0.014]
Partner gender [unknown]							-0.012	0.027	-0.437(16853)	0.662	-0.005	[-0.028, 0.018]
Participant gender [male]							0.119	0.061	1.943(3352)	0.052	0.052	[-0.000, 0.104]
Age							0.004	0.002	1.961(3353)	0.050	0.027	[0.000, 0.053]
Game order [SL-CG]							-0.478	0.061	-7.798(3354)	<0.001	-0.207	[-0.259, -0.155]
**Random Effects**												
σ^2	2.420						2.420					
τ_{00}	2.846 Participant_ID:Society						2.759 Participant_ID:Society					
	0.047 Society						0.048 Society					
ICC	0.545						0.537					
N	3371 Participant_ID						3371 Participant_ID					
	13 Society						13 Society					
Observations	20226						20226					
Marginal R^2 / Conditional R^2	0.004 / 0.546						0.022 / 0.547					

Note. fs = factor score, SPR = self-promotion and retaliation, DFR = defence of family reputation, SL = step-level public goods game, CG = contest game, *std. Beta* = standardized regression coefficient, *std. 95% CI* = 95% Confidence Interval of the standardized regression coefficient. The reference level for partner gender was [female], for participant gender was [female], for game order was [CG-SL]. Societal-level honour (fs) was the societal-level factor scores of perceived normative honour values across both the SPR and DFR facets of honour, while personal honour (fs) and normative honour (fs) were the individual-level factor scores.

Table S18. Mixed-effects models with societal-level and individual-level honour values (factor scores) predicting individuals' expectations of others' cooperation.

	**Model S18a: Cooperation Expectation**						**Model S18b: Cooperation Expectation**					
Predictors	*B*	*SE*	*t(df)*	*p*	*std. Beta*	*std. 95% CI*	*B*	*SE*	*t(df)*	*p*	*std. Beta*	*std. 95% CI*
(Intercept)	5.965	0.053	112.808(11)	<0.001	/	/	5.923	0.107	55.288(31)	<0.001	/	/
Societal-level honour (fs)	**0.457**	**0.141**	**3.240(11)**	**0.008**	**0.077**	**[0.025, 0.129]**	0.453	0.141	3.217(11)	0.008	0.076	[0.024, 0.129]
Normative honour (SPR) (fs)							**0.115**	**0.067**	**1.715(3353)**	**0.086**	**0.032**	**[-0.005, 0.069]**
Normative honour (DFR) (fs)							**0.149**	**0.056**	**2.660(3353)**	**0.008**	**0.057**	**[0.015, 0.098]**
Personal honour (SPR) (fs)							-0.120	0.070	-1.700(3352)	0.089	-0.025	[-0.055, 0.004]
Personal honour (DFR) (fs)							0.126	0.049	2.584(3352)	0.010	0.045	[0.011, 0.080]
Partner gender [male]							0.174	0.027	6.342(16853)	<0.001	0.078	[0.054, 0.102]
Partner gender [unknown]							0.047	0.027	1.713(16853)	0.087	0.021	[-0.003, 0.045]
Participant gender [male]							0.055	0.057	0.963(3352)	0.335	0.025	[-0.026, 0.075]
Age							0.002	0.002	0.855(3354)	0.393	0.011	[-0.014, 0.037]
Game order [SL-CG]							-0.257	0.057	-4.526(3355)	<0.001	-0.116	[-0.166, -0.066]
**Random Effects**												
σ^2	2.530						2.524					
τ_{00}	2.361 Participant_ID:Society						2.289 Participant_ID:Society					
	0.026 Society						0.026 Society					
ICC	0.485						0.478					
N	3371 Participant_ID						3371 Participant_ID					
	13 Society						13 Society					
Observations	20226						20226					
Marginal R^2 / Conditional R^2	0.006 / 0.488						0.023 / 0.490					

Note. fs = factor score, SPR = self-promotion and retaliation, DFR = defence of family reputation, SL = step-level public goods game, CG = contest game, *std. Beta* = standardized regression coefficient, *std. 95% CI* = 95% Confidence Interval of the standardized regression coefficient. The reference level for partner gender was [female], for participant gender was [female], for game order was [CG-SL]. Societal-level honour (fs) was the societal-level factor scores of perceived normative honour values across both the SPR and DFR facets of honour, while personal honour (fs) and normative honour (fs) were the individual-level factor scores.

3.3.3 Societal-level honour as a potential moderator for personal honour values

We pre-registered to explore whether societal-level honour might moderate the relationship between personal honour values and cooperation. We found consistent support for the moderating role of societal-level honour in the association between personal values of both dimensions and cooperation, using observed scores and factor scores (see Table S19). Simple slope analyses revealed that in societies with lower societal-level honour, personally endorsing values of self-promotion and retaliation was associated with less cooperation, whereas in societies with higher societal-level honour, these negative associations became weaker, nonsignificant, and even positive (see Figure S3). However, the moderating effect was reversed for the defence of family reputation: in societies with lower societal-level honour, personally endorsing values of defence of family reputation was associated with more cooperation, whereas in societies with higher societal-level honour, these positive associations became weaker and nonsignificant (see Figure S3). These results indicated that personally endorsing the two facets of honour values had a more similar relationship with cooperation in societies with higher societal-level honour. However, in societies with lower societal-level honour, different mechanisms emerged for the two facets of personal honour values. In these contexts, personally endorsing self-promotion and retaliation may hinder cooperation, while personally endorsing defence of family reputation may play a more positive role in fostering cooperation.

Table S19. Mixed-effects models with individual-level honour values interacting with societal-level honour predicting cooperation.

	Cooperation Behaviour											
	Model S19a: Observed scores of honour values						Model S19b: Factor scores of honour values					
Predictors	*B*	*SE*	*t(df)*	*p*	*std. Beta*	*std. 95% CI*	*B*	*SE*	*t(df)*	*p*	*std. Beta*	*std. 95% CI*
(Intercept)	4.347	0.668	6.506(27)	<0.001	/	/	6.272	0.122	51.553(23)	<0.001	/	/
Personal honour (SPR)	-0.800	0.290	-2.762(3349)	0.006	-0.016	[-0.049, 0.017]	-0.069	0.076	-0.904(3350)	0.366	-0.014	[-0.044, 0.017]
Personal honour (DFR)	0.980	0.340	2.883(11)	0.004	0.054	[0.017, 0.092]	0.121	0.052	2.313(3350)	0.021	0.042	[0.006, 0.078]
Societal-level honour	0.381	0.128	2.983(3349)	0.013	0.079	[0.021, 0.138]	0.409	0.182	2.252(11)	0.046	0.066	[0.001, 0.131]
Personal honour (SPR) × Soci. honour	**0.150**	**0.056**	**2.672(3349)**	**0.008**	**0.039**	**[0.010, 0.068]**	**0.491**	**0.168**	**2.916(3349)**	**0.004**	**0.038**	**[0.012, 0.063]**
Personal honour (DFR) × Soci. honour	**-0.169**	**0.066**	**-2.541(3349)**	**0.011**	**-0.039**	**[-0.069, -0.009]**	**-0.307**	**0.102**	**-2.999(3349)**	**0.003**	**-0.040**	**[-0.066, -0.014]**
Normative honour (SPR)	0.096	0.034	2.797(3349)	0.005	0.049	[0.015, 0.083]	0.156	0.072	2.161(3351)	0.031	0.042	[0.004, 0.081]
Normative honour (DFR)	0.062	0.040	1.554(3349)	0.120	0.030	[-0.008, 0.068]	0.010	0.060	0.167(3350)	0.867	0.004	[-0.040, 0.047]
Partner gender [male]	-0.019	0.027	-0.725(16853)	0.468	-0.008	[-0.031, 0.014]	-0.019	0.027	-0.725(16853)	0.468	-0.008	[-0.031, 0.014]
Partner gender [unknown]	-0.012	0.027	-0.437(16853)	0.662	-0.005	[-0.028, 0.018]	-0.012	0.027	-0.437(16853)	0.662	-0.005	[-0.028, 0.018]
Participant gender [male]	0.122	0.061	2.001(3350)	0.045	0.053	[0.001, 0.105]	0.129	0.061	2.097(3350)	0.036	0.056	[0.004, 0.108]
Age	0.003	0.002	1.140(3351)	0.254	0.016	[-0.011, 0.042]	0.003	0.002	1.529(3351)	0.126	0.021	[-0.006, 0.048]
Game order [SL-CG]	-0.475	0.061	-7.782(3352)	<0.001	-0.206	[-0.258, -0.154]	-0.484	0.061	-7.904(3352)	<0.001	-0.210	[-0.262, -0.158]
**Random Effects**												
σ^2	2.420						2.420					
τ_{00}	2.720 $_{\text{Participant\_ID:Society}}$						2.748 $_{\text{Participant\_ID:Society}}$					
	0.037 $_{\text{Society}}$						0.048 $_{\text{Society}}$					
ICC	0.533						0.536					
N	3371 $_{\text{Participant\_ID}}$						3371 $_{\text{Participant\_ID}}$					
	13 $_{\text{Society}}$						13 $_{\text{Society}}$					
Observations	20226						20226					
Marginal R^2 / Conditional R^2	0.031 / 0.547						0.024 / 0.547					

Note. SPR = self-promotion and retaliation, DFR = defence of family reputation, SL = step-level public goods game, CG = contest game, Soci. = Societal-level, *std. Beta* = standardized regression coefficient, *std. 95% CI* = 95% Confidence Interval of the standardized regression coefficient. The reference level for partner gender was [female], for participant gender was [female], for game order was [CG-SL]. Societal-level honour was the societal mean of perceived normative honour values across both the SPR and DFR facets of honour, while personal honour and normative honour (centred within society) were the individual-level unweighted means across the scale items.

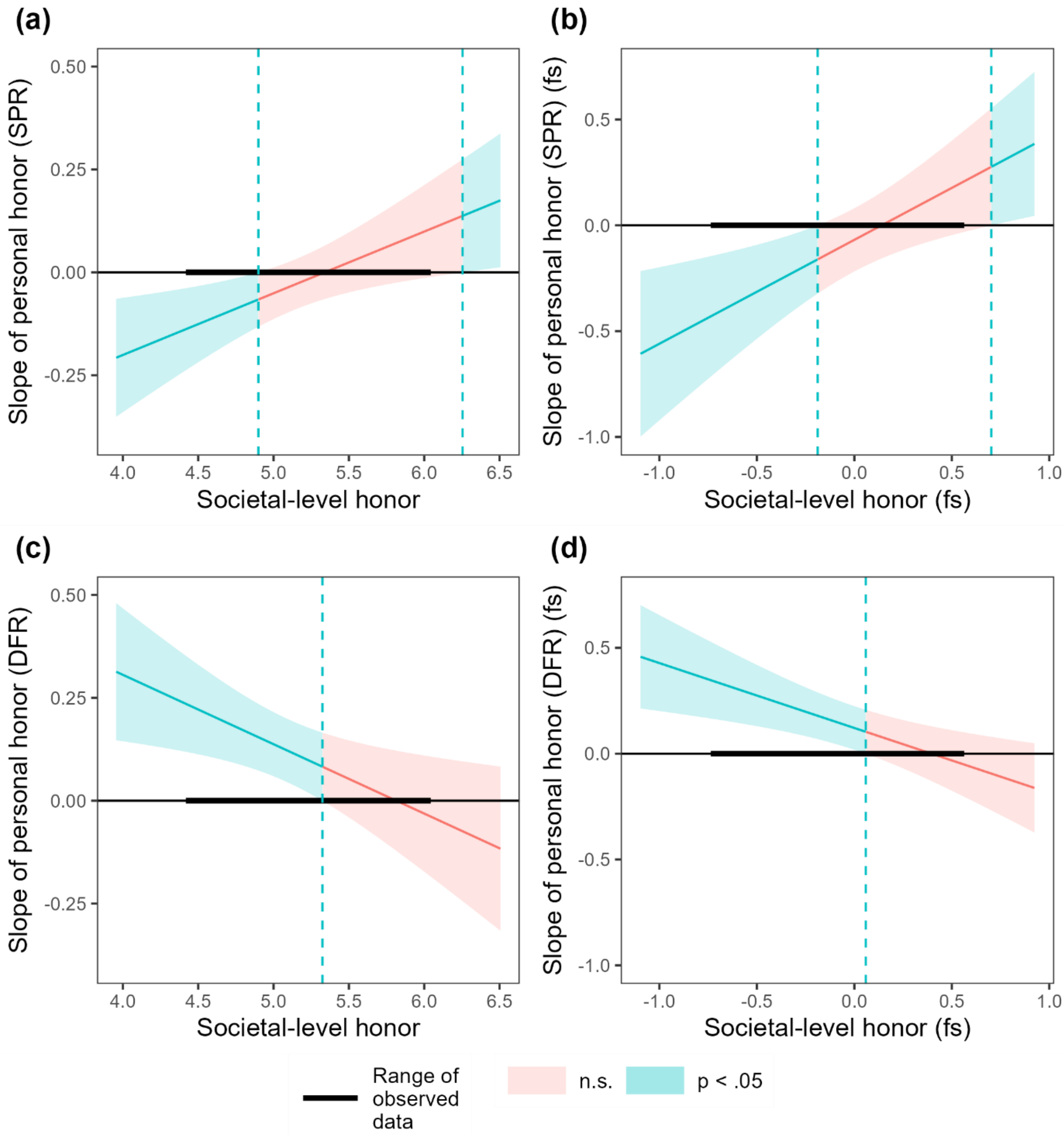

Figure S3. Johnson-Neyman plots for the interaction between personal honour values (self-promotion and retaliation, and defence of family reputation) and societal-level honour in predicting cooperation behaviour: (a) and (c) using observed scores for honour values, (b) and (d) using factor scores for honour values.

Note. SPR = self-promotion and retaliation, DFR = defence of family reputation, fs = factor scores. The x-axis shows the moderator societal-level honor, and y-axis shows the size of the estimated slope of each facet of personal honor values on cooperation behaviour. The range of observed scores of societal-level honor was [4.44, 6.03], and for factor scores, it was [-0.72, 0.55]. The solid line represents how the slope changes as the moderator (i.e., societal-level honor) increases. The shadow area represents the 95% confidence intervals around the slope estimate. The blue region marks the range of moderator values where the slope was statistically significant ($p < .05$): (a) outside the interval [4.90, 6.25], (b) outside the interval [-0.19, 0.70], (c) outside the interval [5.32, 8.43], (d) outside the interval [0.06, 1.31].

3.3.4 Contextual effects of honour on cooperation and expectations about other's cooperation

In this section, we report the results from additional analyses that were not pre-registered to explore the potential contextual effects of honour on cooperation and expectations about other's cooperation. As for competition (see Supplementary Section 3.2.4 for more information about contextual effects), we applied the same grand mean centring approach to the observed scores of perceived normative and personal honour values at the individual level, and added them (along with control variables such as participant age and gender, partner gender information, and game order) to Model S15a and S16a in Supplementary Section 3.3.1 to examine the contextual effect of societal-level perceived normative honour values on cooperation, controlling for individual level perceived normative and personal honour values.

The results showed that the association between societal mean perceived normative honour values and cooperation found in Model S15a or expectations of other's cooperation found in Model S16a both became nonsignificant after controlling for individual-level grand mean-centred perceived normative and personal honour values (see Model S20a and S20b). This suggested that for individuals with the same level of perceived normative and personal honour values, there was no evidence that those living in societies with higher societal mean perceived normative honour values would engage in greater cooperation or expect more cooperation from others. Comparing the B coefficients in Model S15b ($B = 0.381$, see Supplementary Section 3.3.1) and Model S20a ($B = 0.130$), 66% [= (0.381 – 0.130) / 0.381] of the variation in cooperation explained by societal-level honour could be explained by individual-level honour values, particularly personal endorsement of defence of family reputation ($\beta = 0.065$, $p = .002$) and perceived normative values of self-promotion and retaliation ($\beta = 0.052$, $p = .006$, see Table S20). Similarly, for expectations of other's

cooperation, comparing the B coefficients in Model S16b (*B* = 0.309, see Supplementary Section 3.3.1) and Model S20b (*B* = 0.082), 73% [= (0.309 – 0.082) / 0.309] of the variation explained by societal-level honour could be explained by individual-level honour values, including both perceived normative honour values (SPR: β = 0.036, p = .049; DFR: β = 0.077, $p < .001$) and personally endorsed honour values (SPR: β = –0.052, p = .003; DFR: β = 0.050, p = .012, see Table S20). These findings imply that the cultural contexts characterized by varying levels of honour value prevalence shape individual's engagement in interpersonal cooperation and trust primarily through their perceptions of the prescribed values and norms within those cultural contexts.

Table S20. Mixed-effects models with societal-level and individual-level perceived normative honour values (grand mean centred observed scores) predicting individuals' own cooperative behaviours and expectations of others' cooperation.

	**Model S20a: Cooperation Behaviour**						**Model S20b: Cooperation Expectation**					
Predictors	*B*	*SE*	*t*(*df*)	*p*	*std. Beta*	*std. 95% CI*	*B*	*SE*	*t*(*df*)	*p*	*std. Beta*	*std. 95% CI*
(Intercept)	5.604	0.671	8.349(28)	<0.001	/	/	5.507	0.633	8.703(28)	<0.001	/	/
Societal-level honour	**0.130**	**0.128**	**1.020(12)**	**0.327**	**0.027**	**[-0.031, 0.085]**	**0.082**	**0.120**	**0.685(13)**	**0.506**	**0.018**	**[-0.039, 0.074]**
Normative honour (SPR)	0.095	0.034	2.764(3359)	0.006	0.052	[0.015, 0.088]	0.063	0.032	1.970(3360)	0.049	0.036	[0.000, 0.071]
Normative honour (DFR)	0.066	0.040	1.650(3361)	0.099	0.035	[-0.007, 0.076]	0.140	0.037	3.783(3361)	<0.001	0.077	[0.037, 0.117]
Personal honour (SPR)	-0.036	0.031	-1.155(3245)	0.248	-0.021	[-0.056, 0.014]	-0.085	0.029	-2.986(3254)	0.003	-0.052	[-0.085, -0.018]
Personal honour (DFR)	0.122	0.039	3.128(3357)	0.002	0.065	[0.024, 0.105]	0.092	0.036	2.521(3357)	0.012	0.050	[0.011, 0.090]
Partner gender [male]	-0.019	0.027	-0.725(16853)	0.468	-0.008	[-0.031, 0.014]	0.174	0.027	6.342(16853)	<0.001	0.078	[0.054, 0.102]
Partner gender [unknown]	-0.012	0.027	-0.437(16853)	0.662	-0.005	[-0.028, 0.018]	0.047	0.027	1.713(16853)	0.087	0.021	[-0.003, 0.045]
Participant gender [male]	0.113	0.061	1.849(3352)	0.065	0.049	[-0.003, 0.101]	0.057	0.057	1.000(3352)	0.318	0.026	[-0.025, 0.076]
Age	0.003	0.002	1.542(3354)	0.123	0.021	[-0.006, 0.047]	0.001	0.002	0.727(3354)	0.467	0.009	[-0.016, 0.035]
Game order [SL-CG]	-0.471	0.061	-7.716(3354)	<0.001	-0.204	[-0.256, -0.152]	-0.253	0.057	-4.455(3354)	<0.001	-0.114	[-0.164, -0.064]
**Random Effects**												
σ^2	2.420						2.524					
τ_{00}	2.727 Participant_ID:Society						2.285 Participant_ID:Society					
	0.033 Society						0.030 Society					
ICC	0.533						0.478					
N	3371 Participant_ID						3371 Participant_ID					
	13 Society						13 Society					
Observations	20226						20226					
Marginal R^2 / Conditional R^2	0.030 / 0.547						0.022 / 0.490					

Note. SL = step-level public goods game, CG = contest game, *std. Beta* = standardized regression coefficient, *std. 95% CI* = 95% Confidence Interval of the standardized regression coefficient. The reference level for partner gender was [female], for participant gender was [female], for game order was [CG-SL]. Societal-level honour was the societal mean of perceived normative honour values across both the self-promotion and retaliation, and the defence of family reputation facets of honour, while normative honour (centred at the grand mean) was the individual-level unweighted means across all the scale items measuring perceived normative honour values, including both the self-promotion and retaliation, and the defence of family reputation facets of honour.

3.3.5 Additional explanation for cooperation: Relational mobility

We pre-registered to test whether there are other cultural factors additional to honour values that may explain between-individuals level and between-societies level variation in cooperation. *Relational mobility* is a socio-ecological variable that represents how much freedom and opportunity a society affords individuals to choose and dispose of interpersonal relationships based on personal preference[8]. Low relational mobility societies are characterized by closed networks, and low possibility to change interpersonal relationships and groups. High relational mobility societies are characterized by plenty of opportunities to engage in new friendships based on personal preferences and choices. Past research has found higher cooperation in societies characterized by more flexible and fluid social relations, and that people who perceived their environment to have more opportunities to establish new relationships with strangers were generally more cooperative with strangers[12]. We therefore examined whether relational mobility could explain additional variation in cooperation beyond what was explained by honour values.

At the societal level, relational mobility explained significant variation in cooperation beyond societal-level honour (see Model S21a). At the individual level, relational mobility also explained additional variance in cooperation beyond honour values (see Models S21b). Relational mobility was associated with higher cooperation at both societal and individual level. Yet, these results were not replicated using factor scores for honour values and relational mobility (see Model S22a and S22b). The present dataset included only 13 societies, and the mixed-effects models incorporated two societal-level predictors (see Models S21a and S22a). The relatively small sample size at the societal-level may limit the statistical power and generalizability of these findings. These results suggested that relational mobility may provide additional explanation for cooperation beyond honour values,

particularly at the individual level, while interpretations of societal-level patterns should be made cautiously due to potential overfitting and limited degrees of freedom.

Table S21. Mixed-effects models with societal-level and individual-level honour values and relational mobility (observed scores) predicting cooperation.

	**Model S21a: Cooperation Behaviour**						**Model S21b: Cooperation Behaviour**					
Predictors	*B*	*SE*	*t(df)*	*p*	*std. Beta*	*std. 95% CI*	*B*	*SE*	*t(df)*	*p*	*std. Beta*	*std. 95% CI*
(Intercept)	-0.174	1.771	-0.098(10)	0.924	/	/	-0.158	1.800	-0.088(34)	0.930	/	/
Societal-level honour	0.342	0.102	3.337(10)	0.008	0.071	[0.024, 0.119]	0.348	0.104	3.344(10)	0.008	0.073	[0.024, 0.121]
Societal-level RMOB	**1.231**	**0.467**	**2.635(10)**	**0.025**	**0.056**	**[0.009, 0.104]**	1.233	0.474	2.600(10)	0.027	0.056	[0.008, 0.105]
Normative honour (SPR)							0.103	0.034	2.982(3350)	0.003	0.052	[0.018, 0.086]
Normative honour (DFR)							0.049	0.040	1.227(3350)	0.220	0.024	[-0.014, 0.062]
Personal honour (SPR)							-0.015	0.032	-0.486(3350)	0.627	-0.008	[-0.042, 0.025]
Personal honour (DFR)							0.107	0.039	2.714(3350)	0.007	0.051	[0.014, 0.089]
Relational mobility							**0.139**	**0.059**	**2.379(3350)**	**0.017**	**0.033**	**[0.006, 0.061]**
Partner gender [male]							-0.019	0.027	-0.725(16853)	0.468	-0.008	[-0.031, 0.014]
Partner gender [unknown]							-0.012	0.027	-0.437(16853)	0.662	-0.005	[-0.028, 0.018]
Participant gender [male]							0.119	0.061	1.947(3351)	0.052	0.052	[-0.000, 0.104]
Age							0.003	0.002	1.557(3353)	0.120	0.021	[-0.005, 0.047]
Game order [SL-CG]							-0.473	0.061	-7.754(3355)	<0.001	-0.205	[-0.257, -0.153]
**Random Effects**												
σ^2	2.420						2.420					
τ_{00}	2.846 $_{\text{Participant\_ID:Society}}$						2.723 $_{\text{Participant\_ID:Society}}$					
	0.018 $_{\text{Society}}$						0.020 $_{\text{Society}}$					
ICC	0.542						0.531					
N	3371 $_{\text{Participant\_ID}}$						3371 $_{\text{Participant\_ID}}$					
	13 $_{\text{Society}}$						13 $_{\text{Society}}$					
Observations	20226						20226					
Marginal R^2 / Conditional R^2	0.009 / 0.546						0.034 / 0.547					

Note. RMOB = relational mobility, SPR = self-promotion and retaliation, DFR = defence of family reputation, SL = step-level public goods game, CG = contest game, *std. Beta* = standardized regression coefficient, *std. 95% CI* = 95% Confidence Interval of the standardized regression coefficient. The reference level for partner gender was [female], for participant gender was [female], for game order was [CG-SL]. Societal-level honour was the societal mean of perceived normative honour values across both the SPR and DFR facets of honour, and societal-level RMOB was the societal mean of relational mobility, while personal honour, normative honour and relational mobility (centred within society) were the individual-level unweighted means across the scale items.

Table S22. Mixed-effects models with societal-level and individual-level honour values and relational mobility (factor scores) predicting cooperation.

	**Model S22a: Cooperation Behaviour**						**Model S22b: Cooperation Behaviour**					
Predictors	*B*	*SE*	*t*(*df*)	*p*	*std. Beta*	*std. 95% CI*	*B*	*SE*	*t*(*df*)	*p*	*std. Beta*	*std. 95% CI*
(Intercept)	6.217	0.061	101.297(10)	<0.001	/	/	6.234	0.118	52.736(24)	<0.001	/	/
Societal-level honour (fs)	0.321	0.170	1.890(10)	0.088	0.052	[-0.009, 0.114]	0.326	0.173	1.886(10)	0.089	0.053	[-0.010, 0.115]
Societal-level RMOB (fs)	**0.728**	**0.400**	**1.821(10)**	**0.099**	**0.050**	**[-0.011, 0.112]**	0.722	0.406	1.776(10)	0.106	0.050	[-0.013, 0.112]
Normative honour (SPR) (fs)							0.154	0.073	2.129(3352)	0.033	0.042	[0.003, 0.080]
Normative honour (DFR) (fs)							-0.000	0.061	-0.001(3351)	0.999	-0.000	[-0.044, 0.044]
Personal honour (SPR) (fs)							-0.059	0.077	-0.772(3350)	0.440	-0.012	[-0.043, 0.019]
Personal honour (DFR) (fs)							0.109	0.053	2.052(3351)	0.040	0.038	[0.002, 0.074]
Relational mobility (fs)							**0.188**	**0.135**	**1.394(3351)**	**0.163**	**0.020**	**[-0.008, 0.049]**
Partner gender [male]							-0.019	0.027	-0.725(16853)	0.468	-0.008	[-0.031, 0.014]
Partner gender [unknown]							-0.012	0.027	-0.437(16853)	0.662	-0.005	[-0.028, 0.018]
Participant gender [male]							0.124	0.062	2.012(3351)	0.044	0.054	[0.001, 0.106]
Age							0.004	0.002	1.961(3352)	0.050	0.027	[0.000, 0.053]
Game order [SL-CG]							-0.480	0.061	-7.827(3353)	<0.001	-0.208	[-0.260, -0.156]
**Random Effects**												
σ^2	2.420						2.420					
τ_{00}	2.846 Participant_ID:Society						2.758 Participant_ID:Society					
	0.036 Society						0.038 Society					
ICC	0.544						0.536					
N	3371 Participant_ID						3371 Participant_ID					
	13 Society						13 Society					
Observations	20226						20226					
Marginal R^2 / Conditional R^2	0.007 / 0.547						0.024 / 0.547					

Note. fs = factor score, RMOB = relational mobility, SPR = self-promotion and retaliation, DFR = defence of family reputation, SL = step-level public goods game, CG = contest game, *std. Beta* = standardized regression coefficient, *std. 95% CI* = 95% Confidence Interval of the standardized regression coefficient. The reference level for partner gender was [female], for participant gender was [female], for game order was [CG-SL]. Societal-level honour (fs) was the societal-level factor scores of perceived normative honour values across both the SPR and DFR facets of honour, and societal-level RMOB (fs) was the societal-level factor scores of relational mobility, while personal honour (fs), normative honour (fs) and relational mobility (fs) were the individual-level factor scores.

3.3.6 Other societal-level indicators and cooperation

In this section, we report the results of a series of mixed effects models, where each model included one societal-level indicator predicting cooperation (see Table S23). The same set of societal-level indicators were examined in relation to cooperation that we had previously examined in relation to competition (see Section 3.2.5 for societal-level indicators and competition, and Table S13 for more information about the operationalization of these societal-level indicators). The results showed that among all these societal-level indicators, only market competitiveness was negatively associated with cooperation ($\beta = -.077$, $p = .022$, see Table S23). Interestingly, intercorrelations showed that market competitiveness was the strongest societal-level indicator that negatively correlated with societal-level honour, compared to other indicators (see Figure S4a and S4b). The Global Competitiveness Index (GCI) that has been used to operationalize market competitiveness is a highly complex indicator assessing the ability of countries to provide high levels of prosperity to their citizens. Thus, these findings seem to suggest that greater interpersonal cooperation occurred in harsher environments with lower economic productivity and prosperity, where a stronger culture of honour may exist. Considering the results from both competition and cooperation, our findings may imply that harsher environments may require individuals to develop both the ability to cooperate and compete for scarce resources[13] (see Section 3.2.6 for analyses on the association between other societal-level indicators and competition).

Table S23. A series of mixed-effects models for each societal-level indicator predicting cooperation in separate models.

		Models S23a-S23n: Cooperation Behaviour								
#	**Societal-level indicator**	***B***	***SE***	***t(df)***	***p***	***std. Beta***	***std. 95%CI***	$N_{society}$	$N_{participant}$	$N_{observation}$
	Economic indicators									
a	GDP per capita	-0.112	0.082	-1.363(10)	0.203	-0.046	[-0.113, 0.020]	12	3126	18756
b	GNI	-0.134	0.079	-1.703(10)	0.119	-0.056	[-0.120, 0.008]	12	3126	18756
c	Human development index	-0.101	0.084	-1.211(10)	0.254	-0.042	[-0.110, 0.026]	12	3126	18756
d	Gender inequality	0.112	0.082	1.360(10)	0.204	0.047	[-0.021, 0.114]	12	3126	18756
	Institutions									
e	Government effectiveness	-0.121	0.081	-1.490(10)	0.167	-0.050	[-0.116, 0.016]	12	3126	18756
f	Rule of law	-0.035	0.089	-0.393(10)	0.703	-0.014	[-0.087, 0.058]	12	3126	18756
g	Political stability	0.015	0.089	0.166(10)	0.871	0.006	[-0.067, 0.079]	12	3126	18756
h	Corruption control	-0.048	0.088	-0.543(10)	0.599	-0.020	[-0.092, 0.052]	12	3126	18756
i	Corruption perceptions index	-0.159	0.074	-2.158(10)	0.056	-0.066	[-0.126, -0.006]	12	3126	18756
j	Market competitiveness	-0.184*	0.068	-2.717(10)	0.022	-0.077	[-0.132, -0.021]	12	3126	18756
	Historical and ecological threats									
k	Historical prevalence of infectious disease	0.040	0.089	0.454(10)	0.660	0.017	[-0.055, 0.089]	12	3126	18756
l	World risk index	0.041	0.089	0.463(10)	0.653	0.017	[-0.055, 0.089]	12	3126	18756
m	Exposure	-0.125	0.080	-1.557(10)	0.150	-0.052	[-0.117, 0.013]	12	3126	18756
n	Vulnerability	0.127	0.080	1.591(10)	0.143	0.053	[-0.012, 0.119]	12	3126	18756

Note. std. Beta = standardized regression coefficient, *std. 95% CI* = 95% Confidence Interval of the standardized regression coefficient, $N_{society}$ = number of societies, $N_{participant}$ = number of participants, $N_{observation}$ = number of observations at the decision level. Unstandardized regression coefficients (*B*) were marked with an asterisk if they were significant at the 0.05 level. See Table S13 for the operationalization for each societal-level indicator.

3.3.7 Re-analyses of existing datasets on cooperation

As pre-registered, we conducted secondary analyses of existing datasets to explore whether societal-level honour relates to cooperation versus non-cooperation in situations where non-cooperation can always yield the best outcome for an individual regardless of what others do (i.e., prisoner's dilemma, public goods dilemma). We applied societal mean perceived normative honour values as a societal-level indicator to predict (a) study-level mean cooperation rates in mixed-effects meta-regression models, and (b) individual's cooperation decisions and expectations of others' cooperation in mixed-effects linear regression models across societies. To do so, societal-level honour indicators (operationalized as the societal mean perceived normative honour values) were retrieved from Study 2 of a recent paper[6] [originally cited as Kirchner-Häusler et al. (in preparation) in the pre-registration], which used the same measurement tool to assess perceived normative honour values across diverse samples, including participants beyond university students, varying by age and gender. In their study, two items were initially used to assess perceived normative honour values but were not provided in their final dataset based on model selection results (i.e., "*People must always be ready to defend their honour*", and "*It is important to promote oneself to others*").

We then retrieved (a) study-level (and treatment-level) data on mean cooperation rates from samples in social dilemma studies using prisoner's dilemmas and public goods dilemmas, conducted in eight societies (Turkey, Greece, Italy, Spain, Japan, South Korea, the U.K., the U.S.) in the past literature from a meta-analysis conducted by Spadaro and colleagues[14], and (b) individual decisions on cooperation (measured using a prisoner's dilemma) and expectations about other's cooperation across nine societies (Egypt, Turkey, Greece, Italy, Spain, Japan, South Korea, the U.K., the U.S.) from an empirical study

conducted by Romano and colleagues[12]. These were the only societies that overlapped with the societies in the societal-level honour indicator dataset.

We started by exploring whether there was cross-societal variation in study-level (and treatment-level) mean cooperation rates among the eight societies that we selected from the meta-analysis conducted by Spadaro and colleagues[14] to match with the societies with honour values available. We conducted multilevel meta-analyses to evaluate variance at the between-societies level by fitting a baseline model with society as a random intercept and a second model with the between-societies variance set to zero. Model comparison results revealed that removing the between-societies level variance even resulted in a slightly higher AIC and BIC, with a non-significant likelihood ratio test (LRT = 3.37, p = .067), suggesting that between-societies level variance was not a strong contributor to the overall variability in effect sizes. We therefore ran a mixed-effects meta-regression model with study as the only random intercept, using societal mean perceived normative honour values to predict study-level (and treatment-level) logit-transformed cooperation rates. The model controlled for a set of study characteristics (i.e., symmetry, repetitions, group size, K index, communication, decision protocol, sanction, choice options, source of society, and period of cooperation)[14]. However, we found no support for the association between social-level honour and cooperation (b = 0.061, p = .487, Δ pseudo R^2 = 0%, see Table S24).

Running a multilevel meta-analysis with only eight observations for societies, included as a random intercept, risks producing biased variance estimates and inflated Type I error, making the results less reliable and generalizable. To address this limitation, we conducted a non-pre-registered robustness check using a larger sample of societies from the meta-analytic dataset, categorizing them into five world regions [i.e., *Middle-Eastern*: Iran, Israel, Oman, Palestine (Palestinian Territories), Saudi Arabia, Turkey; *Latin American*: Argentina, Bolivia, Brazil, Chile, Colombia, Costa Rica, Guatemala, Mexico, Peru, Uruguay,

Venezuela; *South Asian*: India; *East Asian*: China, Hong Kong (a Special Administrative Region of China), Japan, South Korea, Taiwan; *Anglo-Western*: Australia, Canada, United Kingdom, New Zealand, United States]. Past literature has found honour being a prevalent cultural value in world regions such as Middle East, Latin America and South Asia. We therefore recoded society into three regions including East Asia, Anglo-West, and a third category representing honour cultures. We included two dummy variables for region as predictors in the mixed-effects meta-regression model (dummy 1: honour cultures = 0, East Asia = 1; dummy 2: honour cultures = 0, Anglo-West = 1). However, we still found no evidence that region was associated with cooperation (dummy 1: $b = -0.061$, $p = .599$; dummy 2, $b = 0.009$, $p = .935$, Δ pseudo $R^2 = 0\%$, see Table S25).

Lastly, we explored whether there was cross-society variation in cooperation with unrelated others from one's own society and expectations about other's cooperation among the nine societies retrieved from the study conducted by Romano and colleagues[12]. We assessed the between-society variance on cooperation and expectations by first fitting models with participant as a random intercept, then adding society to the random intercept, and comparing these models to see if the added variance improved the model fit. Results showed a significant improvement in model fit on cooperation ($\chi^2 = 75.04$, $p < .001$) and expectation ($\chi^2 = 73.36$, $p < .001$), indicating the existence of cross-society variation in cooperation and expectations. We then applied societal mean perceived normative honour values from Study 2 of a recent paper[6] to predict cooperation and expectation of other's cooperation in mixed effect models, with participant and society entered as random intercepts. Results showed that societal mean perceived normative honour values did not significantly predict cooperation or expectation towards strangers from one's own society (*p*s > .695, see Table S26). All together, these findings suggested that societal-level honour was not linked to cooperation (or

expectations of others' cooperation) measured in prisoner's dilemmas and continuous public goods games.

Table S24. Mixed-effects meta-regression model with societal-level honour (societal mean perceived normative honour values) predicting study-level (and treatment-level) logit-transformed cooperation rates.

	**Model S24: Logit-transformed cooperation rates**				
Predictors	*B*	*SE*	*95% CI*	*t*	*p*
(Intercept)	-0.667	0.187	-1.034 – -0.301	-3.570	<0.001
Symmetry [mixed]	0.038	0.245	-0.444 – 0.519	0.153	0.878
Symmetry [symmetric]	0.257	0.135	-0.007 – 0.521	1.910	0.056
Repetitions [mixed]	-0.152	0.267	-0.675 – 0.371	-0.569	0.569
Repetitions [one-shot]	0.012	0.055	-0.096 – 0.119	0.213	0.832
Group size log	-0.050	0.055	-0.157 – 0.058	-0.901	0.368
K Index	**0.792**	**0.118**	**0.562 – 1.023**	**6.738**	**<0.001**
Communication [mixed]	0.524	0.295	-0.055 – 1.103	1.776	0.076
Communication [present]	**0.513**	**0.072**	**0.371 – -0.655**	**7.085**	**<0.001**
Decision protocol [mixed]	-0.112	0.205	-0.514 – 0.290	-0.546	0.585
Decision protocol [simultaneous]	-0.046	0.096	-0.233 – 0.142	-0.479	0.632
Sanction [mixed]	0.335	0.233	-0.121 – 0.791	1.441	0.150
Sanction [present]	**0.508**	**0.072**	**0.367 – 0.648**	**7.077**	**<0.001**
Choice options [continuous]	0.034	0.064	-0.091 – 0.159	0.535	0.593
Source of society [different societies]	-0.328	0.284	-0.884 – 0.229	-1.156	0.248
Source of society [all authors]	-0.094	0.072	-0.236 – 0.048	-1.298	0.195
Source of society [most authors]	0.308	0.287	-0.255 – 0.872	1.074	0.283
Period of cooperation [first]	0.207	0.172	-0.130 – 0.544	1.205	0.229
Society-level honour	0.061	0.088	-0.111 – 0.234	0.696	0.487
**Model Statistics**					
$Q_{\text{residual}}(df)$	26114.83 (1151)*				
$F_{\text{moderators}}(df1, df2)$	8.97 (18, 1151)*				
τ^2	0.202 $_{\text{Effect Size:Study}}$				
	0.318 $_{\text{Study}}$				
I^2: between-study level (%)	37.75				
I^2: within-study level (%)	59.31				
N	1170 $_{\text{Effect Size}}$				
	740 $_{\text{Study}}$				

Note. Group size log, K index and societal-level honour are continuous variables, others are dummy variables. The reference level for symmetry was asymmetric, for repetitions was repeated, for communication was absent, for decision protocol was sequential, for sanction was absent, for choice options was dichotomous, for source of society was specified in the text, and for period of cooperation was from all periods. See the meta-analysis conducted by Spadaro and colleagues[14] for more information about the coding of these study characteristics. Effect Size: Study = effect sizes were nested within studies.

Table S25. Mixed-effects meta-regression model with region predicting study-level (and treatment-level) logit-transformed cooperation rates.

	**Model S25: Logit-transformed cooperation rates**				
Predictors	*B*	*SE*	*95% CI*	*t*	*p*
(Intercept)	-0.502	0.190	-0.876 – -0.129	-2.641	0.008
Symmetry [mixed]	-0.085	0.219	-0.515 – 0.344	-0.389	0.697
Symmetry [yes]	0.098	0.117	-0.131 – 0.326	0.837	0.403
Repetitions [mixed]	0.025	0.228	-0.422 – 0.472	0.110	0.913
Repetitions [one-shot]	0.018	0.048	-0.076 – 0.113	0.378	0.705
Group size log	-0.041	0.047	-0.134 – 0.052	-0.873	0.383
K Index	**0.746**	**0.108**	**0.533 – 0.959**	**6.880**	**<0.001**
Communication [mixed]	**0.575**	**0.235**	**0.113 – 1.037**	**2.442**	**0.015**
Communication [present]	**0.494**	**0.070**	**0.358 – 0.631**	**7.090**	**<0.001**
Decision protocol [mixed]	-0.185	0.180	-0.537 – 0.168	-1.028	0.304
Decision protocol [simultaneous]	-0.100	0.088	-0.273 – 0.073	-1.137	0.256
Sanction [mixed]	0.096	0.193	-0.283 – 0.475	0.497	0.619
Sanction [present]	**0.485**	**0.060**	**0.368 – 0.602**	**8.140**	**<0.001**
Choice options [continuous]	0.088	0.056	-0.022 – 0.197	1.576	0.115
Source of society [different societies]	-0.378	0.271	-0.910 – 0.153	-1.395	0.163
Source of society [all authors]	-0.067	0.066	-0.197 – 0.063	-1.011	0.312
Source of society [most authors]	0.197	0.258	-0.309 – 0.702	0.764	0.445
Period of cooperation [first]	0.155	0.156	-0.150 – 0.460	0.994	0.320
Region [East Asia]	-0.061	0.116	-0.288 – 0.166	-0.527	0.599
Region [Anglo West]	0.009	0.107	-0.200 – 0.218	0.082	0.935
**Model Statistics**					
$Q_{\text{residual}}(df)$	36810.05 (1343)*				
$F_{\text{moderators}}(df1, df2)$	9.54 (19, 1343)*				
τ^2	0.197 $_{\text{EffectSize:Study}}$				
	0.284 $_{\text{Study}}$				
I^2: between-study level (%)	39.93				
I^2: within-study level (%)	57.56				
N	1363 $_{\text{EffectSize}}$				
	880 $_{\text{Study}}$				

Note. Group size log, K index and societal-level honour are continuous variables, others are dummy variables. The reference level for symmetry was asymmetric, for repetitions was repeated, for communication was absent, for decision protocol was sequential, for sanction was absent, for choice options was dichotomous, for source of society was specified in the text, and for period of cooperation was from all periods, for region was societies representing honour cultures. See the meta-analysis conducted by Spadaro and colleagues[14] for more information about the coding of these study characteristics. Effect Size: Study = effect sizes were nested within studies.

Table S26. Mixed-effects models with societal-level honour (societal mean perceived normative honour values) predicting cooperation with unrelated others from one's own society and expectations about other's cooperation.

	**Model S26a: Cooperation Behaviour**						**Model S26b: Cooperation Expectation**					
Predictors	*B*	*SE*	*t*(*df*)	*p*	*std. Beta*	*std. 95% CI*	*B*	*SE*	*t*(*df*)	*p*	*std. Beta*	*std. 95% CI*
(Intercept)	4.356	0.158	27.503(7)	<0.001	/	/	4.241	0.155	27.320(7)	<0.001	/	/
Society-level honour	0.073	0.186	0.392(7)	0.707	0.021	[-0.107, 0.149]	-0.014	0.183	-0.078(7)	0.940	-0.004	[-0.134, 0.126]
**Random Effects**												
σ^2	2.099						2.126					
τ_{00}	5.016 $_{\text{Participant\_ID:Society}}$						4.486 $_{\text{Participant\_ID:Society}}$					
	0.186 $_{\text{Society}}$						0.180 $_{\text{Society}}$					
ICC	0.713						0.687					
N	3872 $_{\text{Participant\_ID}}$						3872 $_{\text{Participant\_ID}}$					
	9 $_{\text{Society}}$						9 $_{\text{Society}}$					
Observations	15240						15240					
Marginal R^2 / Conditional R^2	0.000 / 0.713						0.000 / 0.687					

Note. *std. Beta* = standardized regression coefficient. Societal-level honour was the societal mean of perceived normative honour values across both the SPR and DFR facets of honour.

3.3.8 Honour and coordinative decisions (contributing 8 or 6 MUs)

In this section, we present the results from models exploring the association between societal-level honour (see Model S27a and S27c) or individual-level honour values (see Model S27b and S27d) and the likelihood of contributing 8 MUs or contributing 6 MUs, using both observed scores and factor scores of honour values (Table S27-S28). Although society was removed from the models as a random intercept due to statistically insignificant variance at the between-society level, we still found that societal-level honour was positively associated with the likelihood of contributing 8 MUs (see Model S27a and Model S28a). Perceived normative values of defence of family reputation consistently positively predicted the likelihood of contributing 8 MUs (see Model S27b and S28b), but not 6 MUs (see Model S27d and S28d). However, perceived normative values of self-promotion and retaliation were not associated with contributing either 8 or 6 MUs. Personal values of defence of family reputation consistently positively predicted the likelihood of contributing 6 MUs (see Model S27d and S28d), but not with 8 MUs (see Model S27b and S28b). In contrast, personally endorsing values of self-promotion and retaliation consistently and negatively predicted the likelihood of contributing either 8 or 6 MUs.

Table S27. Generalized linear mixed models with personal and perceived normative honour values (observed scores) predicting the likelihood of contributing 8 MUs or 6 MUs.

	**Model S27a: Contribute 8 MUs**			**Model S27b: Contribute 8 MUs**			**Model S27c: Contribute 6 MUs**			**Model S27d: Contribute 6 MUs**		
Predictors	*Odds Ratios*	*95% CI*	*p*	*Odds Ratios*	*95% CI*	*p*	*Odds Ratios*	*95% CI*	*p*	*Odds Ratios*	*95% CI*	*p*
(Intercept)	0.102	0.094 – 0.112	<0.001	0.113	0.098 – 0.131	<0.001	0.182	0.171 – 0.194	<0.001	0.169	0.151 – 0.190	<0.001
Societal-level honour	**1.138**	**1.055 – 1.227**	**0.001**	1.145	1.063 – 1.233	<0.001	**0.994**	**0.937 – 1.055**	**0.841**	0.996	0.939 – 1.057	0.893
Normative honour (SPR)				**1.039**	**0.947 – 1.141**	**0.419**				**1.010**	**0.938 – 1.089**	**0.785**
Normative honour (DFR)				**1.300**	**1.168 – 1.446**	**<0.001**				**1.052**	**0.968 – 1.143**	**0.230**
Personal honour (SPR)				**0.837**	**0.765 – 0.915**	**<0.001**				**0.881**	**0.820 – 0.947**	**0.001**
Personal honour (DFR)				**1.006**	**0.909 – 1.115**	**0.904**				**1.137**	**1.048 – 1.233**	**0.002**
Partner gender [male]				0.982	0.885 – 1.089	0.730				0.955	0.870 – 1.047	0.323
Partner gender [unknown]				0.934	0.841 – 1.037	0.200				0.984	0.897 – 1.078	0.725
Participant gender [male]				1.271	1.101 – 1.467	0.001				1.011	0.902 – 1.133	0.852
Age				1.056	0.982 – 1.136	0.140				1.035	0.977 – 1.096	0.247
Game order [SL-CG]				0.684	0.593 – 0.790	<0.001				1.187	1.059 – 1.329	0.003
**Random Effects**												
σ^2	3.290			3.290			3.29			3.290		
τ_{00}	2.488 Participant_ID			2.367 Participant_ID			1.420 Participant_ID			1.395 Participant_ID		
ICC	0.431			0.418			0.301			0.298		
N	3371 Participant_ID			3371 Participant_ID			3371 Participant_ID			3371 Participant_ID		
Observations	20226			20226			20226			20226		
Marginal R^2 / Conditional R^2	0.003 / 0.432			0.027 / 0.434			0.000 / 0.301			0.008 / 0.303		

Note. SPR = self-promotion and retaliation, DFR = defence of family reputation, SL = step-level public goods game, CG = contest game, *std. Beta* = standardized regression coefficient. The reference level for partner gender was [female], for participant gender was [female], for game order was [CG-SL]. Societal-level honour was the societal mean of perceived normative honour values across both the SPR and DFR facets of honour, while personal honour and normative honour (centred within society) were the individual-level unweighted means across the scale items. Society was not entered to the models as a random intercept due to statistically insignificant variance at the between-society level.

Table S28. Generalized linear mixed models with personal and perceived normative honour values (factor scores) predicting the likelihood of contributing 8 MUs or 6 MUs.

	**Model S28a: Contribute 8 MUs**			**Model S28b: Contribute 8 MUs**			**Model S28c: Contribute 6 MUs**			**Model S28d: Contribute 6 MUs**		
Predictors	*Odds Ratios*	*95% CI*	*p*	*Odds Ratios*	*95% CI*	*p*	*Odds Ratios*	*95% CI*	*p*	*Odds Ratios*	*95% CI*	*p*
(Intercept)	0.102	0.094 – 0.112	<0.001	0.114	0.098 – 0.131	<0.001	0.182	0.171 – 0.194	<0.001	0.170	0.151 – 0.191	<0.001
Societal-level honour (fs)	**1.547**	**1.274 – 1.878**	**<0.001**	1.542	1.274 – 1.866	<0.001	**1.029**	**0.884 – 1.198**	**0.709**	1.025	0.881 – 1.193	0.750
Normative honour (SPR) (fs)				**1.089**	**0.921 – 1.288**	**0.318**				**1.044**	**0.913 – 1.193**	**0.531**
Normative honour (DFR) (fs)				**1.352**	**1.173 – 1.559**	**<0.001**				**1.078**	**0.964 – 1.205**	**0.190**
Personal honour (SPR) (fs)				**0.768**	**0.644 – 0.915**	**0.003**				**0.865**	**0.751 – 0.996**	**0.044**
Personal honour (DFR) (fs)				**1.054**	**0.933 – 1.191**	**0.398**				**1.198**	**1.086 – 1.321**	**<0.001**
Partner gender [male]				0.982	0.885 – 1.089	0.730				0.954	0.870 – 1.047	0.323
Partner gender [unknown]				0.934	0.841 – 1.037	0.200				0.984	0.897 – 1.078	0.725
Participant gender [male]				1.261	1.093 – 1.456	0.001				1.005	0.897 – 1.126	0.927
Age				1.050	0.977 – 1.129	0.184				1.028	0.971 – 1.089	0.341
Game order [SL-CG]				0.684	0.593 – 0.789	<0.001				1.186	1.059 – 1.329	0.003
**Random Effects**												
σ^2	3.290			3.290			3.290			3.290		
τ_{00}	2.481 $_{\text{Participant\_ID}}$			2.348 $_{\text{Participant\_ID}}$			1.420 $_{\text{Participant\_ID}}$			1.389 $_{\text{Participant\_ID}}$		
ICC	0.430			0.416			0.301			0.297		
N	3371 $_{\text{Participant\_ID}}$			3371 $_{\text{Participant\_ID}}$			3371 $_{\text{Participant\_ID}}$			3371 $_{\text{Participant\_ID}}$		
Observations	20226			20226			20226			20226		
Marginal R^2 / Conditional R^2	0.005 / 0.433			0.031 / 0.435			0.000 / 0.302			0.010 / 0.304		

Note. fs = factor score, SPR = self-promotion and retaliation, DFR = defence of family reputation, SL = step-level public goods game, CG = contest game, *std. Beta* = standardized regression coefficient. The reference level for partner gender was [female], for participant gender was [female], for game order was [CG-SL]. Societal-level honour (fs) was the societal-level factor scores of perceived normative honour values across both the SPR and DFR facets of honour, while personal honour (fs) and normative honour (fs) were the individual-level factor scores. Society was not entered to the models as a random intercept due to statistically insignificant variance at the between-society level.

3.4 Honour and anticipation of coordination success

In this section, we present the results from additional exploratory analyses examining the sum of an individual's own investment and expected partner's investment in a given round. In the step-level public goods game, we categorized a given game round as *efficient coordination* if the expected sum contribution reached the second provision point (i.e., 16 MUs), as *less-efficient* coordination if it only reaches the first provision point (i.e., 12 MUs), and otherwise *failed coordination*.

We present the results from models exploring the association between societal-level honour or individual-level honour values and the occurrence of anticipated success of efficient coordination or less-efficient coordination (i.e., the likelihood of a game round being categorized as efficient coordination success or less-efficient coordination success). Although society was removed as a random intercept in models testing efficient coordination success due to statistically nonsignificant variance at the between-society level, we consistently found that societal-level honour positively predicted the occurrence of efficient coordination success using both observed scores (see Model S29a) and factor scores (see Model S30a) of honour values. However, no association was found between societal-level honour and less-efficient coordination success (see Model S29c and S30c). At the individual-level, perceived normative values of defence of family reputation were positively associated with the occurrence of anticipated less-efficient coordination success (i.e., reaching the first provision point, see Model S29d and S30d). Similarly, personally endorsing values of defence of family reputation was positively associated with anticipated coordination success, but specifically for efficient coordination (i.e., reaching the second provision point, see Model S29b and S30b). Conversely, personal values of self-promotion and retaliation were consistently negatively associated with the occurrence of less-efficient coordination success (see Model S29d and S30d).

Table S29. Generalized linear mixed models with personal and perceived normative honour values (observed scores) predicting the likelihood of a game round being categorized as anticipated success of efficient or less-efficient coordination.

	**Model S29a: Efficient coordination**			**Model S29b: Efficient coordination**			**Model S29c: Less-efficient coordination**			**Model S29d: Less-efficient coordination**		
Predictors	*Odds Ratios*	*95% CI*	*p*	*Odds Ratios*	*95% CI*	*p*	*Odds Ratios*	*95% CI*	*p*	*Odds Ratios*	*95% CI*	*p*
(Intercept)	0.063	0.054 – 0.073	<0.001	0.034	0.026 – 0.044	<0.001	0.492	0.448 – 0.539	<0.001	0.581	0.497 – 0.678	<0.001
Societal-level honour	**1.418**	**1.256 – 1.600**	**<0.001**	1.462	1.301 – 1.643	<0.001	**1.011**	**0.919 – 1.113**	**0.816**	1.001	0.903 – 1.109	0.988
Normative honour (SPR)				**1.151**	**0.996 – 1.329**	**0.056**				**1.048**	**0.964 – 1.139**	**0.273**
Normative honour (DFR)				**1.095**	**0.932 – 1.285**	**0.270**				**1.204**	**1.097 – 1.322**	**<0.001**
Personal honour (SPR)				**0.923**	**0.803 – 1.061**	**0.258**				**0.838**	**0.773 – 0.909**	**<0.001**
Personal honour (DFR)				**1.190**	**1.017 – 1.392**	**0.030**				**1.063**	**0.970 – 1.164**	**0.190**
Cooperation deviation type [conditional cooperation]				6.873	5.881 – 8.032	<0.001				0.670	0.605 – 0.741	<0.001
Cooperation deviation type [unconditional cooperation]				1.171	0.998 – 1.373	0.052				1.148	1.043 – 1.263	0.005
Partner gender [male]				1.333	1.183 – 1.502	<0.001				0.936	0.863 – 1.017	0.117
Partner gender [unknown]				1.061	0.941 – 1.197	0.335				0.998	0.920 – 1.083	0.961
Participant gender [male]				1.284	1.026 – 1.607	0.029				1.000	0.880 – 1.137	0.999
Age				1.033	0.921 – 1.158	0.581				0.955	0.896 – 1.020	0.167
Game order [SL-CG]				0.450	0.359 – 0.564	<0.001				0.879	0.774 – 0.999	0.049
**Random Effects**												
σ^2	3.290			3.290			3.290			3.290		
τ_{00}	7.745 $_{\text{Participant\_ID}}$			6.982 $_{\text{Participant\_ID}}$			2.545 $_{\text{Participant\_ID:Society}}$			2.473 $_{\text{Participant\_ID:Society}}$		
	N/A			N/A			0.014 $_{\text{Society}}$			0.019 $_{\text{Society}}$		
ICC	0.702			0.680			0.438			0.431		
N	3371 $_{\text{Participant\_ID}}$			3371 $_{\text{Participant\_ID}}$			3371 $_{\text{Participant\_ID}}$			3371 $_{\text{Participant\_ID}}$		
	N/A			N/A			13 $_{\text{Society}}$			13 $_{\text{Society}}$		
Observations	20226			20226			20226			20226		
Marginal R^2 / Conditional R^2	0.010 / 0.705			0.104 / 0.713			0.000 / 0.438			0.020 / 0.442		

Note. SPR = self-promotion and retaliation, DFR = defence of family reputation, Cooperation deviation type = the type of deviations in an individual's own cooperation from expectations of other's cooperation, SL = step-level public goods game, CG = contest game, *std. Beta* = standardized regression coefficient. The reference level for cooperation deviation type was [underinvested cooperation], for partner gender was [female], for participant gender was [female], for game order was [CG-SL]. Cooperation deviation type was controlled in the model. Societal-level honour was the societal mean of perceived normative honour values across both the SPR and DFR facets of honour, while personal honour and normative honour (centred within society) were the individual-level unweighted means across the scale items. Society was not entered to the models predicting the likelihood of efficient competition as a random intercept due to statistically insignificant variance at the between-society level.

Table S30. Generalized linear mixed models with personal and perceived normative honour values (factor scores) predicting the likelihood of a game round being categorized as anticipated success of efficient or less-efficient coordination.

	**Model S30a: Efficient coordination**			**Model S30b: Efficient coordination**			**Model S30c: Less-efficient coordination**			**Model S30d: Less-efficient coordination**		
Predictors	*Odds Ratios*	*95% CI*	*p*	*Odds Ratios*	*95% CI*	*p*	*Odds Ratios*	*95% CI*	*p*	*Odds Ratios*	*95% CI*	*p*
(Intercept)	0.062	0.054 – 0.073	<0.001	0.034	0.026 – 0.043	<0.001	0.491	0.451 – 0.536	<0.001	0.581	0.500 – 0.676	<0.001
Societal-level honour (fs)	**2.459**	**1.805 – 3.350**	**<0.001**	2.473	1.832 – 3.336	<0.001	**1.175**	**0.934 – 1.478**	**0.169**	1.155	0.903 – 1.476	0.251
Normative honour (SPR) (fs)				**1.234**	**0.952 – 1.601**	**0.112**				**1.094**	**0.942 – 1.272**	**0.240**
Normative honour (DFR) (fs)				**1.040**	**0.837 – 1.293**	**0.722**				**1.242**	**1.095 – 1.408**	**0.001**
Personal honour (SPR) (fs)				**0.911**	**0.693 – 1.199**	**0.506**				**0.773**	**0.660 – 0.905**	**0.001**
Personal honour (DFR) (fs)				**1.224**	**1.013 – 1.480**	**0.036**				**1.103**	**0.989 – 1.230**	**0.079**
Cooperation deviation type [conditional cooperation]				6.842	5.855 – 7.996	<0.001				0.669	0.605 – 0.740	<0.001
Cooperation deviation type [unconditional cooperation]				1.176	1.003 – 1.379	0.046				1.151	1.046 – 1.266	0.004
Partner gender [male]				1.332	1.182 – 1.501	<0.001				0.937	0.863 – 1.017	0.118
Partner gender [unknown]				1.060	0.940 – 1.196	0.341				0.998	0.920 – 1.083	0.962
Participant gender [male]				1.290	1.030 – 1.617	0.027				0.997	0.877 – 1.134	0.968
Age				1.036	0.924 – 1.162	0.541				0.953	0.893 – 1.017	0.147
Game order [SL-CG]				0.447	0.357 – 0.561	<0.001				0.878	0.773 – 0.998	0.046
**Random Effects**												
σ^2	3.290			3.290			3.290			3.290		
τ_{00}	7.746 Participant_ID			7.061 Participant_ID			2.546 Participant_ID:Society			2.466 Participant_ID:Society		
	N/A			N/A			0.011 Society			0.015 Society		
ICC	0.702			0.682			0.437			0.430		
N	3371 Participant_ID			3371 Participant_ID			3371 Participant_ID			3371 Participant_ID		
	N/A			N/A			13 Society			13 Society		
Observations	20226			20226			20226			20226		
Marginal R^2 / Conditional R^2	0.010 / 0.705			0.101 / 0.714			0.001 / 0.438			0.022 / 0.442		

Note. fs = factor score, SPR = self-promotion and retaliation, DFR = defence of family reputation, Cooperation deviation type = the type of deviations in an individual's own cooperation from expectations of other's cooperation, SL = step-level public goods game, CG = contest game, *std. Beta* = standardized regression coefficient. The reference level for cooperation deviation type was [underinvested cooperation], for partner gender was [female], for participant gender was [female], for game order was [CG-SL]. Cooperation deviation type was controlled in the model. Societal-level honour (fs) was the societal-level factor scores of perceived normative honour values across both the SPR and DFR facets of honour, while personal honour (fs) and normative honour (fs) were the individual-level factor scores. Society was not entered to the models predicting the likelihood of efficient competition as a random intercept due to statistically insignificant variance at the between-society level.

3.5 Honour and behavioural deviations from expectations

In this section, we present the results from additional exploratory analyses examining the difference between individuals' own investments in competition and cooperation and their expectations of others' investments in these behaviours.

3.5.1 Honour and efficient/less-efficient competition

In the contest game, we categorized a given game round as *underinvested competition* if the deviation of an individual's own investment in competition from expected competition of the opponent was negative (meaning that they were willing to lose their money), as *tie* if the deviation was equal to zero MU, as *efficient competition* if the deviation was equal to one MU (because an individual could potentially win the contest game with minimal investment, thereby retaining the most remaining resources), and as *less-efficient competition* of the deviation was higher than one MU (because any positive deviations greater than one might ensure a win but reduced the individual's overall payoff in that round).

We present the results from models exploring the association between societal-level honour or individual-level honour values and the occurrence of efficient competition or less-efficient competition (i.e., the likelihood of a game round being categorized as efficient competition and less-efficient competition). Society was removed from the models testing efficient competition as a random intercept due to statistically insignificant variance at the between-society level. We did not find consistent support for the association between societal-level honour and the occurrence of either efficient or less-efficient competition using observed scores (see Table S31) and factor scores (see Table S32) of honour values. However, at the individual-level, perceiving honour values of self-promotion and retaliation as more prevalent in one's society was consistently positively associated with the occurrence of efficient competition (see Model S31b and S32b), but not with less-efficient competition (see Model S31d and S32d).

Table S31. Generalized linear mixed models with personal and perceived normative honour values (observed scores) predicting the likelihood of a game round being categorized as efficient or less-efficient competition.

	**Model S31a: Efficient competition**			**Model S31b: Efficient competition**			**Model S31c: Less-efficient competition**			**Model S31d: Less-efficient competition**		
Predictors	*Odds Ratios*	*95% CI*	*p*	*Odds Ratios*	*95% CI*	*p*	*Odds Ratios*	*95% CI*	*p*	*Odds Ratios*	*95% CI*	*p*
(Intercept)	0.110	0.102 – 0.119	<0.001	0.127	0.112 – 0.144	<0.001	0.370	0.346 – 0.395	<0.001	0.446	0.375 – 0.531	<0.001
Societal-level honour	**0.934**	**0.875 – 0.997**	**0.039**	0.934	0.876 – 0.997	0.040	**1.156**	**1.084 – 1.234**	**<0.001**	1.067	0.934 – 1.218	0.341
Normative honour (SPR)				**1.112**	**1.024 – 1.207**	**0.012**				**0.967**	**0.879 – 1.064**	**0.497**
Normative honour (DFR)				**1.005**	**0.917 – 1.101**	**0.918**				**0.947**	**0.852 – 1.053**	**0.314**
Personal honour (SPR)				**0.905**	**0.837 – 0.980**	**0.013**				**0.983**	**0.897 – 1.078**	**0.722**
Personal honour (DFR)				**0.928**	**0.850 – 1.013**	**0.093**				**1.026**	**0.925 – 1.138**	**0.632**
Competition behaviour				1.010	0.956 – 1.066	0.725				4.820	4.483 – 5.181	<0.001
Partner gender [male]				1.024	0.923 – 1.136	0.658				0.686	0.624 – 0.754	<0.001
Partner gender [unknown]				1.086	0.980 – 1.204	0.115				0.878	0.799 – 0.964	0.006
Participant gender [male]				0.935	0.825 – 1.059	0.287				0.989	0.855 – 1.144	0.882
Age				1.023	0.960 – 1.090	0.483				1.033	0.959 – 1.113	0.389
Game order [SL-CG]				0.747	0.660 – 0.846	<0.001				0.605	0.523 – 0.700	<0.001
**Random Effects**												
σ^2	3.290			3.290			3.290			3.290		
τ_{00}	1.543 $_{\text{Participant\_ID}}$			1.516 $_{\text{Participant\_ID}}$			2.180 $_{\text{Participant\_ID:Society}}$			3.006 $_{\text{Participant\_ID:Society}}$		
	N/A			N/A			0.000 $_{\text{Society}}$			0.038 $_{\text{Society}}$		
ICC	0.319			0.315			0.399			0.481		
N	3371 $_{\text{Participant\_ID}}$			3371 $_{\text{Participant\_ID}}$			3371 $_{\text{Participant\_ID}}$			3371 $_{\text{Participant\_ID}}$		
	N/A			N/A			13 $_{\text{Society}}$			13 $_{\text{Society}}$		
Observations	20226			20226			20226			20226		
Marginal R^2 / Conditional R^2	0.001 / 0.320			0.009 / 0.322			0.004 / 0.401			0.285 / 0.628		

Note. SPR = self-promotion and retaliation, DFR = defence of family reputation, SL = step-level public goods game, CG = contest game, *std. Beta* = standardized regression coefficient. The reference level for partner gender was [female], for participant gender was [female], for game order was [CG-SL]. Individuals' own level of competition was controlled in the model. Societal-level honour was the societal mean of perceived normative honour values across both the SPR and DFR facets of honour, while personal honour and normative honour (centred within society) were the individual-level unweighted means across the scale items. Society was not entered to the models predicting the likelihood of efficient competition as a random intercept due to statistically insignificant variance at the between-society level.

Table S32. Generalized linear mixed models with personal and perceived normative honour values (factor scores) predicting the likelihood of a game round being categorized as efficient or less-efficient competition.

	**Model S32a: Efficient competition**			**Model S32b: Efficient competition**			**Model S32c: Less-efficient competition**			**Model S32d: Less-efficient competition**		
Predictors	*Odds Ratios*	*95% CI*	*p*	*Odds Ratios*	*95% CI*	*p*	*Odds Ratios*	*95% CI*	*p*	*Odds Ratios*	*95% CI*	*p*
(Intercept)	0.110	0.102 – 0.119	<0.001	0.127	0.112 – 0.145	<0.001	0.373	0.338 – 0.411	<0.001	0.446	0.374 – 0.531	<0.001
Societal-level honour (fs)	**0.872**	**0.738 – 1.031**	**0.109**	0.873	0.739 – 1.031	0.110	**1.152**	**0.890 – 1.490**	**0.283**	0.903	0.636 – 1.281	0.568
Normative honour (SPR) (fs)				**1.289**	**1.111 – 1.496**	**0.001**				**0.939**	**0.791 – 1.115**	**0.475**
Normative honour (DFR) (fs)				**1.002**	**0.886 – 1.134**	**0.972**				**0.932**	**0.808 – 1.075**	**0.333**
Personal honour (SPR) (fs)				**0.897**	**0.769 – 1.047**	**0.168**				**0.918**	**0.767 – 1.099**	**0.353**
Personal honour (DFR) (fs)				**0.960**	**0.864 – 1.067**	**0.447**				**1.035**	**0.914 – 1.172**	**0.588**
Competition behaviour				1.003	0.949 – 1.059	0.928				4.832	4.495 – 5.194	<0.001
Partner gender [male]				1.024	0.923 – 1.136	0.654				0.686	0.624 – 0.754	<0.001
Partner gender [unknown]				1.086	0.980 – 1.204	0.115				0.877	0.799 – 0.964	0.006
Participant gender [male]				0.926	0.817 – 1.048	0.224				0.992	0.857 – 1.148	0.913
Age				1.011	0.949 – 1.077	0.727				1.034	0.960 – 1.114	0.378
Game order [SL-CG]				0.750	0.662 – 0.850	<0.001				0.605	0.523 – 0.700	<0.001
**Random Effects**												
σ^2	3.290			3.290			3.290			3.290		
τ_{00}	1.547 $_{\text{Participant\_ID}}$			1.517 $_{\text{Participant\_ID}}$			2.144 $_{\text{Participant\_ID:Society}}$			3.003 $_{\text{Participant\_ID:Society}}$		
	N/A			N/A			0.019 $_{\text{Society}}$			0.040 $_{\text{Society}}$		
ICC	0.320			0.316			0.397			0.481		
N	3371 $_{\text{Participant\_ID}}$			3371 $_{\text{Participant\_ID}}$			3371 $_{\text{Participant\_ID}}$			3371 $_{\text{Participant\_ID}}$		
	N/A			N/A			13 $_{\text{Society}}$			13 $_{\text{Society}}$		
Observations	20226			20226			20226			20226		
Marginal R^2 / Conditional R^2	0.001 / 0.320			0.010 / 0.322			0.001 / 0.397			0.283 / 0.628		

Note. fs = factor score, SPR = self-promotion and retaliation, DFR = defence of family reputation, SL = step-level public goods game, CG = contest game, *std. Beta* = standardized regression coefficient. The reference level for partner gender was [female], for participant gender was [female], for game order was [CG-SL]. Individuals' own level of competition was controlled in the model. Societal-level honour (fs) was the societal-level factor scores of perceived normative honour values across both the SPR and DFR facets of honour, while personal honour (fs) and normative honour (fs) were the individual-level factor scores. Society was not entered to the models predicting the likelihood of efficient competition as a random intercept due to statistically insignificant variance at the between-society level.

3.5.2 Honour and conditional/unconditional cooperation

In the step-level public goods game, we categorized a given game round as *underinvested cooperation* if the deviation of an individual's own investment in cooperation from expected cooperation of the game partner was negative (meaning that they were willing to contribute less than their partner), as *conditional cooperation* if the deviation was zero MU (because an individual was willing to match their partner's level of cooperation in that round), and as *unconditional cooperation* if the deviation was positive (because an individual was willing to contribute more than their partner, rather than conditioning their contributions on their partner's level of cooperation).

We present the results from models exploring the association between societal-level honour or individual-level honour values and the occurrence of conditional cooperation or unconditional cooperation (i.e., the likelihood of a game round being categorized as conditional cooperation or unconditional cooperation). No association was found between societal-level honour and the occurrence of either conditional or unconditional cooperation using observed scores (see Table S33) and factor scores (see Table S34) of honour values. However, at the individual-level, perceiving honour values of defence of family reputation as more prevalent in one's society was consistently positively associated with the occurrence of conditional cooperation (see Model S33b and S34b), but negatively associated with unconditional cooperation (see Model S33d and S34d).

Table S33. Generalized linear mixed models with personal and perceived normative honour values (observed scores) predicting the likelihood of a game round being categorized as conditional or unconditional cooperation.

	**Model S33a: Conditional cooperation**			**Model S33b: Conditional cooperation**			**Model S33c: Unconditional cooperation**			**Model S33d: Unconditional cooperation**		
Predictors	*Odds Ratios*	*95% CI*	*p*	*Odds Ratios*	*95% CI*	*p*	*Odds Ratios*	*95% CI*	*p*	*Odds Ratios*	*95% CI*	*p*
(Intercept)	0.407	0.369 – 0.449	<0.001	0.327	0.285 – 0.375	<0.001	0.498	0.457 – 0.544	<0.001	0.503	0.420 – 0.602	<0.001
Societal-level honour	**0.905**	**0.818 – 1.001**	**0.051**	0.896	0.811 – 0.989	0.029	**1.090**	**0.996 – 1.192**	**0.061**	0.979	0.848 – 1.129	0.769
Normative honour (SPR)				**0.945**	**0.873 – 1.023**	**0.161**				**0.991**	**0.901 – 1.089**	**0.847**
Normative honour (DFR)				**1.096**	**1.003 – 1.197**	**0.043**				**0.816**	**0.734 – 0.907**	**<0.001**
Personal honour (SPR)				**0.965**	**0.894 – 1.042**	**0.367**				**1.108**	**1.011 – 1.214**	**0.028**
Personal honour (DFR)				**1.010**	**0.927 – 1.101**	**0.816**				**0.938**	**0.847 – 1.040**	**0.224**
Cooperation behaviour				1.167	1.115 – 1.221	<0.001				5.098	4.744 – 5.477	<0.001
Partner gender [male]				1.036	0.954 – 1.125	0.397				0.735	0.669 – 0.806	<0.001
Partner gender [unknown]				1.006	0.926 – 1.092	0.892				0.941	0.858 – 1.031	0.192
Participant gender [male]				1.380	1.222 – 1.558	<0.001				0.852	0.737 – 0.985	0.030
Age				1.072	1.008 – 1.140	0.028				0.992	0.921 – 1.068	0.828
Game order [SL-CG]				1.095	0.970 – 1.236	0.143				0.965	0.835 – 1.115	0.625
**Random Effects**												
σ^2	3.290			3.290			3.290			3.290		
τ_{00}	2.211 $_{Participant\_ID:Society}$			2.108 $_{Participant\_ID:Society}$			1.863 $_{Participant\_ID:Society}$			3.066 $_{Participant\_ID:Society}$		
	0.019 $_{Society}$			0.018 $_{Society}$			0.014 $_{Society}$			0.046 $_{Society}$		
ICC	0.404			0.393			0.363			0.486		
N	3371 $_{Participant\_ID}$			3371 $_{Participant\_ID}$			3371 $_{Participant\_ID}$			3371 $_{Participant\_ID}$		
	13 $_{Society}$			13 $_{Society}$			13 $_{Society}$			13 $_{Society}$		
Observations	20226			20226			20226			20226		
Marginal R^2 / Conditional R^2	0.002 / 0.405			0.014 / 0.401			0.001 / 0.364			0.294 / 0.637		

Note. SPR = self-promotion and retaliation, DFR = defence of family reputation, SL = step-level public goods game, CG = contest game, *std. Beta* = standardized regression coefficient. The reference level for partner gender was [female], for participant gender was [female], for game order was [CG-SL]. Individuals' own level of cooperation was controlled in the model. Societal-level honour was the societal mean of perceived normative honour values across both the SPR and DFR facets of honour, while personal honour and normative honour (centred within society) were the individual-level unweighted means across the scale items.

Table S34. Generalized linear mixed models with personal and perceived normative honour values (factor scores) predicting the likelihood of a game round being categorized as conditional or unconditional cooperation.

	**Model S34a: Conditional cooperation**			**Model S34b: Conditional cooperation**			**Model S34c: Unconditional cooperation**			**Model S34d: Unconditional cooperation**		
Predictors	*Odds Ratios*	*95% CI*	*p*	*Odds Ratios*	*95% CI*	*p*	*Odds Ratios*	*95% CI*	*p*	*Odds Ratios*	*95% CI*	*p*
(Intercept)	0.407	0.365 – 0.454	<0.001	0.328	0.283 – 0.380	<0.001	0.499	0.453 – 0.550	<0.001	0.501	0.421 – 0.596	<0.001
Societal-level honour (fs)	**0.935**	**0.699 – 1.252**	**0.654**	0.908	0.677 – 1.217	0.518	**1.052**	**0.813 – 1.362**	**0.700**	0.808	0.572 – 1.142	0.228
Normative honour (SPR) (fs)				**0.922**	**0.800 – 1.064**	**0.267**				**0.982**	**0.828 – 1.165**	**0.837**
Normative honour (DFR) (fs)				**1.156**	**1.026 – 1.303**	**0.017**				**0.785**	**0.681 – 0.905**	**0.001**
Personal honour (SPR) (fs)				**0.997**	**0.858 – 1.158**	**0.969**				**1.099**	**0.920 – 1.313**	**0.299**
Personal honour (DFR) (fs)				**1.041**	**0.938 – 1.155**	**0.450**				**0.906**	**0.801 – 1.024**	**0.114**
Cooperation behaviour				1.164	1.112 – 1.217	<0.001				5.085	4.733 – 5.464	<0.001
Partner gender [male]				1.036	0.954 – 1.125	0.397				0.735	0.669 – 0.806	<0.001
Partner gender [unknown]				1.006	0.926 – 1.092	0.892				0.941	0.858 – 1.031	0.193
Participant gender [male]				1.372	1.216 – 1.549	<0.001				0.856	0.741 – 0.990	0.036
Age				1.065	1.001 – 1.132	0.046				0.993	0.923 – 1.069	0.854
Game order [SL-CG]				1.095	0.970 – 1.236	0.141				0.967	0.837 – 1.117	0.647
**Random Effects**												
σ^2	3.290			3.290			3.290			3.290		
τ_{00}	2.210 Participant_ID:Society			2.103 Participant_ID:Society			1.862 Participant_ID:Society			3.053 Participant_ID:Society		
	0.028 Society			0.029 Society			0.021 Society			0.039 Society		
ICC	0.405			0.393			0.364			0.485		
N	3371 Participant_ID			3371 Participant_ID			3371 Participant_ID			3371 Participant_ID		
	13 Society			13 Society			13 Society			13 Society		
Observations	20226			20226			20226			20226		
Marginal R^2 / Conditional R^2	0.000 / 0.405			0.014 / 0.401			0.000 / 0.364			0.295 / 0.637		

Note. fs = factor score, SPR = self-promotion and retaliation, DFR = defence of family reputation, SL = step-level public goods game, CG = contest game, *std. Beta* = standardized regression coefficient. The reference level for partner gender was [female], for participant gender was [female], for game order was [CG-SL]. Individuals' own level of cooperation was controlled in the model. Societal-level honour (fs) was the societal-level factor scores of perceived normative honour values across both the SPR and DFR facets of honour, while personal honour (fs) and normative honour (fs) were the individual-level factor scores.

4. Descriptives

Table S35. Sample descriptives for each society.

		N		Age		SSS (1-10)	Education (1-8)	Ethnicity	
**Societies**	**ISO-3**	*n*	% Female	*M (SD)*	Range	*M (SD)*	*M (SD)*	Majority	% Majority
Egypt	EGY	270	50.38%	40.78 (14.00)	[18, 68]	6.84 (2.04)	5.23 (1.34)	Egyptian	99.63%
Greece	GRC	255	49.61%	40.59 (13.76)	[18, 69]	5.40 (1.55)	4.60 (1.25)	Greek	98.43%
Greek Cypriot community	CYP-S*	269	50.93%	41.22 (14.20)	[18, 77]	5.91 (1.54)	4.21 (1.42)	Greek Cypriot	97.76%
Italy	ITA	270	50.37%	41.14 (14.21)	[19, 79]	5.61 (1.51)	4.32 (1.48)	White: Italian	98.52%
Japan	JPN	261	49.23%	41.56 (14.91)	[18, 78]	4.76 (2.04)	4.83 (1.11)	Asian	98.05%
Lebanon	LBN	250	53.01%	39.25 (12.83)	[18, 63]	5.54 (1.67)	4.32 (1.69)	Lebanese	98.76%
Morocco	MAR	260	49.22%	39.81 (13.15)	[18, 68]	5.33 (1.71)	3.30 (1.85)	Moroccan	99.23%
South Korea	KOR	271	49.82%	41.21 (14.61)	[18, 79]	4.94 (1.95)	4.56 (1.37)	Korean	99.63%
Spain	ESP	249	48.19%	40.81 (14.30)	[18, 77]	5.72 (1.52)	4.27 (1.63)	Spanish	96.79%
Turkish Cypriot community	CYP-N*	245	49.80%	40.32 (14.46)	[18, 77]	5.89 (2.09)	3.47 (1.63)	Turkish Cypriot	82.50%
Türkiye	TUR	260	50.77%	40.72 (14.01)	[18, 79]	5.80 (1.91)	3.58 (1.56)	Turkish	90.63%
United Kingdom	GBR	255	49.80%	41.47 (15.79)	[18, 80]	5.07 (1.97)	4.58 (1.45)	White: English / Welsh / Scottish / Northern Irish	88.19%
United States	USA	256	51.01%	41.33 (16.25)	[18, 80]	5.79 (2.37)	4.98 (1.36)	Caucasian American/White	67.19%
	**Total**	**3,371**	**50.16%**	**40.79 (14.36)**	**[18, 80]**	**5.59 (1.92)**	**4.33 (1.58)**	/	**93.60%**

Table S35 (continued)

		Living Environment			Comprehension check of games		
**Societies**	**ISO-3**	% Urban	**Language**	**Panel**	% Failed 1	% Failed 2	% Failed 3
Egypt	EGY	92.22%	Arabic	Tln's partner	24.44%	28.89%	19.26%
Greece	GRC	94.12%	Greek	Toluna	23.92%	28.24%	10.20%
Greek Cypriot community	CYP-S*	88.10%	Greek	CYMAR	18.22%	22.68%	8.18%
Italy	ITA	83.33%	Italian	Toluna	25.19%	24.81%	16.67%
Japan	JPN	75.86%	Japanese	Toluna	22.61%	23.37%	14.94%
Lebanon	LBN	78.40%	Arabic	Tln's partner	26.40%	20.80%	8.40%
Morocco	MAR	95.00%	Arabic	Tln's partner	27.69%	20.38%	10.77%
South Korea	KOR	98.15%	Korean	Toluna	25.09%	18.82%	16.97%
Spain	ESP	88.35%	Spanish	Toluna	24.10%	27.71%	16.87%
Turkish Cypriot community	CYP-N*	74.29%	Turkish	Statika	32.24%	29.39%	16.73%
Türkiye	TUR	95.00%	Turkish	Tln's partner	33.08%	27.31%	21.15%
United Kingdom	GBR	78.82%	English	Toluna	29.80%	30.20%	14.51%
United States	USA	71.88%	English	Toluna	28.91%	32.81%	21.09%
	**Total**	**85.79%**	/	/	**26.22%**	**25.75%**	**15.07%**

Note. ISO-3 = three-letter country codes published by the International Organization for Standardization (ISO) to represent countries, dependent territories, and special areas of geographical interest. *To differentiate between the Turkish Cypriot and Greek Cypriot communities, we applied CYP-N for the Turkish Cypriot community, and CYP-S for the Greek Cypriot community. SSS = subjective social status, Education = the average of the father's and mother's education levels, Living Envir. = living environment, % Female = percentage of female participants, % Majority = percentage of participants who self-identified as belonging to the major ethnic group in the respective society, % Urban = percentage of participants with experience living in urban environments (incl. those who have lived in both urban and rural environments), Tln's partner = Toluna's third party panel provider, % Failed 1 = the percentage of participants who answered exactly one out of four comprehension questions incorrectly after two attempts, % Failed 2 = the percentage of participants who answered exactly two out of four comprehension questions incorrectly after two attempts, % Failed 3 = the percentage of participants who answered exactly three out of four comprehension questions incorrectly after two attempts. We found significant differences across societies in participants' failure to answer the comprehension questions of economic games correctly. The between-society variance was significantly different from zero for the percentage of participants who failed exactly one question, $\chi 2$ (1) = 4.33, p = .037; two questions, $\chi 2$ (1) = 6.34, p = .012; and three questions, $\chi 2$ (1) = 19.10, p < .001.

Table S36. Societal means of competition, cooperation and expectations of these behaviours from others.

Societies	Competition		Comp. Expectation		Cooperation		Coop. Expectation	
	M	*SD*	*M*	*SD*	*M*	*SD*	*M*	*SD*
Egypt	6.95	1.97	6.02	1.78	6.65	1.97	6.22	1.72
Greece	6.49	1.98	5.73	1.81	6.42	1.65	6.04	1.66
Greek Cypriot community	6.57	2.13	5.95	1.85	6.41	1.80	6.29	1.50
Italy	6.23	1.91	5.74	1.72	6.26	1.59	6.07	1.56
Japan	6.41	2.32	5.71	2.09	5.71	2.06	5.64	1.83
Lebanon	6.12	1.84	5.04	1.93	5.97	1.61	5.68	1.83
Morocco	6.77	1.94	5.93	1.80	6.37	1.67	5.96	1.48
South Korea	6.20	1.96	5.55	1.76	6.01	1.79	6.01	1.57
Spain	6.28	2.08	5.47	1.77	6.16	1.78	5.82	1.64
Turkish Cypriot community	5.94	1.89	5.76	1.86	5.96	1.76	5.96	1.76
Türkiye	6.76	2.04	6.18	1.82	6.67	1.89	6.44	1.60
United Kingdom	6.25	1.88	5.57	1.75	6.09	1.69	5.61	1.60
United States	6.22	2.13	5.57	1.94	6.14	2.07	5.78	1.89
**Total**	**6.40**	**2.03**	**5.71**	**1.86**	**6.22**	**1.82**	**5.97**	**1.68**

Note. Comp. Expectation = expectation of others' competition, Coop. Expectation = expectation of others' cooperation, *M* = societal means, *SD* = standard deviations of societal means.

Table S37. Societal mean percentages of rounds involving coordinative decisions and expectations (contributing or expecting others to contribute 8 or 6 MUs), and percentages of rounds categorized as different types of anticipated coordination success.

Societies	Coordinative decisions and expectations				Anticipated coordination success		
	Coop. 8	Expe. 8	Coop. 6	Expe. 6	Efficient	Less efficient	Failed
Egypt	17%	17%	18%	23%	27%	35%	37%
Greece	19%	18%	21%	26%	23%	40%	38%
Greek Cypriot community	23%	23%	19%	25%	29%	39%	33%
Italy	19%	18%	24%	27%	19%	44%	36%
Japan	13%	12%	22%	30%	16%	40%	44%
Lebanon	14%	14%	21%	21%	16%	37%	46%
Morocco	20%	18%	24%	27%	22%	40%	38%
South Korea	16%	17%	23%	26%	18%	40%	42%
Spain	17%	17%	24%	26%	19%	41%	40%
Turkish Cypriot community	12%	14%	18%	19%	18%	37%	45%
Türkiye	18%	18%	18%	24%	30%	35%	35%
United Kingdom	17%	13%	20%	23%	16%	37%	46%
United States	15%	14%	18%	20%	22%	31%	47%
**Total**	**17%**	**17%**	**21%**	**24%**	**21%**	**38%**	**41%**

Note. Coop. 8 = contributing exactly 8 MUs in the step-level public goods game, Expe. 8 = expecting the game partner to contribute exactly 8 MUs, Coop. 6 = contributing exactly 6 MUs, Expe. 6 = expecting the game partner to contribute exactly 6 MUs, Efficient = anticipated success of efficient coordination (expected sum contributions reached 16 MUs), Less-efficient = anticipated success of less efficient coordination (expected sum contributions reached 12 MUs but not 16 MUs), Failed = anticipated failed coordination (expected sum contributions did not reach 12 MUs).

Table S38. Societal mean percentages of rounds categorized as different types of competition deviating from expectations of other's competition and cooperation deviating from expectations of other's cooperation.

Societies	Competition deviated from expectation				Cooperation deviated from expectation		
	Underinvested competition	Tie	Efficient competition	Less efficient competition	Underinvested cooperation	Conditional cooperation	Unconditional cooperation
Egypt	20%	29%	12%	40%	28%	30%	42%
Greece	19%	33%	16%	32%	24%	37%	39%
Greek Cypriot community	21%	33%	17%	29%	28%	38%	34%
Italy	24%	29%	17%	30%	28%	36%	36%
Japan	19%	33%	18%	30%	26%	43%	31%
Lebanon	17%	25%	19%	39%	30%	29%	41%
Morocco	20%	30%	12%	38%	28%	31%	42%
South Korea	22%	27%	18%	33%	31%	35%	34%
Spain	21%	26%	18%	36%	26%	35%	38%
Turkish Cypriot community	30%	30%	12%	28%	32%	30%	38%
Türkiye	23%	34%	11%	31%	30%	35%	36%
United Kingdom	22%	30%	16%	32%	27%	31%	42%
United States	22%	34%	11%	32%	28%	34%	38%
**Total**	**21%**	**30%**	**15%**	**33%**	**28%**	**34%**	**38%**

Note. Underinvested competition = individual's own competition was less than expected competition from the other in a given round, Tie = the individual competed exactly the same level as the expected level from the other in a given round, Efficient competition = the individual's own competition was just one MU more than the expected competition from the other in a given round, Less-efficient competition = the individual's own competition was at least two MUs more than the expected competition from the other in a given round, Underinvested cooperation = the individual's own contribution was less than expected contribution from the other in a given round, Conditional cooperation = the individual contributed exactly the same level as the expected level from the other in a given round, Unconditional cooperation = the individual's own contribution was more than the expected contribution from the other in a given round.

Table S39. Societal means and factor scores of perceived normative and personal honour values.

	**Perceived normative honour values**				**Personal honour values**				**Beliefs in a zero-sum game**				**Relational mobility**			
**Societies**	*M*	*SD*	FS	ω_t	*M*	*SD*	FS	ω_t	*M*	*SD*	FS	ω_t	*M*	*SD*	FS	ω_t
Egypt	6.03	0.80	0.41	0.87	6.03	0.79	0.55	0.86	4.15	1.21	-0.21	0.92	3.76	0.54	-0.06	0.90
Greece	5.29	0.99	0.19	0.90	4.89	0.96	-0.10	0.88	3.96	1.00	-0.05	0.91	3.90	0.64	0.22	0.77
Greek Cypriot community	5.35	0.97	0.48	0.86	4.74	1.07	-0.05	0.87	3.59	0.87	-0.32	0.86	3.88	0.64	0.23	0.84
Italy	5.04	0.94	-0.09	0.89	4.73	1.01	-0.28	0.90	3.81	0.97	-0.07	0.92	3.78	0.54	-0.02	0.90
Japan	4.50	0.88	-0.34	0.89	4.49	0.98	-0.34	0.91	3.64	0.87	0.13	0.91	3.51	0.47	-0.35	0.89
Lebanon	5.64	0.83	-0.08	0.88	5.57	0.82	-0.08	0.89	4.30	0.95	0.03	0.90	3.62	0.39	-0.24	0.86
Morocco	5.66	1.04	0.55	0.91	5.67	0.91	0.81	0.86	3.82	1.11	-0.29	0.90	3.84	0.55	0.09	0.86
South Korea	4.89	0.92	0.05	0.89	4.77	0.93	0.03	0.89	3.97	0.95	0.32	0.93	3.68	0.52	-0.13	0.91
Spain	4.98	1.12	-0.16	0.91	4.99	1.13	-0.05	0.92	3.97	1.03	0.17	0.91	3.81	0.60	0.10	0.81
Turkish Cypriot community	5.05	0.86	0.17	0.82	4.92	0.95	0.25	0.85	3.81	0.97	0.11	0.85	3.79	0.58	0.05	0.76
Türkiye	5.50	1.00	0.15	0.92	5.51	0.95	0.30	0.90	4.28	1.01	0.18	0.88	3.73	0.49	0.00	0.90
United Kingdom	4.45	1.10	-0.60	0.89	4.43	1.06	-0.55	0.89	/	/	/	/	3.79	0.53	0.02	0.85
United States	4.44	1.40	-0.72	0.92	4.61	1.33	-0.49	0.93	/	/	/	/	3.86	0.63	0.11	0.91
**Total**	**5.14**	**1.11**	**/**	**0.90**	**5.03**	**1.11**	**/**	**0.90**	**3.93**	**1.02**	**/**	**0.89**	**3.76**	**0.56**	**/**	**0.88**

Note. *M* = societal means, *SD* = standard deviations of societal means, FS = factor scores at the between-society level obtained from the multilevel confirmatory factor analysis, adjusting for response style (see Section 2 for more information). ω_t (Omega Total) represented the overall reliability of the scale, accounting for both general and specific factors, indicating the proportion of variance in the scale scores that can be attributed to consistent sources rather than random error[15]. Values above 0.80 are generally considered to indicate good reliability. Beliefs in a zero-sum game were not reported for the U.K. and the U.S.A. because participants' responses were given on a seven-point scale in these two societies due to a survey programming error, while a six-point scale was used in the other sample.

Table S40. Societal means of each facet of perceived normative and personal honour values.

	**Perceived normative honour values**				**Personal honour values**			
	**SPR**		**DFR**		**SPR**		**DFR**	
**Society**	*M*	*SD*	*M*	*SD*	*M*	*SD*	*M*	*SD*
Egypt	5.57	1.09	6.33	0.88	5.40	1.21	6.44	0.82
Greece	4.94	1.21	5.53	1.10	4.19	1.24	5.36	1.13
Greek Cypriot community	4.82	1.14	5.70	1.12	3.90	1.30	5.31	1.21
Italy	4.68	1.17	5.28	1.04	4.03	1.31	5.20	1.14
Japan	4.26	0.98	4.67	0.99	4.21	1.03	4.67	1.09
Lebanon	5.45	0.99	5.77	0.91	5.31	1.04	5.75	0.91
Morocco	5.01	1.25	6.10	1.20	4.82	1.31	6.24	0.97
South Korea	4.53	1.12	5.12	1.03	4.28	1.11	5.10	1.04
Spain	4.76	1.15	5.12	1.24	4.60	1.21	5.24	1.27
Turkish Cypriot community	4.67	1.07	5.30	0.99	4.32	1.28	5.31	1.04
Türkiye	5.13	1.11	5.75	1.12	4.92	1.13	5.90	1.05
United Kingdom	3.99	1.24	4.75	1.25	3.88	1.33	4.80	1.19
United States	3.95	1.60	4.76	1.56	4.02	1.64	5.01	1.47
**Total**	**4.75**	**1.26**	**5.40**	**1.23**	**4.45**	**1.34**	**5.41**	**1.22**

Note. SPR = self-promotion and retaliation, DFR = defence of family reputation, *M* = societal means, *SD* = standard deviations of societal means.

In Figures S4 to S5 below, we reported the associations between predictor variables at the between-society and within-society levels, using observed scores and factor scores (see Section 2 for more information about obtaining factor scores for multi-item measures). At the between-society level, societal-level honour was not associated with societal-level relational mobility; societal-level honour was negatively associated with societal-level beliefs in a zero-sum game when using factor scores, but not when using observed scores (observed scores: $r = .186$, see Figure S4a; factor scores: $r = -.715$, see Figure S4b). However, the results from multilevel confirmatory factor analyses showed nonsignificant variance for beliefs in a zero-sum game (see Section 2.2) and relational mobility (see Section 2.3) at the between-society level. This indicated that these two variables may not vary significantly at the societal level among the current samples. Interpretations of these societal-level correlation coefficients in Figures S4a and S4b should also be made cautiously, given the small number of societies.

Based on both observed scores and factor scores of societal-level honour, we report associations between this variable and other societal-level indicators that were retrieved from online databases (see Table S13 for the operationalization of these indicators). We observed substantial negative correlations between societal-level honour and market competitiveness (observed score: $r = -.971$, see Figure S4a; factor score: $r = -.769$, see Figure S4b), as well as with GNI (observed score: $r = -.909$, see Figure S4a; factor score: $r = -.790$, see Figure S4b), GDP per capita (observed score: $r = -.864$, see Figure S4a; factor score: $r = -.764$, see Figure S4b), and corruption perception index (observed score: $r = -.947$, see Figure S4a; factor score: $r = -.668$, see Figure S4b). Conversely, a large positive correlation was found between societal-level honour and historical prevalence of infectious disease (observed score: $r = .668$, see Figure S4a; factor score: $r = .637$, see Figure S4b).

At the within-society level, self-promotion and retaliation and defence of family reputation were positively correlated when honour was measured as personal values

(observed scores: $r = .457$, see Figure S5a; factor scores: $r = .368$, see Figure S5b), and the strength of the positive association became stronger when honour was measured as perceived normative values (observed scores: $r = .516$; factor scores: $r = .671$). Relational mobility was consistently positively associated with the defence of family reputation facet of personal values (observed scores: $r = .148$; factor scores: $r = .316$), and perceived normative values (observed scores: $r = .203$; factor scores: $r = .347$). Beliefs in a zero-sum game was positively associated with only the self-promotion and retaliation dimension of honour values, regardless of whether these values were personally endorsed (observed scores: $r = .369$; factor scores: $r = .163$) or perceived as societal norms (observed scores: $r = .264$; factor scores: $r = .101$), but negatively associated with relational mobility (observed scores: $r = –.183$; factor scores: $r = –.068$).

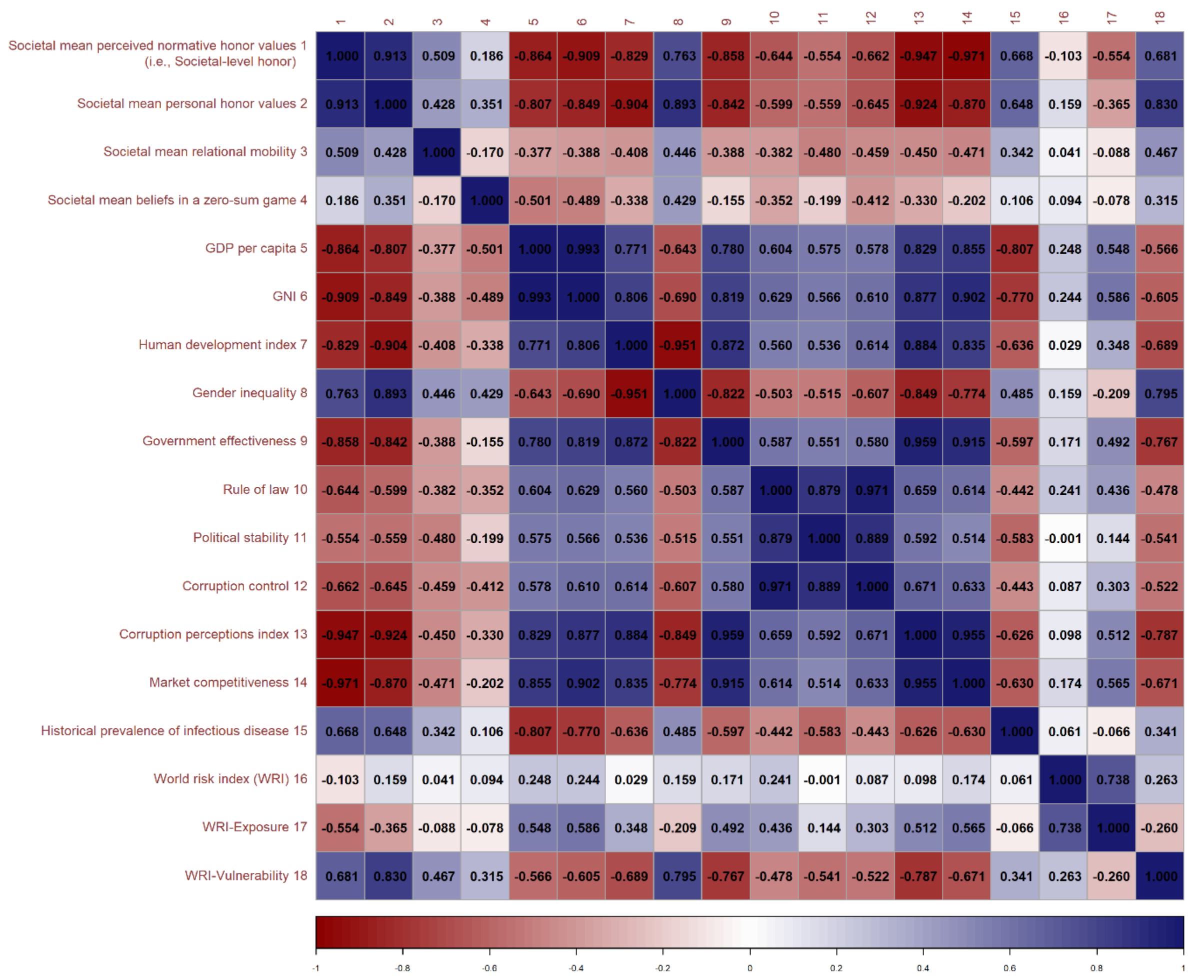

Figure S4a. Intercorrelations between societal-level indicators (using observed scores for variables 1-4, $N_{society}$ = 13).

Note. Correlation coefficients with absolute values greater than or equal to .578 were statistically significant at the 0.05 level. Beliefs in a zero-sum game ($N_{society}$ = 11) were not reported for the U.K. and the U.S.A. because participants' responses were given on a seven-point scale in these two societies due to a survey programming error, while a six-point scale was used in the other societies. Societal-level indicators from (5) GDP per capita to (18) WRI-Vulnerability were not retrievable for the Turkish Cypriot community ($N_{society}$ = 12).

	1	2	3	4	5	6	7	8	9	10	11	12	13	14	15	16	17	18
Societal-level perceived normative honor values (fs) 1	1.000	0.841	0.269	-0.715	-0.764	-0.790	-0.569	0.445	-0.507	-0.491	-0.379	-0.507	-0.668	-0.769	0.637	-0.224	-0.517	0.244
Societal-level personal honor values (fs) 2	0.841	1.000	0.112	-0.381	-0.779	-0.807	-0.765	0.695	-0.558	-0.470	-0.398	-0.522	-0.726	-0.729	0.680	0.112	-0.340	0.479
Societal-level relational mobility (fs) 3	0.269	0.112	1.000	-0.454	0.184	0.101	0.136	-0.150	0.127	0.097	0.485	0.094	0.037	-0.118	-0.387	-0.182	-0.340	-0.282
Societal-level beliefs in a zero-sum game (fs) 4	-0.715	-0.381	-0.454	1.000	0.322	0.377	0.485	-0.429	0.371	-0.051	-0.173	0.061	0.392	0.587	-0.175	0.194	0.311	-0.060
GDP per capita 5	-0.764	-0.779	0.184	0.322	1.000	0.993	0.771	-0.643	0.780	0.604	0.575	0.578	0.829	0.855	-0.807	0.248	0.548	-0.566
GNI 6	-0.790	-0.807	0.101	0.377	0.993	1.000	0.806	-0.690	0.819	0.629	0.566	0.610	0.877	0.902	-0.770	0.244	0.586	-0.605
Human development index 7	-0.569	-0.765	0.136	0.485	0.771	0.806	1.000	-0.951	0.872	0.560	0.536	0.614	0.884	0.835	-0.636	0.029	0.348	-0.689
Gender inequality 8	0.445	0.695	-0.150	-0.429	-0.643	-0.690	-0.951	1.000	-0.822	-0.503	-0.515	-0.607	-0.849	-0.774	0.485	0.159	-0.209	0.795
Government effectiveness 9	-0.507	-0.558	0.127	0.371	0.780	0.819	0.872	-0.822	1.000	0.587	0.551	0.580	0.959	0.915	-0.597	0.171	0.492	-0.767
Rule of law 10	-0.491	-0.470	0.097	-0.051	0.604	0.629	0.560	-0.503	0.587	1.000	0.879	0.971	0.659	0.614	-0.442	0.241	0.436	-0.478
Political stability 11	-0.379	-0.398	0.485	-0.173	0.575	0.566	0.536	-0.515	0.551	0.879	1.000	0.889	0.592	0.514	-0.583	-0.001	0.144	-0.541
Corruption control 12	-0.507	-0.522	0.094	0.061	0.578	0.610	0.614	-0.607	0.580	0.971	0.889	1.000	0.671	0.633	-0.443	0.087	0.303	-0.522
Corruption perceptions index 13	-0.668	-0.726	0.037	0.392	0.829	0.877	0.884	-0.849	0.959	0.659	0.592	0.671	1.000	0.955	-0.626	0.098	0.512	-0.787
Market competitiveness 14	-0.769	-0.729	-0.118	0.587	0.855	0.902	0.835	-0.774	0.915	0.614	0.514	0.633	0.955	1.000	-0.630	0.174	0.565	-0.671
Historical prevalence of infectious disease 15	0.637	0.680	-0.387	-0.175	-0.807	-0.770	-0.636	0.485	-0.597	-0.442	-0.583	-0.443	-0.626	-0.630	1.000	0.061	-0.066	0.341
World risk index (WRI) 16	-0.224	0.112	-0.182	0.194	0.248	0.244	0.029	0.159	0.171	0.241	-0.001	0.087	0.098	0.174	0.061	1.000	0.738	0.263
WRI-Exposure 17	-0.517	-0.340	-0.340	0.311	0.548	0.586	0.348	-0.209	0.492	0.436	0.144	0.303	0.512	0.565	-0.066	0.738	1.000	-0.260
WRI-Vulnerability 18	0.244	0.479	-0.282	-0.060	-0.566	-0.605	-0.689	0.795	-0.767	-0.478	-0.541	-0.522	-0.787	-0.671	0.341	0.263	-0.260	1.000

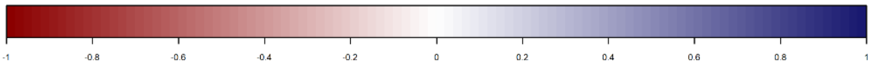

Figure S4b. Intercorrelations between societal-level indicators (using factor scores for variables 1-4, $N_{\text{society}} = 13$).

Note. Correlation coefficients with absolute values greater than or equal to .580 were statistically significant at the 0.05 level. Beliefs in a zero-sum game ($N_{\text{society}} = 11$) were not reported for the U.K. and the U.S.A. because participants' responses were given on a seven-point scale in these two societies due to a survey programming error, while a six-point scale was used in the other societies. Societal-level indicators from (5) GDP per capita to (18) WRI-Vulnerability were not retrievable for the Turkish Cypriot community ($N_{\text{society}} = 12$).

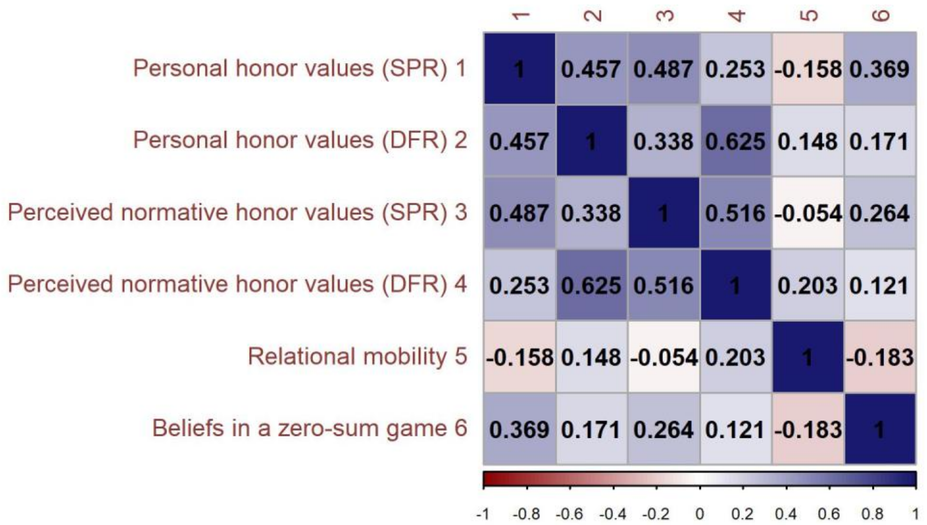

Figure S5a. Correlations between observed scores of predictor variables at the within-society level ($N_{\text{participant}}$ = 3,371).
Note. SPR = self-promotion and retaliation, DFR = defence of family reputation. All the correlation coefficients were statistically significant at the 0.05 level. Beliefs in a zero-sum game ($N_{\text{participant}}$ = 2,860) were not reported for the U.K. and the U.S.A. because participants' responses were given on a seven-point scale in these two societies due to a survey programming error, while a six-point scale was used in the other societies.

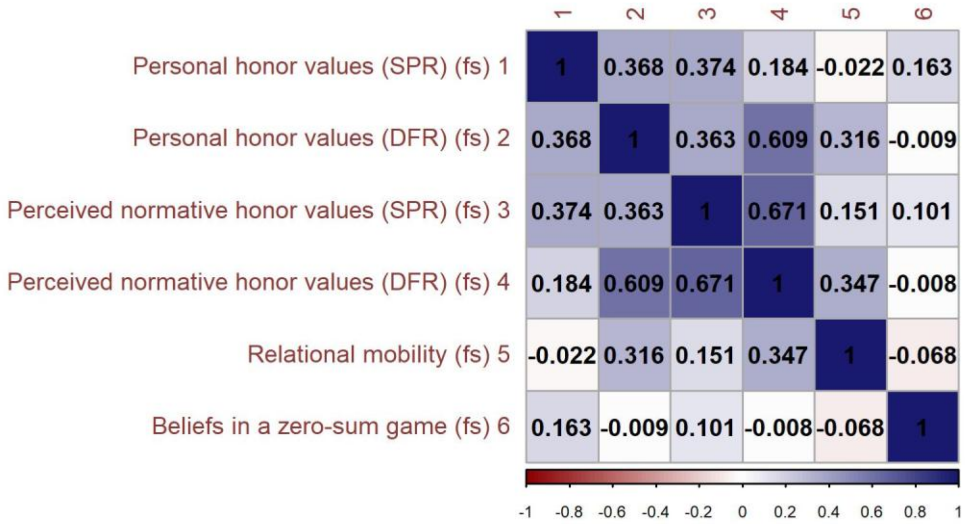

Figure S5b. Correlations between factor scores of predictor variables at the within-society level ($N_{\text{participant}}$ = 3,371).
Note. SPR = self-promotion and retaliation, DFR = defence of family reputation. Correlation coefficients with absolute values greater than or equal to .068 were statistically significant at the 0.05 level. Beliefs in a zero-sum game ($N_{\text{participant}}$ = 2,860) were not reported for the U.K. and the U.S.A. because participants' responses were given on a seven-point scale in these two societies due to a survey programming error, while a six-point scale was used in the other societies.

5. Games

5.1 Contest game

In the contest game, each player received an endowment of 10 MUs and decided how many of the 10 MUs they wanted to invest into a challenge pool (investment = x_i, $0 \leq x_i \leq 10$) or keep for themselves. Player i's payoff π_i was defined as (see Table S41 for the discrete payoff matrix):

$$\pi_i = \begin{cases} (10 - x_i) + (10 - x_j), & \text{if } x_i > x_j \text{ (i.e., } i \text{ wins)} \\ 10 - x_i, & \text{if } x_i = x_j \text{ (i.e., } i \text{ and } j \text{ tie)} \\ 0, & \text{if } x_i < x_j \text{ (i.e., } i \text{ loses).} \end{cases} \quad (1)$$

Table S41. Discrete payoff matrix for the contest game.

		Player *i*										
		0	1	2	3	4	5	6	7	8	9	10
Player *j*	0	10, 10	0, 19	0, 18	0, 17	0, 16	0, 15	0, 14	0, 13	0, 12	0, 11	0, 10
	1	19, 0	9, 9	0, 17	0, 16	0, 15	0, 14	0, 13	0, 12	0, 11	0, 10	0, 9
	2	18, 0	17, 0	8, 8	0, 15	0, 14	0, 13	0, 12	0, 11	0, 10	0, 9	0, 8
	3	17, 0	16, 0	15, 0	7, 7	0, 13	0, 12	0, 11	0, 10	0, 9	0, 8	0, 7
	4	16, 0	15, 0	14, 0	13, 0	6, 6	0, 11	0, 10	0, 9	0, 8	0, 7	0, 6
	5	15, 0	14, 0	13, 0	12, 0	11, 0	5, 5	0, 9	0, 8	0, 7	0, 6	0, 5
	6	14, 0	13, 0	12, 0	11, 0	10, 0	9, 0	4, 4	0, 7	0, 6	0, 5	0, 4
	7	13, 0	12, 0	11, 0	10, 0	9, 0	8, 0	7, 0	3, 3	0, 5	0, 4	0, 3
	8	12, 0	11, 0	10, 0	9, 0	8, 0	7, 0	6, 0	5, 0	2, 2	0, 3	0, 2
	9	11, 0	10, 0	9, 0	8, 0	7, 0	6, 0	5, 0	4, 0	3, 0	1, 1	0, 1
	10	10, 0	9, 0	8, 0	7, 0	6, 0	5, 0	4, 0	3, 0	2, 0	1, 0	0, 0

In the contest game described above, the player investing more wins, taking the opponent's remaining MUs, while a tie results in each player retaining their non-invested MUs. When neither player invests in competition ($x_i = 0, x_j = 0$), any unilateral deviation from this strategy (i.e., the deviating player investing at least 1 MU) would lead the deviating player to win, making the deviation advantageous. Thus, investing 1 MU while the opponent invests nothing yields the highest payoff for a player. However, both players always have an incentive to increase their investment by one MU more than the other player to ensure the highest possible payoff given the other player's investment. This continuous escalation leads to four stable outcomes where each player invests either 9 or 10 MUs, as any deviations from

the combination of these decisions would result in a guaranteed loss for the deviating player. Thus, this game involves four Nash equilibria where both players invest either 9 or 10 MUs.

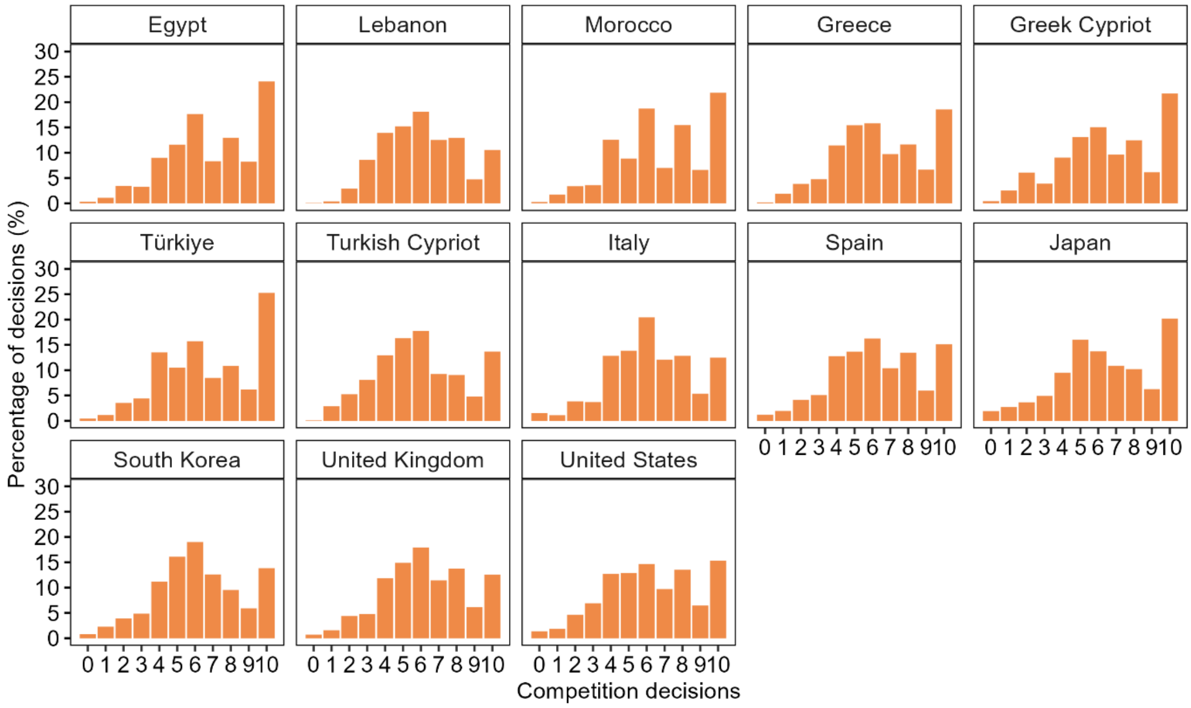

Figure S6a. Percentage of each competition decision (0-10) per society.

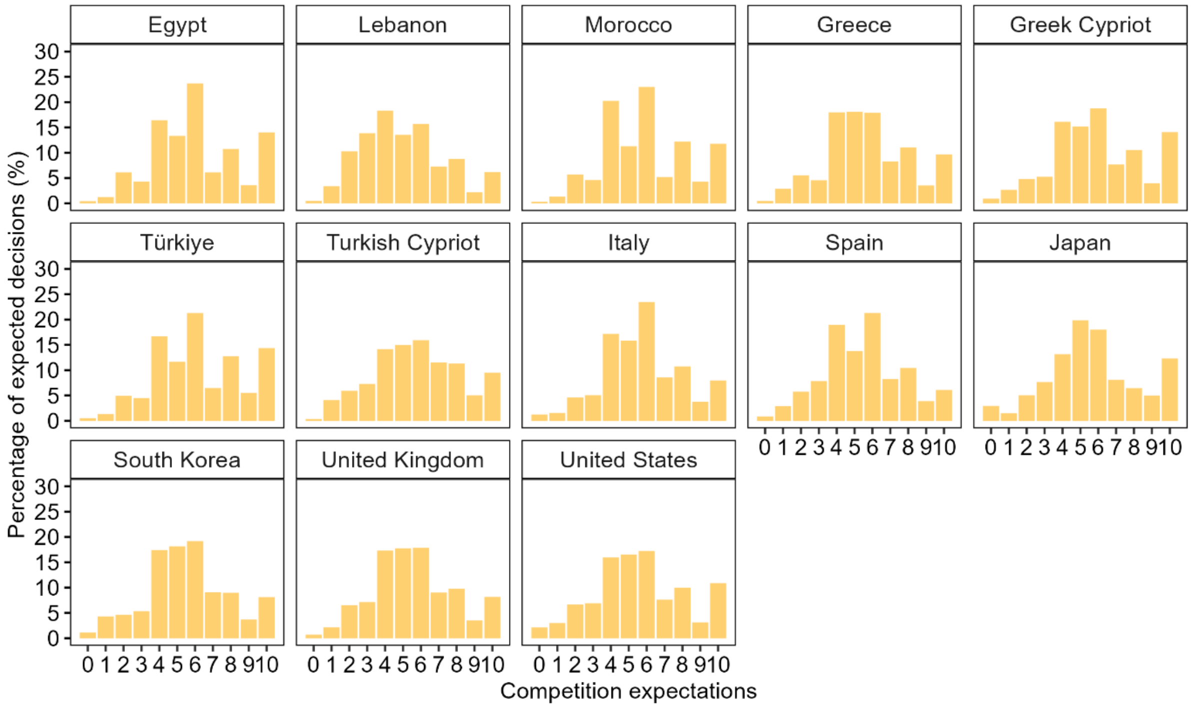

Figure S6b. Percentage of each expected cooperation decision from others (0-10) per society.

5.2 Step-level public goods game

In the step-level public goods game, each player received an endowment of 10 MUs and decided how many of the 10 MUs they wanted to invest into a common pool (investment = x_i, $0 \leq x_i \leq 10$) or keep for themselves. Player i's payoff π_i was defined as (see Table S42 for the discrete payoff matrix):

$$\pi_i = \begin{cases} 10 - x_i, & \text{if} \quad x_i + x_j < 12 \\ 10 - x_i + 10, & \text{if} \quad 12 \leq x_i + x_j < 16 \\ 10 - x_i + 15, & \text{if} \quad 16 \leq x_i + x_j. \end{cases} \tag{2}$$

Table S42. Discrete payoff matrix for the step-level public goods game.

		Player i										
		0	1	2	3	4	5	6	7	8	9	10
Player j	0	10, 10	10, 9	10, 8	10, 7	10, 6	10, 5	10, 4	10, 3	10, 2	10, 1	10, 0
	1	9, 10	9, 9	9, 8	9, 7	9, 6	9, 5	9, 4	9, 3	9, 2	9, 1	9, 0
	2	8, 10	8, 9	8, 8	8, 7	8, 6	8, 5	8, 4	8, 3	8, 2	8, 1	18, 10
	3	7, 10	7, 9	7, 8	7, 7	7, 6	7, 5	7, 4	7, 3	7, 2	17, 11	17, 10
	4	6, 10	6, 9	6, 8	6, 7	6, 6	6, 5	6, 4	6, 3	16, 12	16, 11	16, 10
	5	5, 10	5, 9	5, 8	5, 7	5, 6	5, 5	5, 4	15, 13	15, 12	15, 11	15, 10
	6	4, 10	4, 9	4, 8	4, 7	4, 6	4, 5	14, 14	14, 13	14, 12	14, 11	19, 15
	7	3, 10	3, 9	3, 8	3, 7	3, 6	13, 15	13, 14	13, 13	13, 12	18, 16	18, 15
	8	2, 10	2, 9	2, 8	2, 7	12, 16	12, 15	12, 14	12, 13	17, 17	17, 16	17, 15
	9	1, 10	1, 9	1, 8	11, 17	11, 16	11, 15	11, 14	16, 18	16, 17	16, 16	16, 15
	10	0, 10	0, 9	10, 18	10, 17	10, 16	10, 15	15, 19	15, 18	15, 17	15, 16	15, 15

In the step-level public goods game described above, there are two provision points (i.e., at 12 and 16 MUs), and two robust pure strategy Nash equilibria. The first equilibrium is where neither player contributes to the common pool ($x_i = 0, x_j = 0$), resulting in each player retaining their initial endowment of 10 MUs. The second equilibrium is when both players contribute 8 MUs ($x_i = 8, x_j = 8$), meeting the second provision point and resulting in a payoff of 17 MUs each. These equilibria existed because, in each scenario, players have no incentive to deviate unilaterally, as such deviations would lead to lower payoffs by failing to meet the required provision point, or by wasting non-needed contributions. There is a special case when both players contribute exactly 6 MUs ($x_i = 6, x_j = 6$). This allows them to reach the first provision point and each receive a payoff of 14 MUs. However, this strategy

does not hold as a Nash equilibrium considering that once the first provision point was reached, players always have an incentive to contribute more to reach the second provision point.

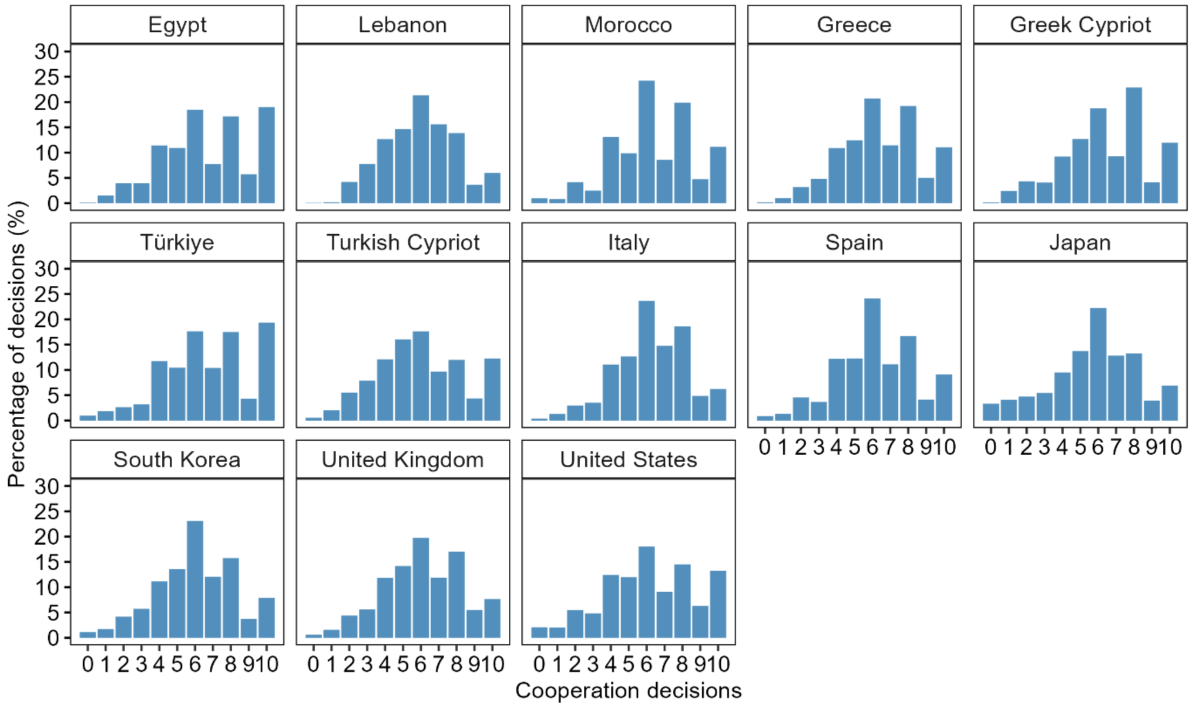

Figure S7a. Percentage of each cooperation decision (0-10) per society.

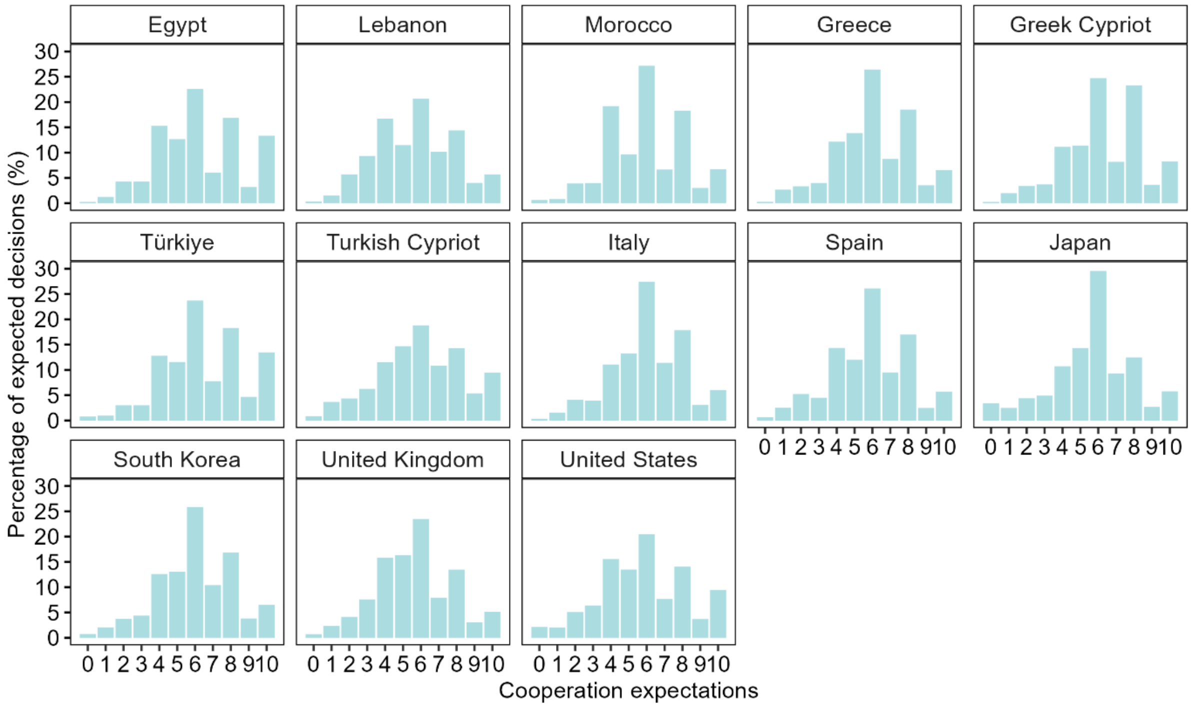

Figure S7b. Percentage of each expected cooperation decision from others (0-10) per society.

6. References

1. Willroth, E. C. & Atherton, O. E. Best laid plans: A guide to reporting preregistration deviations. *Adv. Methods Pract. Psychol. Sci.* **7**, 25152459231213802 (2024).
2. Muthen, L. K. & Muthén, B. O. Mplus (Version 8.10) [computer software]. *Los Angel. CA Muthén Muthén* (2023).
3. Hu, L. & Bentler, P. M. Cutoff criteria for fit indexes in covariance structure analysis: Conventional criteria versus new alternatives. *Struct. Equ. Model. Multidiscip. J.* **6**, 1–55 (1999).
4. Kline, R. B. *Principles and Practice of Structural Equation Modeling*. (Guilford Publications, 2023).
5. Asparouhov, T. & Muthén, B. *SRMR in Mplus. Technical Appendix*. (Los Angeles, CA: Muthén & Muthén., 2018).
6. Vignoles, V. L. *et al.* Are Mediterranean societies "cultures of honor?": Prevalence and implications of a cultural logic of honor across three world regions. *Pers. Soc. Psychol. Bull.* 01461672241295500 (2024) doi:10.1177/01461672241295500.
7. Różycka-Tran, J., Boski, P. & Wojciszke, B. Belief in a zero-sum game as a social axiom: A 37-nation study. *J. Cross-Cult. Psychol.* **46**, 525–548 (2015).
8. Thomson, R. *et al.* Relational mobility predicts social behaviors in 39 countries and is tied to historical farming and threat. *Proc. Natl. Acad. Sci. U. S. A.* **115**, 7521–7526 (2018).
9. Enders, C. K. & Tofighi, D. Centering predictor variables in cross-sectional multilevel models: A new look at an old issue. *Psychol. Methods* **12**, 121–138 (2007).
10. Sirola, N. & Pitesa, M. Economic downturns undermine workplace helping by promoting a zero-sum construal of success. *Acad. Manage. J.* **60**, 1339–1359 (2017).

11. Murray, D. R. & Schaller, M. Historical prevalence of infectious diseases within 230 geopolitical regions: A tool for investigating origins of culture. *J. Cross-Cult. Psychol.* **41**, 99–108 (2010).

12. Romano, A., Sutter, M., Liu, J. H., Yamagishi, T. & Balliet, D. National parochialism is ubiquitous across 42 nations around the world. *Nat. Commun.* **12**, 4456 (2021).

13. De Dreu, C. K. W., Gross, J., Fariña, A. & Ma, Y. Group cooperation, carrying-capacity stress, and intergroup conflict. *Trends Cogn. Sci.* **24**, 760–776 (2020).

14. Spadaro, G. *et al.* Cross-cultural variation in cooperation: A meta-analysis. *J. Pers. Soc. Psychol.* **123**, 1024–1088 (2022).

15. Flora, D. B. Your coefficient Alpha is probably wrong, but which coefficient Omega is right? A tutorial on using R to obtain better reliability estimates. *Adv. Methods Pract. Psychol. Sci.* **3**, 484–501 (2020).
