# Supplementary material for: Honour, competition and cooperation across 13 societies
Source: Nat Hum Behav. 2025 Sep 26;10(2):255–67. doi: 10.1038/s41562-025-02308-0 (PMC12932108; doi:10.1038/s41562-025-02308-0)
Supplement: Supplementary file 2 — Reporting Summary [file 41562_2025_2308_MOESM2_ESM.pdf]

nature portfolio

Corresponding author(s): Shuxian Jin

Last updated by author(s): Jul 27, 2025

Reporting Summary

Nature Portfolio wishes to improve the reproducibility of the work that we publish. This form provides structure for consistency and transparency in reporting. For further information on Nature Portfolio policies, see our Editorial Policies and the Editorial Policy Checklist.

Statistics

For all statistical analyses, confirm that the following items are present in the figure legend, table legend, main text, or Methods section.

n/a	Confirmed	
☐	☒	The exact sample size (*n*) for each experimental group/condition, given as a discrete number and unit of measurement
☐	☒	A statement on whether measurements were taken from distinct samples or whether the same sample was measured repeatedly
☐	☒	The statistical test(s) used AND whether they are one- or two-sided<br>*Only common tests should be described solely by name; describe more complex techniques in the Methods section.*
☐	☒	A description of all covariates tested
☒	☐	A description of any assumptions or corrections, such as tests of normality and adjustment for multiple comparisons
☐	☒	A full description of the statistical parameters including central tendency (e.g. means) or other basic estimates (e.g. regression coefficient) AND variation (e.g. standard deviation) or associated estimates of uncertainty (e.g. confidence intervals)
☐	☒	For null hypothesis testing, the test statistic (e.g. *F*, *t*, *r*) with confidence intervals, effect sizes, degrees of freedom and *P* value noted<br>*Give P values as exact values whenever suitable.*
☒	☐	For Bayesian analysis, information on the choice of priors and Markov chain Monte Carlo settings
☐	☒	For hierarchical and complex designs, identification of the appropriate level for tests and full reporting of outcomes
☐	☒	Estimates of effect sizes (e.g. Cohen's *d*, Pearson's *r*), indicating how they were calculated

Our web collection on statistics for biologists contains articles on many of the points above.

Software and code

Policy information about availability of computer code

Data collection	Individual responses were collected using the Qualtrics software (Version May 2023).
Data analysis	Data were analyzed using the software R (version 4.2.1). Confirmatory factor analyses were conducted using the software Mplus (version 8.10). The code used to analyse the data is publicly available at https://osf.io/3dscw/. The R code is also provided on the Code Ocean platform (https://doi.org/10.24433/CO.9371203.v1), allowing for a straightforward reproducible run.

For manuscripts utilizing custom algorithms or software that are central to the research but not yet described in published literature, software must be made available to editors and reviewers. We strongly encourage code deposition in a community repository (e.g. GitHub). See the Nature Portfolio guidelines for submitting code & software for further information.

Data

Policy information about availability of data

All manuscripts must include a data availability statement. This statement should provide the following information, where applicable:

- Accession codes, unique identifiers, or web links for publicly available datasets
- A description of any restrictions on data availability
- For clinical datasets or third party data, please ensure that the statement adheres to our policy

The datasets generated and analysed during the current study are publicly available at https://osf.io/3dscw/.

Research involving human participants, their data, or biological material

Policy information about studies with human participants or human data. See also policy information about sex, gender (identity/presentation), and sexual orientation and race, ethnicity and racism.

Reporting on sex and gender	Participants were asked to self-report their sex/gender (male; female; not listed, please specify) at the beginning of the study, following the provision of informed consent. Only those who self-identified as male or female were included in the data analysis. In surveys conducted in the Arabic (Egypt, Lebanon, Morocco), Greek (Greece, Greek Cypriot community), Japanese (Japan), Korean (South Korea), and Turkish (Türkiye, Turkish Cypriot community) languages, the terms "sex" and "gender" were translated using the same word. In the English (U.K., U.S.A.), Italian (Italy), and Spanish (Spain) versions of the survey, (the translation of) the term "sex" was used in the question.<br><br>Across these 13 societies, we consider this self-reported measure closer to the working definition of gender rather than a strictly binary concept of sex, provided on the Nature portfolio, because participants were always provided a third option (i.e., not listed, please specify) to indicate self-identifications beyond male and female.<br><br>We also manipulated the sex/gender information of the game partner (male vs. female vs. not provided) with whom the participants' decision would be paired if that game round was selected for game payment calculation. Across these 13 societies, we consider this manipulation to reflect partner gender information for the same reasons mentioned above.<br><br>Individual-level participant gender information is provided in the source data. Consent has been obtained for sharing de-identified individual-level data.
Reporting on race, ethnicity, or other socially relevant groupings	Participants were asked to self-report their ethnic and religious backgrounds at the end of the study. The categories provided for these demographic questions were adapted to each society by local collaborators to ensure that they reflected locally meaningful categories. Individual-level data on these variables are provided in the source data. In Table S35 of the Supplementary Information, we report the percentage of participants who self-identified as belonging to the major ethnic group in the respective society (see the column "% Majority"). These two variables were not used as control variables in the analysis.
Population characteristics	See "Behavioural & social sciences study design" section.
Recruitment	Participants were recruited through an online panel provider (Toluna, https://www.toluna.com/) including members of its third-party panel providers. As an exception, participants from Cyprus were recruited through a market research agency based in the Greek Cypriot community (CYMAR, https://www.cymar.com.cy/), and a research, analysis and consultancy organization based in the Turkish Cypriot community (Statica, https://staticacy.com/). See Table S35 of the Supplementary Information for more details about the panels. Participants in all 13 societies were compensated for their participation in the study, and also received additional payment based on their own and their paired game partner's decisions at the end of data collection in each society. To minimize self-selection bias, we did not set specific requirements for participation. The recruitment template included only general information about the estimated survey length and the compensation for participation.
Ethics oversight	This study was approved by the Sciences & Technology Cross-Schools Research Ethics Committee (C-REC) at the University of Sussex (ER/SJ468/1).

Note that full information on the approval of the study protocol must also be provided in the manuscript.

Field-specific reporting

Please select the one below that is the best fit for your research. If you are not sure, read the appropriate sections before making your selection.

☐ Life sciences ☒ Behavioural & social sciences ☐ Ecological, evolutionary & environmental sciences

For a reference copy of the document with all sections, see nature.com/documents/nr-reporting-summary-flat.pdf

Behavioural & social sciences study design

All studies must disclose on these points even when the disclosure is negative.

Study description	Quantitative data, experimental and correlational design
Research sample	This study involved participants from 13 societies, recruited from participant pools provided by panel providers, including Toluna and its third-party partners, CYMAR and Statica. The percentage of females in the final sample ranged from 48% to 53%, and participants' mean age ranged from 39.25 (SD = 12.83) to 41.56 (SD = 14.91) across the 13 societies. These panel providers were chosen because their samples are heterogeneous in terms of age, gender, and socio-economic background. Due to limited access to participants in the Greek and Turkish Cypriot communities through Toluna and its third-party partners, we collaborated with local research companies for data collection in these communities.
Sampling strategy	Participants were recruited from participant pools of the panel providers in each society. We stratified the participants by age and gender in each participant pool by setting quota groups for age (18-25, 26-35, 36-45, 46-55, 56+) and gender (male and female) at

the beginning of the Qualtrics surveys.

One of our main goals was to detect potential differences between societies in their level of competition and cooperation. A sensitivity power analysis indicated that a sample of 250 participants per society, with 80% power (α = .05), could detect an effect size of d = .25 between two societies. We thus aimed at recruiting 3,250 participants (~250 per society).

Data collection

This study involved anonymized online data collection. Participants either received an email invitation or accessed the Qualtrics survey link through panelist portals. Only participants in the Turkish Cypriot community completed the Qualtrics survey on a tablet provided by the research organization, in a separate room and alone, without the presence of the research representative who was blind to the study hypotheses and experimental conditions. Thus, across all societies, researchers could not influence the results knowing the hypotheses and the experimental conditions in advance.

Timing

Data collection in the Greek Cypriot community, managed by CYMAR: June 1, 2023 to June 9, 2023
Data collection in the Turkish Cypriot community, managed by Statica: June 21, 2023 to October 21, 2023
Data collection in the rest of the societies, managed by Toluna and its third-party panel provider: May 23, 2023 to June 13, 2023

Data exclusions

Several inclusion criteria were applied, resulting in the exclusion of a) 120 participants who were not born and located in the respective society, b) 24 participants who did not self-identify as male or female, c) 29 participants who failed the quality check question, and d) 112 participants who failed all four comprehension questions designed to assess participants' understanding of the contest game and step-level PGG rules. These criteria were established in consultation with panel providers regarding the availability of eligible samples in their participant pools before and during the data collection stage.

Non-participation

The response rate for each society, calculated as the final sample divided by the number of participants who agreed to participate and passed the quota group checks, was as follows: Egypt (75%), Greece (67%), Greek Cypriot community (80%), Italy (68%), Japan (54%), South Korea (71%), Lebanon (83%), Morocco (72%), Spain (47%), Türkiye (60%), Turkish Cypriot community (79%), United Kingdom (50%), and United States (33%).

Randomization

The design consisted of two counter-balanced within-participant treatments with type of game (i.e., contest game, step-level public goods game) and three randomized within-participant treatments related to the gender information of the pairing partner (i.e., male vs. female vs. gender information not provided). Thus, participants were not allocated into experimental groups. Counter-balancing and randomization were handled by Qualtrics. Both the order of the game and partner gender information were included in the analyses as control variables.

Reporting for specific materials, systems and methods

We require information from authors about some types of materials, experimental systems and methods used in many studies. Here, indicate whether each material, system or method listed is relevant to your study. If you are not sure if a list item applies to your research, read the appropriate section before selecting a response.

Materials & experimental systems

n/a	Involved in the study
☒	☐ Antibodies
☒	☐ Eukaryotic cell lines
☒	☐ Palaeontology and archaeology
☒	☐ Animals and other organisms
☒	☐ Clinical data
☒	☐ Dual use research of concern
☒	☐ Plants

Methods

n/a	Involved in the study
☒	☐ ChIP-seq
☒	☐ Flow cytometry
☒	☐ MRI-based neuroimaging

Plants

Seed stocks

Report on the source of all seed stocks or other plant material used. If applicable, state the seed stock centre and catalogue number. If plant specimens were collected from the field, describe the collection location, date and sampling procedures.

Novel plant genotypes

Describe the methods by which all novel plant genotypes were produced. This includes those generated by transgenic approaches, gene editing, chemical/radiation-based mutagenesis and hybridization. For transgenic lines, describe the transformation method, the number of independent lines analyzed and the generation upon which experiments were performed. For gene-edited lines, describe the editor used, the endogenous sequence targeted for editing, the targeting guide RNA sequence (if applicable) and how the editor was applied.

Authentication

Describe any authentication procedures for each seed stock used or novel genotype generated. Describe any experiments used to assess the effect of a mutation and, where applicable, how potential secondary effects (e.g. second site T-DNA insertions, mosiacism, off-target gene editing) were examined.
